# Discovery of a Novel Template, 7-Substituted 7-Deaza-4′-Thioadenosine Derivatives as Multi-Kinase Inhibitors

**DOI:** 10.3390/ph14121290

**Published:** 2021-12-10

**Authors:** Karishma K. Mashelkar, Woong Sub Byun, Hyejin Ko, Kisu Sung, Sushil K. Tripathi, Seungchan An, Yun A Yum, Jee Youn Kwon, Minjae Kim, Gibae Kim, Eun-Ji Kwon, Hyuk Woo Lee, Minsoo Noh, Sang Kook Lee, Lak Shin Jeong

**Affiliations:** 1 Research Institute of Pharmaceutical Sciences, College of Pharmacy, Seoul National University, Seoul 08826, Korea; karimashelkar@gmail.com (K.K.M.); sky_magic@naver.com (W.S.B.); hyejinko@snu.ac.kr (H.K.); stkiss@snu.ac.kr (K.S.); bipinsu.5@gmail.com (S.K.T.); ann081993@snu.ac.kr (S.A.); dua010@snu.ac.kr (Y.A.Y.); wldus5116@snu.ac.kr (J.Y.K.); mingae93@naver.com (M.K.); kgb_not_beer@naver.com (G.K.); Ejkwon226@gmail.com (E.-J.K.); minsoonoh@snu.ac.kr (M.N.); sklee61@snu.ac.kr (S.K.L.); 2 Future Medicine Co., Ltd., Seongnam 13449, Korea; hwlee@futuremedicine.co.kr

**Keywords:** 7-deaza-4′-thioadenosine derivatives, multi-kinase inhibitor, anticancer, nucleoside

## Abstract

The development of anticancer drugs remains challenging owing to the potential for drug resistance. The simultaneous inhibition of multiple targets involved in cancer could overcome resistance, and these agents would exhibit higher potency than single-target inhibitors. Protein kinases represent a promising target for the development of anticancer agents. As most multi-kinase inhibitors are heterocycles occupying only the hinge and hydrophobic region in the ATP binding site, we aimed to design multi-kinase inhibitors that would occupy the ribose pocket, along with the hinge and hydrophobic region, based on ATP-kinase interactions. Herein, we report the discovery of a novel 4′-thionucleoside template as a multi-kinase inhibitor with potent anticancer activity. The in vitro evaluation revealed a lead **1g** (7-acetylene-7-deaza-4′-thioadenosine) with potent anticancer activity, and marked inhibition of TRKA, CK1δ, and DYRK1A/1B kinases in the kinome scan assay. We believe that these findings will pave the way for developing anticancer drugs.

## 1. Introduction

Although cancer has been extensively investigated, drug resistance remains a major challenge in the clinical progress of anticancer drugs [1]. It is frequently responsible for treatment failure in patients with cancer undergoing monotherapy. Under these circumstances, a polypharmacological strategy may overcome the drug resistance crisis. The question then arises: How would it work? Cancer cells are dependent not only on a single oncogene but also on cells enclosing it. Therefore, inhibition of a single target produces mutations that promote cancer cell survival, in advanced cancers [2]. Rationally designed multi-target inhibitors that could hit more than one oncogenic target may surpass the effect mediated by single-target inhibitors, as they would obstruct cancer cell proliferation and, secondly, block the microenvironment that facilitates oncogenesis [3]. This would more comprehensively inhibit the pathway involved, simultaneously reducing the negative impact on tumor cells to acquire a resistance mutation. Accordingly, the synergistic effect of inhibiting multiple targets would induce less resistance and greater efficacy [3]. As cancer is a polygenic disease, it is worth noting that a single drug acting synchronously on multiple targets is advantageous over the combined use of individual single-target drugs [3]. Accordingly, smaller doses are required for simultaneous targets to produce desired effects as the molecule will be concurrently present in tissues (at the site of action). On the other hand, combination therapy complicates the dosing schedule, increases the risk of toxicity arising from drug-drug interactions, and negatively impacts patient adherence, contrary to multi-target drugs [4,5]. These well-established facts motivate our interest in the current study. Polypharmacology has become more appealing in recent years and is currently a hot topic in this field [6,7,8]. 

One main factor underlying cancer growth is the presence of kinase mutations. Protein kinases play a crucial role in cellular functions by mediating protein phosphorylation. These enzymes transfer the terminal phosphoryl group of adenosine triphosphate (ATP) to a protein substrate, ultimately resulting in processes, such as signal transduction, gene regulation, and metabolism. Therefore, dysregulation of kinases is often associated with several diseases, including cancer [9]. The protein kinase domain is the most common domain encoded by cancer genes [10] and is linked to cancer onset and progression [10,11]. Several multi-kinase inhibitors are currently in clinical use, indicating growing attention for multi-kinase inhibitors [12]; for example, multi-targeted receptor tyrosine kinase inhibitors, such as sorafenib—approved for the treatment of renal cell carcinoma (RCC) and hepatocellular carcinoma, and sunitinib—approved for the treatment of RCC and imatinib-resistant gastrointestinal stromal tumor have been developed [3,13,14]. Recently, the multi-target kinase inhibitor, entrectinib was approved by the Food and Drug Administration for the treatment of ROS1 (c-ros oncogene 1)-positive, metastatic non-small cell lung cancer and solid tumors with neurotrophic receptor tyrosine kinase (NTRK) fusions [15,16].

**Figure 1:**
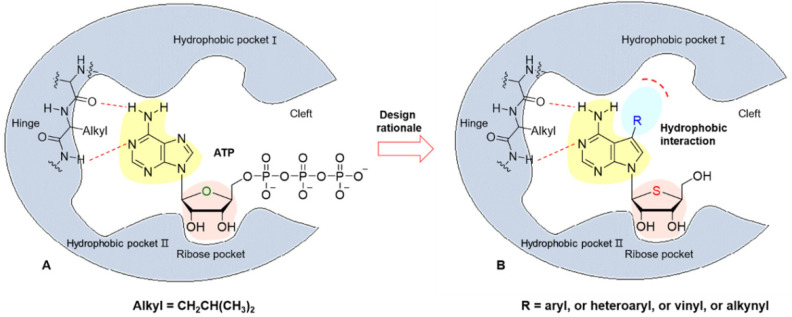
The rationale for the design of multi-kinase inhibitors with a nucleoside skeleton. (**A**) Schematic representation displaying catalytic domain of typical protein kinases binding ATP. Important interactions are highlighted with either colored or dashed lines, indicating hydrophobic interaction/hydrogen bonding. (**B**) Rational design of kinase inhibitors based on ATP-kinase interactions.

The majority of the kinase inhibitors are heterocycles, which are ATP-competitive [17,18,19], i.e., they act by competing with ATP to bind to the ATP-binding site of kinases, and therefore, block the phosphorylation process. The ATP binding site of protein kinases is illustrated in Figure 1A. The catalytic domain of all protein kinases encompasses two lobes, linked by a flexible hinge region. ATP binds to the cleft, between the two lobes, a highly conserved catalytic structure in protein kinases [20], where the transfer of γ-phosphate of ATP to protein substrate is catalyzed by kinases in their active DFG-in conformation. The adenine ring from ATP forms two hydrogen bonds with the amino acids in the hinge region [21]. The ATP pocket also contains the unoccupied hydrophobic pockets and a hydrophilic ribose region. Most of the ATP-competitive inhibitors known, commonly occupy the hinge and hydrophobic regions I, II [22,23] but rarely the ribose pocket. Nevertheless, it is worthy to note that, occupying the ribose region results in improved binding towards kinases as demonstrated by Gandin et al. [23]. Since ATP binding site is conserved in protein kinases, it could be a challenging task to design selective multi-kinase inhibitors [24] as it can often lead to off-target interactions [22,25]. It may be advantageous in treating polygenic diseases like cancer, where polypharmacological agents are more effective [3]. However, to maintain the safety profile of a multi-kinase inhibitor, only specific kinases should be targeted [22]. It would be of great importance to find out the combination of kinases whose inhibition would result in therapeutic benefits without unwanted side effects [26,27]. The state-of-the-art of kinase inhibitors in human trials have been provided by Klaeger et al. [28]. 

In the present study, we aimed to design multi-kinase inhibitors that would interact with the hinge region, hydrophobic pocket I, also known as buried region adjacent to the hinge region and ribose pocket at the same time. The interactions with the hydrophobic pocket have frequently been utilized to achieve inhibitor selectivity over kinases [23]. On this basis, we attempted to design novel kinase inhibitors with a nucleoside skeleton as it’s an ATP-mimic by modifying the hydrophobic residue (R), based on ATP-kinase interactions, as illustrated in Figure 1B. These compounds are expected to simultaneously inhibit several kinases, given the sequence and structural homology among the ATP binding sites of kinases [20]. Nevertheless, we wanted to determine the most suitable substituent for the hydrophobic pocket which is not occupied by ATP, whether acting as a pharmacophore for selective kinase inhibition. To achieve this goal, we selected a 7-deazaadenine scaffold, as it serves as a good template for functionalization at the 7-position to occupy the adjacent hydrophobic pocket, thereby enhancing the interactions with the kinase. It is interesting to note how a subtle structural variation in the nucleobase of adenosine exerts cytotoxic biological properties, as demonstrated by a natural product tubercidin (7-deazaadenosine) [29,30]. The 7-substituted-tubercidin analogs also showed very interesting anti-cancer activity [31]. The sugar pocket is predominantly hydrophilic and conserved in most protein kinases. It is well known that the bioisosteric replacement of oxygen with sulfur on furanose imparts chemotherapeutic properties to its respective sulfur analog [32,33,34,35] with metabolic stability [36]. Like the ribose ring in ATP, the hydrophilic polar hydroxyl group of the 4-thiosugar moiety will form a hydrogen bond with the sugar region enabling the molecule to fit in it, resulting in enhanced binding [23]. 

All the synthesized compounds were evaluated for their anticancer activity by employing a sulforhodamine B (SRB) colorimetric assay and the most potent compound **1g** (7-acetylene-7-deaza-4′-thioadenosine) was screened for kinase panel assay. Accordingly, compound **1g** was found to inhibit TRKA (neurotrophic tyrosine receptor kinase 1, NTRK1), DYRK1A/1B (dual specificity tyrosine-phosphorylation-regulated kinase 1A and 1B), and CK1δ (casein kinase 1 delta, CSNK1D) kinases, reportedly associated with overexpression in cancer cells [11,37,38,39]. To the best of our knowledge, we report for the first time the design and synthesis of 7-substituted 7-deaza-4′-thionucleoside analogs that are supposed to simultaneously occupy hinge, hydrophobic, and ribose regions and their structure-activity relationship as a multi-kinase inhibitor against TRKA, DYRK1A, DYRK1B, and CK1δ with potent anticancer activity. 

## 2. Results and Discussion

### 2.1. Chemistry

**Figure 2:**
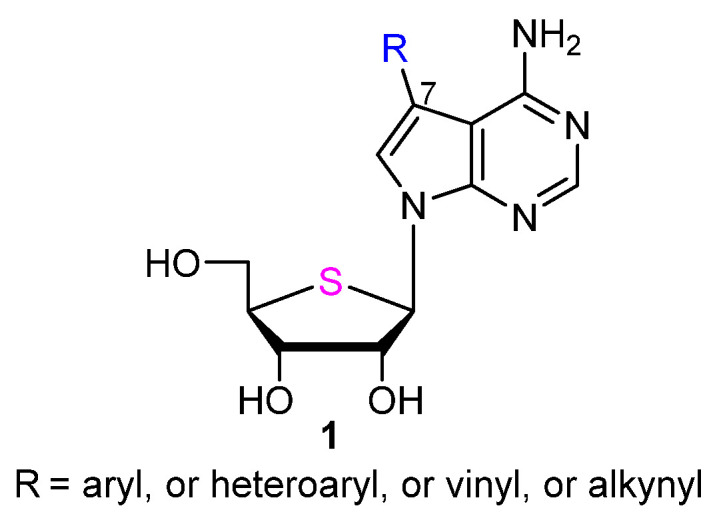
The structures of target nucleoside analogs were modified at the C7 position.

The structures of synthesized compounds are represented in Figure 2. 

**Scheme 1:**
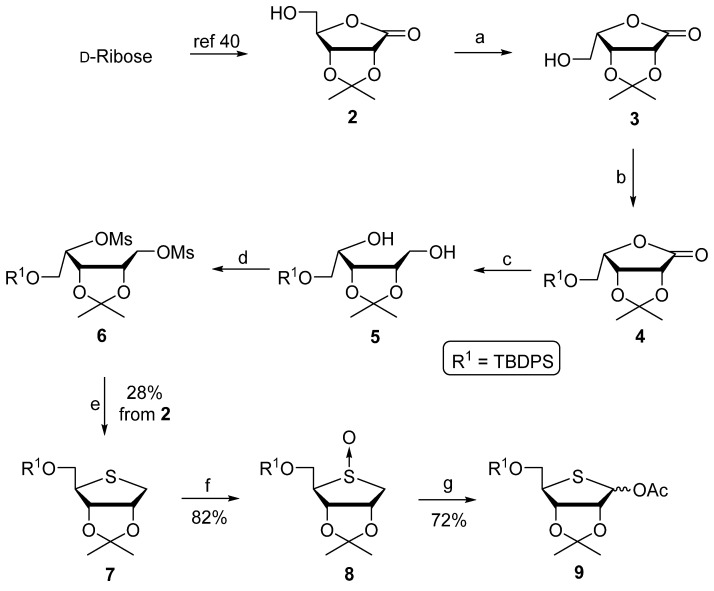
Synthesis of glycosyl donor **9** from D-ribose. Reagents and conditions: (a) (i) MsCl, pyridine, 0 °C, 4 h; (ii) KOH, H_2_O, rt, 12 h; (b) TBDPSCl, imidazole, DMAP, CH_2_Cl_2_, 0 °C to rt, 12 h; (c) NaBH_4_, THF/MeOH, 0 °C to rt, 2 h; (d) MsCl, Et_3_N, CH_2_Cl_2_, 0 °C to rt, 2 h; (e) Na_2_S^.^9H_2_O, DMF, 90 °C, 15 h; (f) *m*CPBA, CH_2_Cl_2_, −78 °C, 45 min; (g) acetic anhydride, 110 °C, 4 h.

As shown in Scheme 1, to synthesize the final nucleoside **1**, we first synthesized the glycosyl donor **9** from commercially available D-ribose. 

D-Ribose was converted to 2,3-*O*-isopropylidene-D-ribonic γ-lactone (**2**) using the two steps protocol as described previously [40]. First, D-ribose was converted to D-ribonolactone using bromine/water in the presence of potassium carbonate and later treated with acetone in the presence of a catalytic amount of concentrated sulfuric acid at room temperature to afford **2**. Following the reported general protocol [41] for thiosugar **7**, the inversion of configuration at the C4 chiral center of D-ribonolactone was achieved by treating **2** initially with mesyl chloride and subjecting it to base hydrolysis using aqueous potassium hydroxide solution to give **3** with inverted stereochemistry. Protection of the C5-hydroxyl of **3** with *tert*-butyldiphenylsilyl (TBDPS), followed by reduction of the resulting compound **4** with NaBH_4_, afforded diol **5**. Sulfur heterocyclization was performed by converting **5** to dimesylate **6**, immediately reacted with sodium sulfide nonahydrate at 90 °C to give **7** at a 28% overall yield from **2**. Compound **7** was subjected to *m*CPBA oxidation at −78 °C to give sulfoxide **8** (82% yield). Pummerer rearrangement of **8** occurred upon heating with acetic anhydride to afford glycosyl donor **9** as a 1:1.6 α/β anomeric mixture.

**Scheme 2:**
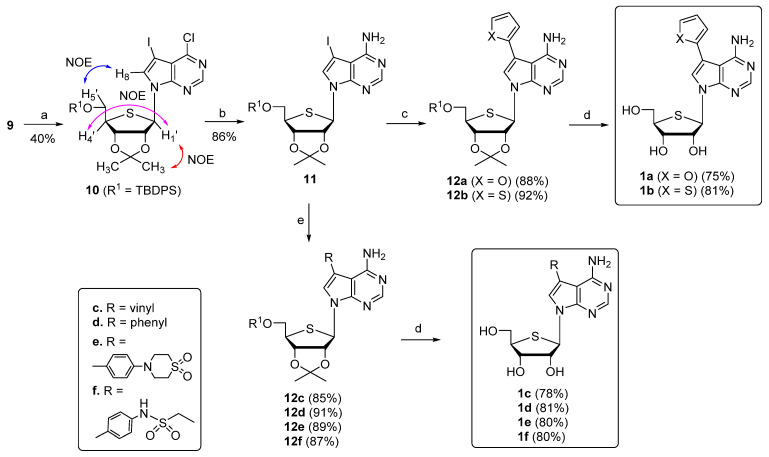
Synthesis of 7-substituted 7-deaza-4′-thioadenosine derivatives **1a–f**. Reagents and conditions: (a) 7-Deaza-7-iodo-6-chloropurine, BSA, TMSOTf, CH_3_CN, rt to 80 °C, 1 h; (b) NH_3_/*tert*-BuOH, 90 °C, 12 h; (c) corresponding 2-tributylstannylheteroaryl, PdCl_2_(PPh_3_)_2_, THF, MW, 70 °C, 1 h; (d) 50% TFA/H_2_O, THF, rt, 12 h; (e) corresponding boronic ester, PdCl_2_(PPh_3_)_2_, Na_2_CO_3_, DMF/H_2_O, MW, 70 °C, 1 h.

The glycosyl donor **9** was then condensed with silylated 7-deaza-7-iodo-6-chloropurine under heating at 80 °C for 1 h in the presence of a Lewis acid, TMSOTf, to afford the desired β-stereoisomer **10** as a single stereoisomer (40% yield; Scheme 2); however, the same reaction at room temperature failed to afford the desired product. The β configuration of condensed nucleoside **10** was easily determined by 2D NOESY experiments. The NOESY spectrum revealed a correlation between 1′-H and 4′-H, as well as between 1′-H and one of the two methyl groups of the acetonide group. A correlation between 5′-H and H-8 was also observed, confirming the presence of the β-D-anomer (see the Supporting Information). Ammonolysis of **10** in *tert*-butanolic ammonia at 90 °C produced key intermediate **11**, which was ready for functionalization with hydrophobic groups at the C7 position via palladium-catalyzed cross-coupling reactions. Pd-catalyzed Stille coupling of 7-iodo derivative **11** with 2-tributylstannylfuran and 2-tributylstannylthiophene in the presence of PdCl_2_(PPh_3_)_2_ yielded 7-furanyl and 7-thiofuranyl derivatives **12a** and **12b**, respectively. Removal of acetonides of **12a** and **12b** with 50% aqueous trifluoroacetic acid (TFA) afforded the final nucleosides, **1a**, and **1b**, respectively. 

Further Suzuki coupling reactions were performed to introduce other hydrophobic groups, such as vinyl, phenyl, and 4-substituted-phenyl to **11**. Coupling of **11** with vinyl boronic ester in the presence of PdCl_2_(PPh_3_)_2_ in DMF/H_2_O gave **12c** in 85% yield. In this Suzuki coupling reaction, water was used as a co-solvent to avoid side reactions, resulting from Heck coupling [42]. The desired Suzuki coupled product was obtained as a single product. Similar Suzuki reactions of **11** with phenyl and 4-substituted phenyl boronic esters afforded 7-phenyl and 7-(4-substituted)phenyl derivatives, **12d**–**f**. Treatment of **12c**–**f** with 50% aqueous TFA yielded the final nucleosides **1c**–**f**, respectively.

**Scheme 3:**
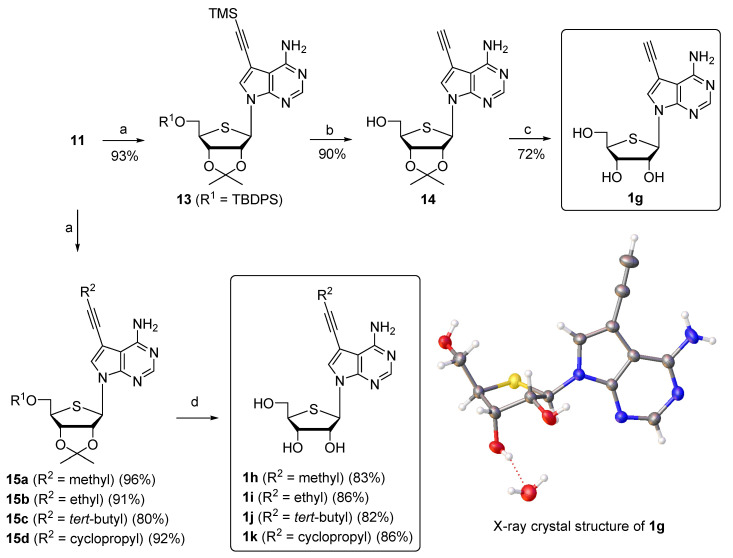
Synthesis of 7-substituted 7-deaza-4′-thioadenosine derivatives **1g**–**k**. Reagents and conditions: (a) corresponding alkyne, PdCl_2_(PPh_3_)_2_, CuI, Et_3_N, DMF, MW, 50 °C, 1 h; (b) 1 M TBAF, THF, rt, 40 min; (c) 2 N HCl, THF, rt, 15 h; (d) 50% TFA/H_2_O, THF, rt, 12 h.

Next, to introduce other linear hydrophobic groups, such as acetylene, Sonogashira coupling was employed, as shown in Scheme 3. Treatment of **11** with trimethylsilyl acetylene in the presence of a palladium catalyst and copper iodide afforded **13** (93% yield). Removal of the silyl groups of **13** with 1 M TBAF solution in THF afforded **14**, which was treated with 2 N HCl to yield the 7-acetylene derivative **1g**. The molecular structure of **1g** was confirmed by a single X-ray crystal analysis (CCDC 1575257); further evidence supporting the β-configuration is provided in the Supporting Information [43]. Several 2-substituted acetylene analogs **15a**–**d** were also synthesized from **11** by employing the same Sonogashira coupling conditions. The final propylene, butylene, *tert*-butyl acetylene, and cyclopropyl acetylene analogs **1h**–**k** were obtained by treating **15a**–**d** with 50% aqueous TFA at room temperature.

### 2.2. Biological Evaluation

#### 2.2.1. Antiproliferative Activity

All synthesized compounds **1a**–**k** were evaluated for their antiproliferative activity against six different cancer cell lines, including human lung (A549), colon (HCT116), breast (MDA-MB-231), liver (SK-HEP-1), stomach (SNU638), and prostate (PC-3) cancer cells, using SRB colorimetric assay [44,45]. As demonstrated in Table 1, compounds **1a** and **1b** possessing furanyl and thiofuranyl moieties at the 7-position, respectively, exhibited moderate antiproliferative activity. Compound **1c**, with a vinyl substituent at the 7-position, displayed potent anticancer activity, whereas bulky groups, such as phenyl (**1d**) and substituted phenyls (**1e** and **1f**) showed low to no anticancer activity. In contrast, the linear acetylene moiety (**1g**) at the 7-position exhibited excellent anticancer activity in the nanomolar range. Surprisingly, 2-substituted acetylene derivatives **1h**–**k** abolished antiproliferative activity. This result demonstrates that a small and linear hydrophobic group, such as acetylene is necessary for potent anticancer activity. Since compound **1g** exhibited the most potent antiproliferative activity against cancer cells (IC_50_ = 0.004–0.06 μM), the antiproliferative activity of **1g** against normal cells was additionally evaluated in primary dermal fibroblast cells. Compound **1g** also showed considerable growth inhibition in cultured human normal dermal fibroblast cells (IC_50_ = 0.15 μM). Although rather toxic, compound **1g** seems to be more potent in the antiproliferative activity of cancer cells than normal cells.

#### 2.2.2. Kinome Scan Profile

**Figure 3:**
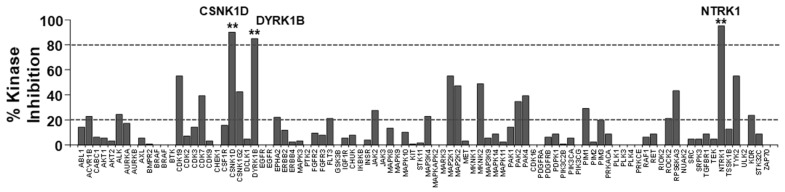
Kinase inhibition profile of compound **1g**. Kinome scan assays were performed for compound **1g** (1 μM) against 96 kinases. **—strong inhibition of target kinases (<20% activity remaining).

To characterize the kinase inhibition profile of the most potent compound **1g**, it was profiled against a panel of 96 kinases at a concentration of 1 μM (Figure 3). The results revealed that compound **1g** exhibited strong inhibitory activities against four kinases at 1 μM (<20% activity remaining), i.e., TRKA (NTRK1), DYRK1B, and CK1δ (CSNK1D) among the panel (see the Supporting Information for tabular representation of kinome scan data, Table S1). Since compound **1g** showed strong inhibition of DYRK1B, it was evaluated for its isoform, DYRK1A inhibition. Compound **1g** displayed potent inhibition of DYRK1A (IC_50_ = 43 nM, see the Supporting information, Table S2). These kinases are reportedly involved in cancer initiation and progression [11,37,38,39] and represent promising targets for cancer therapy. Based on this result, it could be inferred that the hydrophobic pocket in the ATP binding site of these four kinases could accommodate only small and linear hydrophobic groups such as acetylene for kinase inhibition. Next, the concentration-dependent inhibitory activities of compounds **1a**–**k** were investigated and the half-maximal inhibitory concentration (IC_50_) was determined against four kinases, TRKA, DYRK1A, DYRK1B, and CK1δ (see the Supporting information, Table S2). The kinase inhibition trend observed for **1a**–**k** was almost similar to that of their antiproliferative activity. In general, compounds **1a**, **1b**, and **1g** exhibited excellent kinase inhibition activities, whereas compounds **1c** and **1d** exhibited moderate kinase inhibition. Among compounds tested, compounds **1a** and **1b** with 7-furanyl and 7-thiofuranyl substituents, respectively showed excellent kinase inhibition activity against NTRK1, whereas compounds **1a** and **1g** with 7-furanyl and 7-acetylene substituents, respectively showed excellent kinase inhibition activity against DYRK1A and DYRK1B. Compounds **1b** and **1d** with 7-thiofuranyl and 7-phenyl substituents, respectively showed excellent inhibition against CSNK1D. However, sterically demanding compounds **1e** and **1f** and the 2-substituted acetylene derivatives **1h**–**k** demonstrated weak to no inhibitory activity. Compound **1g** showed the best antiproliferative activity, but compound **1a** was discovered as the best inhibitory compound against the above-mentioned four kinases, indicating that the anti-cancer effect of **1g** might occur by unexpected mode of action.

#### 2.2.3. Antiproliferative Activity against KM12 and ACHN Cell Lines

The effect of **1g** on cell growth was also determined in KM12, a colon cancer cell line that highly expresses NTRK1/2/3 and DYRK3. Compared to doxorubicin (IC_50_ = 0.12 μM), compound **1g** (IC_50_ = 0.07 μM) showed marked activity against the cell growth of KM12. Likewise, IC_50_ of **1g** on cell growth of ACHN, a renal cancer cell line, expressing CK1δ and DYRK2 was approximately 0.04 μM, when compared with 0.04 μM of doxorubicin, suggesting the highly effective and selective anti-proliferative activity of **1g** in cancer cell lines expressing NTRK, DYRK2 or CK1δ; However, we can’t exclude the possibility that anti-cancer phenotype might be driven through some other mechanism.

#### 2.2.4. Metabolic Stability and CYP Inhibition

Based on anticancer and kinase inhibition data, compound **1g** was examined for its microsomal stability and CYP isozyme inhibition in vitro (Table 2). Compound **1g** was metabolically stable in human liver microsomes. Moreover, it showed no CYP isozyme inhibition against the five major drug-metabolizing cytochrome P450 isozymes.

### 2.3. Docking Analysis

**Figure 4:**
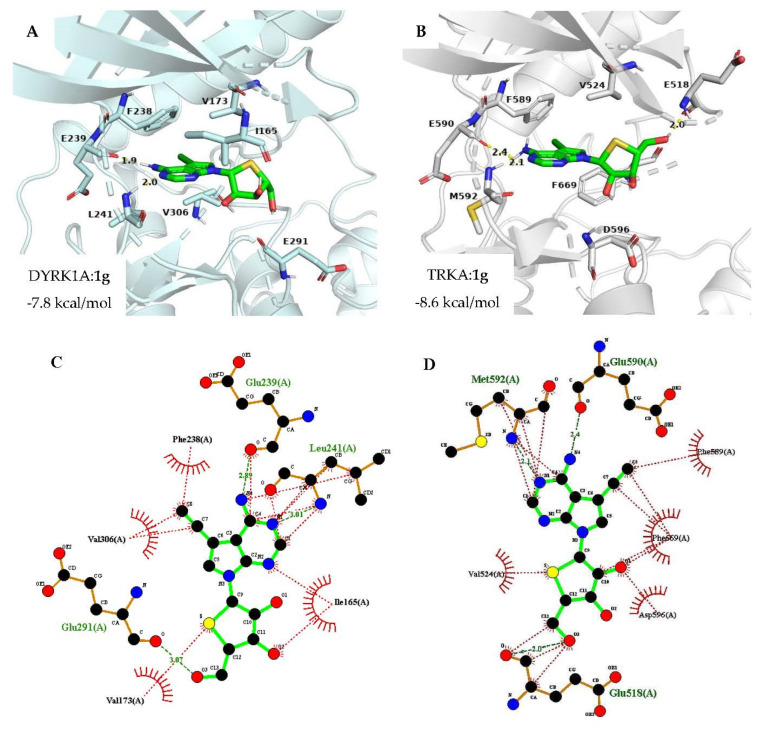
(**A**) Proposed binding mode of **1g** (represented in green) in the ATP binding site of (**A**) DYRK1A (represented in pale cyan; PDB ID: 7A51). (**B**) TRKA (represented in gray; PDB ID: 5JFV). Hydrogen bonding is represented as yellow dashed lines. Non-polar hydrogen atoms are omitted. (**B**) 2D interaction diagram of **1g** (represented in green) with (**C**) DYRK1A amino acid residues. (**D**) TRKA amino acid residues.

Next, to justify the rationale of this design strategy, the ligand-bound DYRK1A (PDB ID: 7A51) [46] and TRKA (PDB ID: 5JFV) [47] crystal structures were used for the molecular docking study (Figure 4). The docked pose of compound **1g** into the ATP binding site of DYRK1A and TRKA, as depicted in Figure 4A and 4B respectively, revealed that the purine ring formed two key hydrogen bonds with hinge residue Leu 241, and Glu 239 of DYRK1A, whereas in the case of TRKA the purine ring of **1g** formed hydrogen bonding with Met 592 and Glu 590, in a manner comparable with that of ATP. Furthermore, hydrogen bonding interaction was seen between 5′-OH of **1g** and Glu 291 of DYRK1A (Figure 4C). Similarly, hydrogen bonding interaction between 5′-OH of **1g** and Glu 518 of TRKA was observed. In addition, the acetylene moiety occupied the hydrophobic pocket as supposed and displayed hydrophobic interactions with gatekeeper residue, Phe 238 and Val 306 of DYRK1A (Figure 4C). While, in the TRKA: **1g** docked pose acetylene was found to form hydrophobic interactions with the gatekeeper residue, Phe 589 and Phe 669 (Figure 4D). Additionally, a hydrophobic interaction of 4-thio of the sugar moiety with Val 173 and Val 524 of DYRK1A and TRKA, respectively was observed. Thus, it can be concluded that compound **1g** fits markedly well in the ATP binding sites of DYRK1A and TRKA, thus providing a new scaffold that inhibits the activity of these enzymes.

## 3. Materials and Methods

### 3.1. General Methods

Proton (^1^H) and carbon (^13^C) NMR spectra were recorded on a JEOL JNM-GCX (400/100 MHz), Bruker AMX-500 (500/125 MHz), or JEOL JNM-ECA 600 (600/150 MHz) spectrometer. Chemical shifts are given in parts per million (*δ*), calibrated to the solvent peak, and coupling constants (*J*) in hertz (Hz). High-resolution mass (HRMS) measurements were recorded on a Thermo LCQ XP instrument. UV spectra were recorded in methanol on a U-3000 made by Hitachi. Optical rotations were measured on Jasco III in an appropriate solvent and [α]^25^_D_ values are given in 10^−1^ deg cm^2^ g^−1^. Melting points were determined on a Barnstead electrothermal 9100 instrument and are uncorrected. Microwave-assisted reactions were conducted in Biotage Initiator+ US/JPN (part no. 356007) microwave reactor. The TLC spots were examined under ultraviolet light at 254 nm and further visualized by *p*-anisaldehyde or phosphomolybdic acid stain solution. Column chromatography was performed using silica gel (Kieselgel 60, 70–230 mesh, Merck). The purity of all tested compounds was determined by high-performance liquid chromatography (HPLC) analysis, confirming ≥95% purity. 

### 3.2. Chemical Synthesis 

#### 3.2.1. (3aR,6S,6aR)-6-(Hydroxymethyl)-2,2-dimethyldihydrofuro[3,4-d][1,3]dioxol-4(3aH)-one (**3**) 

To an ice-cooled solution of **2** (50 g, 0.265 mol) in pyridine (330 mL), methanesulfonyl chloride (52.34 g, 0.45 mol) was dropwise added under a nitrogen atmosphere and the solution was stirred at room temperature for 4 h. The reaction was quenched with slow addition of saturated aqueous NaHCO_3_ (520 mL) with stirring until no effervescence and extracted with dichloromethane (2 L). The combined organic layer was washed successively with water and brine, dried (MgSO_4_), and concentrated in vacuo below 25 °C to give the crude product. To this crude mesylate, a solution of potassium hydroxide (36.74 g, 0.65 mol) in water (250 mL) was added using a dropping funnel, maintaining the temperature below 30 °C. This reaction mixture was stirred at room temperature for 12 h and then adjusted to pH 3.0 to 4.0 by adding 3 M hydrochloric acid (260 mL). The acidic solution was concentrated under reduced pressure to afford a solid mass. The solid mass was triturated with acetone (2 L) and heated to 50 °C for 30 min. The acetone was decanted, dried over anhydrous MgSO_4_, and filtered. The filtrate was concentrated to obtain crude **3** (*R*_f_ = 0.45, TLC eluent = CH_2_Cl_2_/MeOH, 19:1). 

#### 3.2.2. (3aR,6S,6aR)-6-(((*tert*-butyldiphenylsilyl)oxy)methyl)-2,2-dimethyldihydrofuro[3,4-d][1,3]dioxol-4(3aH)-one (**4**) 

To a solution of **3** in methylene chloride (240 mL), imidazole (8.78 g, 129 mmol) was added, followed by dropwise addition of *tert*-butyldiphenylsilyl chloride (26 g, 94.26 mmol) at 0 °C. After being stirred at room temperature for 12 h, the reaction mixture was partitioned between methylene chloride (3 × 600 mL) and water (760 mL). The layers were separated and the combined organic layer was dried (MgSO_4_), filtered, and evaporated to give crude **4** (*R*_f_ = 0.50, TLC eluent = hexane/ethyl acetate, 4:1).

#### 3.2.3. (S)-2-((*tert*-butyldiphenylsilyl)oxy)-1-((4S,5R)-5-(hydroxymethyl)-2,2-dimethyl-1,3-dioxolan-4-yl)ethan-1-ol (**5**)

The crude **4** was dissolved in THF-MeOH (285 mL-54 mL) and to this, sodium borohydride (14.64 g, 387.07 mmol) was added portion wise at 0 °C. After stirring for 2 h at room temperature, the reaction mixture was quenched with glacial acetic acid (26 mL) and evaporated. The residue was diluted with 20% aqueous potassium sodium tartrate (500 mL) and the aqueous layer was extracted with ethyl acetate (3 × 600 mL). The organic layer was washed with brine, dried over MgSO_4_, and evaporated to obtain crude **5** (*R*_f_ = 0.50, TLC eluent = hexane/ethyl acetate, 3:2).

#### 3.2.4. (S)-2-((*tert*-butyldiphenylsilyl)oxy)-1-((4R,5R)-2,2-dimethyl-5-(((methylsulfonyl)oxy)methyl)-1,3-dioxolan-4-yl)ethyl Methanesulfonate (**6**)

To a solution of **5** in methylene chloride (277 mL), 4-dimethylaminopyridine (0.36 g, 2.96 mmol) and triethylamine (106.40 g, 1051.53 mmol) were added and the solution was cooled to 0 °C. To this methanesulfonyl chloride (59.11 g, 516.09 mmol) was added dropwise and the reaction mixture was stirred at room temperature for 2 h. The reaction was quenched with saturated aqueous NaHCO_3_ (300 mL) until no effervescence and extracted with methylene chloride (3 × 520 mL). The combined organic layer was washed with brine (150 mL), dried (MgSO_4_), and passed through silica to remove any inorganic impurities and evaporated below 25 °C to give crude di-*O*-mesylate **6** (*R*_f_ = 0.55, TLC eluent = hexane/ethyl acetate, 3:2).

#### 3.2.5. *tert*-Butyl(((3aS,4R,6aR)-2,2-dimethyltetrahydrothieno[3,4-d][1,3]dioxol-4 yl)methoxy)diphenylsilane (**7**)

To a stirred solution of **6** in DMF (1.5 L) crushed Na_2_S⋅9H_2_O (43.37g, 180.60 mmol) was added and the reaction mixture was transferred to a preheated bath at 90 °C. After being stirred for 15 h, the reaction mixture was cooled to room temperature and quenched with water (1 L). The aqueous layer was extracted with n-hexane (3 × 1 L). The organic layer was combined, washed with brine, dried over anhydrous MgSO_4_, and concentrated. The residue was purified by column chromatography (silica gel, hexane/ethyl acetate, 19:1) to afford **7** (31.6 g, 28% in 5 steps) as pale yellow syrup; [α]^25^_D_ 45.17 (*c* 14.5, CH_3_OH); UV (CH_3_OH) *λ*_max_ 264.94 nm; ^1^H NMR (CDCl_3_, 500 MHz): *δ* 7.67–7.63 (m, 4H), 7.42–7.35 (m, 6H), 4.79 (d, *J* = 1.7 Hz, 2H), 3.76 (dd, *J* = 10.6, 4.9 Hz, 1H), 3.60 (dd, *J* = 10.6, 6.8 Hz, 1H), 3.38–3.35 (m, 1H), 3.13–3.10 (m, 1H), 2.82 (merged dd, *J*_1_ = *J*_2_ = 12.6 Hz, 1H), 1.50 (s, 3H), 1.30 (s, 3H), 1.04 (s, 9H); ^13^C NMR (CDCl_3_, 125 MHz): *δ* 135.56, 135.55, 133.0, 132.9, 129.8, 129.7, 127.7, 110.6, 85.9, 83.7, 65.9, 55.4, 38.3, 26.8, 26.4, 24.5, 19.1; HRMS (ESI-Q-TOF) *m*/*z* [M + NH_4_]^+^ for C_24_H_36_NO_3_SSi calculated 446.218, found 446.2175.

#### 3.2.6. (3aS,4R,5S,6aR)-4-(((*tert*-butyldiphenylsilyl)oxy)methyl)-2,2-dimethyltetrahydrothieno[3,4-d][1,3]dioxole 5-oxide (**8**)

A solution of 3-chloroperbenzoic acid (5.56 g, 32.25 mmol) in methylene chloride (110 mL) was added dropwise to a stirred solution of **7** (27.65 g, 64.50 mmol) in methylene chloride (110 mL) at −78 °C under a nitrogen atmosphere and stirred at the same temperature for 45 min. The reaction mixture was quenched with saturated NaHCO_3_ solution and extracted with methylene chloride (3 × 650 mL). The combined organic layer was washed with brine, dried over MgSO_4_, evaporated, and the residue was purified by silica gel column chromatography (hexane/ethyl acetate, 2:3) to afford **8** (23 g, 82%) as a colorless syrup; [α]^25^_D_ 17.87 (*c* 3.75, CH_3_OH); UV (CH_3_OH) *λ*_max_ 264.94 nm; ^1^H NMR (CDCl_3_, 500 MHz): *δ* 7.59–7.54 (m, 4H), 7.46–7.43 (m, 2H), 7.40–7.37 (m, 4H), 5.21 (t, *J* = 5.5 Hz, 1H), 4.97 (d, *J* = 5.3 Hz, 1H), 4.09 (dd, *J* = 11.2, 2.5 Hz, 1H), 3.82 (dd, *J* = 11.2, 3.3 Hz, 1H), 3.58 (s, 1H), 3.39 (merged dd, *J*_1_ = *J*_2_ = 14.8 Hz, 1H), 3.25 (dd, *J* = 14.8, 6.0 Hz, 1H), 1.56 (s, 3H), 1.33 (s, 3H), 1.01 (s, 9H); ^13^C NMR (CDCl_3_, 125 MHz): *δ* 135.4, 135.3, 131.7, 131.6, 130.1, 130.0, 127.9, 127.8, 112.2, 86.3, 84.7, 74.7, 61.7, 58.3, 26.7, 26.6, 24.3, 18.9; HRMS (ESI-Q-TOF) *m*/*z* [M + H]^+^ for C_24_H_33_O_4_SSi calculated 445.1863, found 445.1862.

#### 3.2.7. (3aR,6R,6aS)-6-(((*tert*-butyldiphenylsilyl)oxy)methyl)-2,2-dimethyltetrahydrothieno[3,4-d][1,3]dioxol-4-yl acetate (**9**)

A solution of **8** (16 g, 35.98 mmol) in acetic anhydride (142 mL) was transferred to a preheated bath at 110 °C and stirred at the same temperature for 4 h. After concentration under reduced pressure, the residue was neutralized with aqueous sat. NaHCO_3_ until pH 7.0 and stirred for 15 min. To the solution brine was added and extracted with ethyl acetate (3 × 600 mL). The organic layer was combined and washed with brine, dried (MgSO_4_), filtered, and evaporated under reduced pressure. The crude residue obtained was purified by silica gel column chromatography (hexane/ethyl acetate, 19:1) to give **9** (12.6 g, 72%) as a colorless syrup: 1:1.6 α/β mixture of anomers; UV (CH_3_OH) *λ*_max_ 259.85 nm; ^1^H NMR (CDCl_3_, 500 MHz): *δ* 7.70–7.62 (m, 6H), 7.43–7.40 (m, 3H), 7.39–7.36 (m, 7H), 6.09 (d, *J* = 5.3 Hz, 0.4H), 5.90 (s, 1H), 4.99 (d, *J* = 5.4 Hz, 1H), 4.82–4.80 (m, 0.6H), 4.64–4.61 (m, 1.5H), 3.82–3.80 (m, 1H), 3.77–3.72 (m, 1.6H), 3.60–3.52 (m, 2H), 2.13 (s, 1.4H), 1.81 (s, 3H), 1.53 (s, 1.9H), 1.48 (s, 3H), 1.30 (s, 1.6H), 1.28 (s, 3H), 1.06 (s, 9H), 1.04 (s, 5H); HRMS (ESI-Q-TOF) *m*/*z* [M + Na]^+^ for C_26_H_34_NaO_5_SSi calculated 509.1788, found 509.1791.

#### 3.2.8. 7-((3aR,4R,6R,6aS)-6-(((*tert*-butyldiphenylsilyl)oxy)methyl)-2,2-dimethyltetrahydrothieno[3,4-d][1,3]dioxol-4-yl)-4-chloro-5-iodo-7H-pyrrolo[2,3-d]pyrimidine (**10**)

*N*,*O*-Bis(trimethylsilyl)acetamide (BSA, 2.5 mL, 10.27 mmol) was added to a stirred suspension of 4-chloro-5-iodo-7*H*-pyrrolo[2,3-d]pyrimidine (2.6 g, 9.33 mmol) in anhydrous acetonitrile (67 mL) under nitrogen atmosphere. The resulting suspension was stirred at room temperature for 10 min until a homogeneous solution was obtained. To this clear solution a solution of **9** (5 g, 10.27 mmol) in anhydrous acetonitrile (50 mL) were added followed by dropwise addition of trimethylsilyl trifluoromethanesulfonate (1.5 mL, 8.40 mmol). The reaction mixture was stirred at room temperature for 15 min before transferring it to a preheated bath at 80 °C. After stirring at the same temperature for 1 h, the reaction mixture was cooled to room temperature and diluted with ethyl acetate (700 mL). The organic layer was washed with aqueous sat. NaHCO_3_ (3 × 250 mL) and brine (100 mL), dried over MgSO_4_, filtered, and concentrated. The residue was purified by column chromatography (silica gel, hexane/ethyl acetate, 50:3) to give **10** (2.9 g, 40%) as a pale yellow sticky mass; [α]^25^_D_ −7.03 (*c* 0.6, CH_3_OH); UV (CH_3_OH) *λ*_max_ 310.78 nm; ^1^H NMR (CD_3_OD, 500 MHz): *δ* 8.46 (s, 1H), 7.87 (s, 1H), 7.64–7.58 (m, 4H), 7.40–7.33 (m, 4H), 7.31–7.28 (m, 2H), 6.24 (d, *J* = 2.2 Hz, 1H), 5.10 (dd, *J* = 5.5, 2.2 Hz, 1H), 4.96 (dd, *J* = 5.5, 2.1 Hz, 1H), 3.88 (dd, *J* = 10.4, 7.2 Hz, 1H), 3.81 (dd, *J* = 10.4, 7.3 Hz, 1H), 3.75 (td, *J* = 7.2, 2.1 Hz, 1H), 1.55 (s, 3H), 1.28 (s, 3H), 1.04 (S, 9H); ^13^C NMR (CD_3_OD, 100 MHz): *δ* 154.2, 152.5, 152.3, 137.5, 137.4, 136.2, 135.0, 134.7, 131.9, 131.8, 129.7, 129.6, 119.9, 114.0, 90.9, 87.0, 70.1, 67.7, 58.9, 53.5, 28.3, 28.1, 26.1, 20.8; HRMS (ESI-Q-TOF) *m*/*z* [M + H]^+^ for C_30_H_34_ClIN_3_O_3_SSi calculated 706.0818, found 706.0798.

#### 3.2.9. 7-((3aR,4R,6R,6aS)-6-(((*tert*-butyldiphenylsilyl)oxy)methyl)-2,2-dimethyltetrahydrothieno[3,4-d][1,3]dioxol-4-yl)-5-iodo-7H-pyrrolo[2,3-d]pyrimidin-4-amine (**11**)

A solution of **10** (2.9 g, 4.11 mmol) in saturated solution of NH_3_/*t*-BuOH (30 mL) contained in a stainless steel bomb reactor was transferred to a preheated bath at 90 °C and stirred at the same temperature for 24 h. The steel bomb containing reaction mixture was cooled to room temperature and solvent was evaporated under reduced pressure. The residue was purified by silica gel column chromatography (hexane/ethyl acetate, 13:7) to obtain **11** (2.42 g, 86%) as a sticky mass; [α]^25^_D_ −45.49 (*c* 2.4, CH_3_OH); UV (CH_3_OH) *λ*_max_ 286.04 nm; ^1^H NMR (CDCl_3_, 500 MHz): *δ* 8.22 (s, 1H), 7.64–7.62 (m, 4H), 7.43–7.40 (m, 2H), 7.37–7.33 (m, 5H), 6.21 (d, *J* = 2.7 Hz, 1H), 5.88 (br s, 2H), 4.88 (dd, *J* = 5.6, 2.8 Hz, 1H), 4.78 (dd, *J* = 5.6, 2.8 Hz, 1H), 3.86–3.79 (m, 2H), 3.77–3.75 (m, 1H), 1.58 (s, 3H), 1.27 (s, 3H), 1.07 (s, 9H); ^13^C NMR (CDCl_3_, 125 MHz): *δ* 156.7, 151.9, 150.1, 135.5, 135.5, 132.9, 132.8, 129.9, 129.8, 127.8, 127.0, 112.4, 104.5, 89.0, 84.0, 66.3, 65.1, 55.7, 50.5, 27.3, 26.8, 25.1, 19.2; HRMS (ESI-Q-TOF) *m*/*z* [M + H]^+^ for C_30_H_36_IN_4_O_3_SSi calculated 687.1317, found 687.1301. 

General Procedure of Stille Coupling for the Synthesis of **12a** and **12b**. To the compound **11** (1 equiv) in a microwave vial equipped with a septum, catalyst PdCl_2_(PPh_3_)_2_ (15 mol %) was added and degassed THF (2.8 mL/mmol) under nitrogen atmosphere. The resulting solution was degassed for 5 min and a corresponding 2-(tributylstannyl)hetaryl (2.5 equiv) was added. After stirring the reaction mixture in a microwave for 1 h at 70 °C, it was quenched by adding water and brine. The aqueous layer was extracted with ethyl acetate thrice and organics were concentrated. The residue was purified by column chromatography.

#### 3.2.10. 7-((3aR,4R,6R,6aS)-6-(((*tert*-butyldiphenylsilyl)oxy)methyl)-2,2-dimethyltetrahydrothieno[3,4-d][1,3]dioxol-4-yl)-5-(furan-2-yl)-7H-pyrrolo[2,3-d]pyrimidin-4-amine (**12a**)

Compound **12a** (0.16 g, 88%) was obtained from **11** (0.2 g, 0.29 mmol) as a pale yellow sticky mass; silica gel column chromatography (hexane/ethyl acetate, 3:2); [α]^25^_D_ −31.0 (*c* 0.2, CH_3_OH); UV (CH_3_OH) *λ*_max_ 291.76 nm; ^1^H NMR (CDCl_3_, 400 MHz): *δ* 8.27 (s, 1H), 7.64–7.61 (m, 4H), 7.47–7.46 (m, 1H), 7.39–7.36 (m, 3H), 7.33–7.29 (m, 4H), 6.45–6.44 (m, 1H), 6.28–6.27 (m, 2H), 5.98 (br s, 2H), 4.95 (dd, *J* = 5.9, 3.2 Hz, 1H), 4.82 (dd, *J* = 5.9, 3.2 Hz, 1H), 3.92–3.82 (m, 2H), 3.79–3.75 (m, 1H), 1.59 (s, 3H), 1.28 (s, 3H), 1.05 (s, 9H); ^13^C NMR (CDCl_3_, 100 MHz): *δ* 157.2, 152.9, 151.2, 149.0, 141.3, 135.77, 135.74, 133.17, 133.11, 130.1, 130.0, 127.9, 120.0, 112.7, 112.1, 107.3, 105.6, 100.9, 89.2, 84.2, 66.3, 65.3, 55.8, 27.6, 27.0, 25.4, 19.5; HRMS (ESI-Q-TOF) *m*/*z* [M + H]^+^ for C_34_H_39_N_4_O_4_SSi calculated 627.2456, found 627.2463.

#### 3.2.11. 7-((3aR,4R,6R,6aS)-6-(((*tert*-butyldiphenylsilyl)oxy)methyl)-2,2-dimethyltetrahydrothieno[3,4-d][1,3]dioxol-4-yl)-5-(thiophen-2-yl)-7H-pyrrolo[2,3-d]pyrimidin-4-amine (**12b**)

Compound **12b** (0.21 g) was afforded from **11** (0.25 g, 0.36 mmol) in 92% yield as colorless sticky mass; silica gel column chromatography (hexane/ethyl acetate, 3:2); [α]^25^_D_ −51.36 (*c* 0.31, CH_3_OH); UV (CH_3_OH) *λ*_max_ 287.13 nm; ^1^H NMR (CDCl_3_, 400 MHz): *δ* 8.28 (s, 1H), 7.62–7.59 (m, 4H), 7.37–7.34 (m, 3H), 7.32–7.27 (m, 4H), 7.23 (s, 1H), 7.10–7.07 (m, 1H), 7.00–6.99 (m, 1H), 6.28 (d, *J* = 2.7 Hz, 1H), 5.41 (br s, 2H), 4.94 (dd, *J* = 5.5, 3.2 Hz, 1H), 4.81 (dd, *J* = 5.9, 3.2 Hz, 1H), 3.91–3.82 (m, 2H), 3.79–3.74 (m, 1H), 1.59 (s, 3H), 1.28 (s, 3H), 1.03 (s, 9H); ^13^C NMR (CDCl_3_, 100 MHz): *δ* 156.7, 152.1, 150.7, 135.68, 135.63, 129.97, 129.94, 128.0, 127.8, 126.5, 125.8, 121.9, 112.6, 109.4, 102.1, 89.0, 84.1, 66.2, 65.3, 55.6, 27.5, 26.9, 25.3, 19.3; HRMS (ESI-Q-TOF) *m*/*z* [M + H]^+^ for C_34_H_39_N_4_O_3_S_2_Si calculated 643.2227, found 643.2218.

General Procedure for the Synthesis of **1a** and **1b**. To an ice-cooled solution of **12a** and **12b** (1 equiv) in THF (3 mL/mmol) 50% aqueous trifluoroacetic acid (19.5 mL/mmol) was dropwise added and the resulting mixture was stirred at room temperature for 12 h. Acidic solution was basified using a weakly basic anion-exchange resin (Dowex^®^ 66 free base) and stirred for an additional 3 h, filtered, and concentrated under vacuum. The residue was purified by silica gel column chromatography (CH_2_Cl_2_/MeOH, 47:3) to give **1a** and **1b** respectively. 

#### 3.2.12. (2R,3R,4S,5R)-2-(4-Amino-5-(furan-2-yl)-7H-pyrrolo[2,3-d]pyrimidin-7-yl)-5-(hydroxymethyl)tetrahydrothiophene-3,4-diol (**1a**)

It was obtained in 75% yield as white solid; mp 197–199 °C; [α]^25^_D_ −21.14 (*c* 0.05, CH_3_OH); UV (CH_3_OH) *λ*_max_ 290.77 nm; ^1^H NMR (DMSO-*d*_6_, 500 MHz): *δ* 8.13 (s, 1H), 7.95 (s, 1H), 7.78 (s, 1H), 6.90 (br s, 2H, D_2_O exchange), 6.72 (d, *J* = 3.0 Hz, 1H), 6.62–6.61 (m, 1H), 6.17 (d, *J* = 7.0 Hz, 1H), 5.43 (d, *J* = 6.4 Hz, 1H, D_2_O exchange), 5.27 (d, *J* = 4.4 Hz, 1H, D_2_O exchange), 5.18 (t, *J* = 5.5 Hz, 1H, D_2_O exchange), 4.48–4.45 (m, 1H), 4.19–4.18 (m, 1H), 3.78–3.75 (m, 1H), 3.63–3.58 (m, 1H), 3.29–3.28 (m, 1H); ^13^C NMR (DMSO-*d*_6_, 100 MHz): *δ* 157.6, 152.5, 151.6, 149.1, 142.3, 120.7, 112.3, 106.8, 105.6, 99.4, 77.7, 73.6, 63.9, 61.3, 53.4; HRMS (ESI-Q-TOF) *m*/*z* [M + H]^+^ for C_15_H_17_N_4_O_4_S calculated 349.0965, found 349.0974; purity ≥95%.

#### 3.2.13. (2R,3R,4S,5R)-2-(4-Amino-5-(thiophen-2-yl)-7H-pyrrolo[2,3-d]pyrimidin-7-yl)-5-(hydroxymethyl)tetrahydrothiophene-3,4-diol (**1b**)

It was afforded in 81% yield as white solid; mp 166–172 °C; [α]^25^_D_ −23.93 (*c* 0.09, CH_3_OH); UV (CH_3_OH) *λ*_max_ 286.41 nm; ^1^H NMR (CD_3_OD, 500 MHz): *δ* 8.13 (s, 1H), 7.66 (s, 1H), 7.42 (d, *J* = 5.0 Hz, 1H), 7.14–7.11 (m, 2H), 6.22 (d, *J* = 5.9 Hz, 1H), 4.52 (dd, *J* = 5.7, 3.7 Hz, 1H), 4.29 (merged dd, *J*_1_ = *J*_2_ = 3.8 Hz, 1H), 3.88–3.80 (m, 2H), 3.48 (dd, *J* = 9.4, 4.8 Hz, 1H); ^13^C NMR (CD_3_OD, 125 MHz): *δ* 159.5, 153.5, 152.6, 137.5, 129.8, 128.5, 127.6, 124.5, 111.6, 103.5, 80.8, 76.3, 65.1, 64.5, 54.8; HRMS (ESI-Q-TOF) *m*/*z* [M + H]^+^ for C_15_H_17_N_4_O_3_S_2_ calculated 365.0737, found 365.0749; purity ≥95%.

General Procedure of Suzuki Coupling for the Synthesis of **12c**–**f**. A degassed mixture of DMF/H_2_O (6.9 mL/2.7 mL/mmol) was added to a microwave vial containing compound **11** (1 equiv), corresponding boronic ester (1.2 equiv), PdCl_2_(PPh_3_)_2_ (6 mol %), and sodium carbonate (2 equiv). The resulting reaction mixture was heated in a microwave at 70 °C for 1 h. After quenching with water, the reaction mixture was extracted with ethyl acetate thrice. The organic layers were combined, washed with brine, dried over MgSO_4_, filtered, and evaporated. The residue obtained was purified by column chromatography.

#### 3.2.14. 7-((3aR,4R,6R,6aS)-6-(((*tert*-butyldiphenylsilyl)oxy)methyl)-2,2-dimethyltetrahydrothieno[3,4-d][1,3]dioxol-4-yl)-5-vinyl-7H-pyrrolo[2,3-d]pyrimidin-4-amine (**12c**)

Compound **12c** was obtained in 85% yield as yellow sticky mass; silica gel column chromatography (hexane/ethyl acetate, 13:7); [α]^25^_D_ −38.33 (*c* 1.1, CH_3_OH); UV (CH_3_OH) *λ*_max_ 288.95 nm; ^1^H NMR (CD_3_OD, 400 MHz): *δ* 8.03 (s, 1H), 7.64 (t, *J* = 9.2 Hz, 4H), 7.42–7.39 (m, 3H), 7.37–7.31 (m, 4H), 6.91 (dd, *J* = 17.2, 10.8 Hz, 1H), 6.21 (s, 1H), 5.44 (d, *J* = 17.6 Hz, 1H), 5.20 (d, *J* = 10.8 Hz, 1H), 5.07–5.05 (m, 1H), 4.96–4.95 (m, 1H), 3.93–3.89 (m, 1H), 3.86–3.81 (m, 1H), 3.73–3.69 (m, 1H), 1.55 (s, 3H), 1.29 (s, 3H), 1.04 (s, 9H); ^13^C NMR (CD_3_OD, 100 MHz): *δ* 157.8, 151.1, 150.1, 135.37, 135.35, 133.0, 132.7, 129.74, 129.71, 128.1, 127.58, 127.54, 119.4, 115.8. 114.4, 112.0, 101.4, 88.4, 84.5, 66.0, 65.5, 55.7, 26.3, 26.0, 24.0, 18.7; HRMS (ESI-Q-TOF) *m*/*z* [M + H]^+^ for C_32_H_39_N_4_O_3_SSi calculated 587.2507, found 587.2506.

#### 3.2.15. 7-((3aR,4R,6R,6aS)-6-(((*tert*-butyldiphenylsilyl)oxy)methyl)-2,2-dimethyltetrahydrothieno[3,4-d][1,3]dioxol-4-yl)-5-phenyl-7H-pyrrolo[2,3-d]pyrimidin-4-amine (**12d**)

It was obtained in 91% yield as colorless sticky mass; silica gel column chromatography (hexane/ethyl acetate, 1:1); [α]^25^_D_ −46.89 (*c* 0.14, CH_3_OH); UV (CH_3_OH) *λ*_max_ 283.50 nm; ^1^H NMR (CD_3_OD, 400 MHz): *δ* 8.11 (s, 1H), 7.62–7.59 (m, 4H), 7.43–7.41 (m, 3H), 7.39–7.33 (m, 4H), 7.29–7.26 (m, 4H), 7.23 (s, 1H), 6.27 (d, *J* = 2.7 Hz, 1H), 5.09 (dd, *J* = 5.4, 2.7 Hz, 1H), 4.96 (dd, *J* = 5.5, 2.3 Hz, 1H), 3.92–3.82 (m, 2H), 3.75–3.71 (m, 1H), 1.57 (s, 3H), 1.30 (s, 3H), 1.01 (s, 9H); ^13^C NMR (CD_3_OD, 100 MHz): *δ* 157.4, 151.2, 150.1, 135.38, 135.34, 134.1, 132.9, 132.7, 129.7, 128.7, 128.6, 127.55, 127.50, 127.1, 120.7, 117.9, 111.9, 101.3, 88.6, 84.7, 66.5, 65.6, 56.0, 26.2, 25.9, 24.0, 18.6; HRMS (ESI-Q-TOF) *m*/*z* [M + H]^+^ for C_36_H_41_N_4_O_3_SSi calculated 637.2663, found 637.2646. 

#### 3.2.16. 4-(4-(4-Amino-7-((3aR,4R,6R,6aS)-6-(((*tert*-butyldiphenylsilyl)oxy)methyl)-2,2-dimethyltetrahydrothieno[3,4-d][1,3]dioxol-4-yl)-7H-pyrrolo[2,3-d]pyrimidin-5-yl)phenyl)thiomorpholine 1,1-dioxide (**12e**)

It was afforded in 89% yield as colorless sticky mass; silica gel column chromatography (hexane/ethyl acetate, 11:9); [α]^25^_D_ −28.78 (*c* 0.38, CH_3_OH); UV (CH_3_OH) *λ*_max_ 270.04 nm; ^1^H NMR (CDCl_3_, 400 MHz): *δ* 8.27 (s, 1H), 7.61–7.59 (m, 4H), 7.40–7.34 (m, 4H), 7.32–7.27 (m, 4H), 7.14 (s, 1H), 6.94–6.92 (m, 2H), 6.31 (d, *J* = 3.6 Hz, 1H), 5.28 (br s, 2H), 4.99 (dd, *J* = 5.9, 3.2 Hz, 1H), 4.82 (dd, *J* = 5.9, 2.7 Hz, 1H), 3.93–3.86 (m, 4H), 3.84–3.82 (m, 2H), 3.79–3.75 (m, 1H), 3.13–3.11 (m, 4H), 1.60 (s, 3H), 1.29 (s, 3H), 1.03 (s, 9H); ^13^C NMR (CDCl_3_, 100 MHz): *δ* 156.6, 151.5, 150.7, 146.9, 135.66, 135.62, 133.0, 130.3, 129.96, 129.92, 127.8, 126.5, 120.6, 116.8, 116.6, 112.7, 101.9, 88.9, 84.0, 66.0, 65.2, 55.5, 50.5, 47.6, 27.5, 26.9, 25.3, 19.3; HRMS (ESI-Q-TOF) *m*/*z* [M + H]^+^ for C_40_H_48_N_5_O_5_S_2_Si calculated 770.2861, found 770.2865.

#### 3.2.17. N-(4-(4-Amino-7-((3aR,4R,6R,6aS)-6-(((*tert*-butyldiphenylsilyl)oxy)methyl)-2,2-dimethyltetrahydrothieno[3,4-d][1,3]dioxol-4-yl)-7H-pyrrolo[2,3-d]pyrimidin-5-yl)phenyl)ethanesulfonamide (**12f**)

Compound **12f** was obtained in 87% yield as colorless sticky mass; silica gel column chromatography (hexane/ethyl acetate, 2:3); [α]^25^_D_ −48.16 (*c* 0.09, CH_3_OH); UV (CH_3_OH) *λ*_max_ 286.04 nm; ^1^H NMR (CDCl_3_, 500 MHz): *δ* 8.29 (s, 1H), 7.60–7.59 (m, 4H), 7.39–7.34 (m, 2H), 7.32–7.30 (m, 4H), 7.28–7.27 (m, 2H), 7.25–7.24 (m, 2H), 7.20 (s, 1H), 6.66 (br s, 1H), 6.30 (d, *J* = 2.9 Hz, 1H), 5.37 (br s, 2H), 4.97 (dd, *J* = 5.6, 3.0 Hz, 1H), 4.82 (dd, *J* = 5.6, 2.7 Hz, 1H), 3.90–3.82 (m, 2H), 3.80–3.78 (m, 1H), 3.15 (q, *J* = 7.3 Hz, 2H), 1.60 (s, 3H), 1.39 (t, *J* = 7.3 Hz, 3H), 1.29 (s, 3H), 1.02 (s, 9H); ^13^C NMR (CDCl_3_, 100 MHz): *δ* 156.3, 151.1, 150.7, 136.4, 135.63, 135.60, 132.8, 130.9, 130.0, 127.8, 121.3, 120.9, 116.4, 112.6, 101.7, 89.1, 84.1, 66.4, 65.2, 55.6, 46.3, 27.5, 26.9, 25.3, 19.3, 8.4; HRMS (ESI-Q-TOF) *m*/*z* [M + H]^+^ for C_38_H_46_N_5_O_5_S_2_Si calculated 744.2704, found 744.2698.

#### 3.2.18. (2R,3R,4S,5R)-2-(4-Amino-5-vinyl-7H-pyrrolo[2,3-d]pyrimidin-7-yl)-5-(hydroxymethyl)tetrahydrothiophene-3,4-diol (**1c**)

Compound **12c** (0.12 g, 0.20 mmol) was converted to **1c** as described for **1a**, affording white solid (47.5 mg, 78%); silica gel column chromatography (CH_2_Cl_2_/MeOH, 19:1); mp 110–112 °C; [α]^25^_D_ −41.86 (*c* 0.20, CH_3_OH); UV (CH_3_OH) *λ*_max_ 288.50 nm; ^1^H NMR (DMSO-*d*_6_, 500 MHz): *δ* 8.04 (s, 1H), 7.77 (s, 1H), 7.10 (dd, *J* = 17.2, 10.9 Hz, 1H), 6.70 (br s, 2H, D_2_O exchange), 6.12 (d, *J* = 7.0 Hz, 1H), 5.59 (d, *J* = 17.1 Hz, 1H), 5.38 (d, *J* = 6.4 Hz, 1H, D_2_O exchange), 5.25 (d, *J* = 4.4 Hz, 1H, D_2_O exchange), 5.17 (t, *J* = 5.5 Hz, 1H, D_2_O exchange), 5.11 (d, *J* = 11.0 Hz, 1H), 4.46–4.41 (m, 1H), 4.18–4.14 (m, 1H), 3.77–3.72 (m, 1H), 3.61–3.55 (m, 1H), 3.27–3.24 (m, 1H); ^13^C NMR (CD_3_OD, 100 MHz): *δ* 157.8, 150.9, 150.6, 128.3, 119.9, 115.3, 113.9, 78.6, 74.1, 63.0, 62.2, 52.6; HRMS (ESI-Q-TOF) *m*/*z* [M + H]^+^ for C_13_H_17_N_4_O_3_S calculated 309.1016, found 309.1018; purity ≥95%.

#### 3.2.19. (2R,3R,4S,5R)-2-(4-Amino-5-phenyl-7H-pyrrolo[2,3-d]pyrimidin-7-yl)-5-(hydroxymethyl)tetrahydrothiophene-3,4-diol (**1d**)

Compound **1d** was prepared from **12d** (0.16 g, 0.25 mmol) as described for **1a**, affording white solid (72.5 mg, 81%); silica gel column chromatography (CH_2_Cl_2_/MeOH, 19:1); mp 102–104 °C; [α]^25^_D_ −46.30 (*c* 0.06, CH_3_OH); UV (CH_3_OH) *λ*_max_ 280.95 nm; ^1^H NMR (DMSO-*d*_6_, 500 MHz): *δ* 8.14 (s, 1H), 7.66 (s, 1H), 7.50–7.46 (m, 4H), 7.38–7.34 (m, 1H), 6.18 (d, *J* = 6.9 Hz, 1H), 5.42 (d, *J* = 6.4 Hz, 1H), 5.28 (d, *J* = 4.5 Hz, 1H), 5.16 (t, *J* = 5.5 Hz, 1H), 4.51–4.47 (m, 1H), 4.19–4.16 (m, 1H), 3.77–3.71 (m, 1H), 3.61–3.56 (m, 1H), 3.29–3.25 (m, 1H); ^13^C NMR (CD_3_OD, 125 MHz): *δ* 159.5, 153.1, 152.8, 136.6, 130.9, 130.7, 129.2, 123.4, 119.7, 103.2, 80.9, 76.4, 65.2, 64.5, 54.8; HRMS (ESI-Q-TOF) *m*/*z* [M + H]^+^ for C_17_H_19_N_4_O_3_S calculated 359.1172, found 359.1177; purity ≥95%. 

#### 3.2.20. 4-(4-(4-Amino-7-(3,4-dihydroxy-5-(hydroxymethyl)tetrahydrothiophen-2-yl)-7H-pyrrolo[2,3-d]pyrimidin-5-yl)phenyl)thiomorpholine 1,1-dioxide (**1e**)

It was obtained from **12e** (0.1 g, 0.12 mmol) as described for **1a**, as white solid (47.1 mg, 80%); silica gel column chromatography (CH_2_Cl_2_/MeOH, 93:7); mp 188–192 °C; [α]^25^_D_ −24.29 (*c* 0.05, CH_3_OH); UV (CH_3_OH) *λ*_max_ 270.04 nm; ^1^H NMR (DMSO-*d*_6_, 500 MHz): *δ* 8.13 (s, 1H), 7.54 (s, 1H), 7.35 (merged dd, *J*_1_ = *J*_2_ = 8.4 Hz, 2H), 7.13 (merged dd, *J*_1_ = *J*_2_ = 8.5 Hz, 2H), 6.18 (d, *J* = 6.9 Hz, 1H), 5.40 (d, *J* = 6.4 Hz, 1H), 5.26 (d, *J* = 4.3 Hz, 1H), 5.16 (t, *J* = 5.4 Hz, 1H), 4.49–4.47 (m, 1H), 4.18–4.17 (m, 1H), 3.87–3.82 (m, 4H), 3.75–3.72 (m, 1H), 3.60–3.57 (m, 1H), 3.29–3.27 (m, 1H), 3.17–3.12 (m, 4H); ^13^C NMR (DMSO-*d*_6_, 125 MHz): *δ* 157.2, 151.6, 151.0, 146.2, 129.4, 125.1, 120.1, 116.2, 116.0, 100.3, 77.3, 73.3, 63.3, 60.9, 52.8, 49.7, 46.5; HRMS (ESI-Q-TOF) *m*/*z* [M + H]^+^ for C_21_H_26_N_5_O_5_S_2_ calculated 492.137, found 492.1388; purity ≥95%.

#### 3.2.21. N-(4-(4-Amino-7-(3,4-dihydroxy-5-(hydroxymethyl)tetrahydrothiophen-2-yl)-7H-pyrrolo[2,3-d]pyrimidin-5-yl)phenyl)ethanesulfonamide (**1f**)

It was afforded from **12f** (90 mg, 0.12 mmol) as described for **1a**, as white solid (44.6 mg, 80%); silica gel column chromatography (CH_2_Cl_2_/MeOH, 91:9); mp 127–132 °C; [α]^25^_D_ −35.60 (*c* 0.05, CH_3_OH); UV (CH_3_OH) *λ*_max_ 283.86 nm; ^1^H NMR (DMSO-*d*_6_, 500 MHz): *δ* 9.87 (s, 1H), 8.14 (s, 1H), 7.60 (s, 1H), 7.42 (merged dd, *J*_1_ = *J*_2_ = 8.2 Hz, 2H), 7.31 (merged dd, *J*_1_ = *J*_2_ = 8.1 Hz, 2H), 6.18 (d, *J* = 6.9 Hz, 1H), 5.40 (d, *J* = 6.4 Hz, 1H), 5.26 (d, *J* = 4.3 Hz, 1H), 5.15 (t, *J* = 5.4 Hz, 1H), 4.49–4.46 (m, 1H), 4.19–4.17 (m, 1H), 3.76–3.72 (m, 1H), 3.61–3.57 (m, 1H), 3.29–3.27 (m, 1H), 3.13 (q, *J* = 7.2 Hz, 2H), 1.22 (t, *J* = 7.2 Hz, 3H); ^13^C NMR (DMSO-*d*_6_, 125 MHz): *δ* 157.2, 151.6, 151.1, 137.1, 129.7, 129.2, 120.7, 119.8, 115.9, 100.1, 77.3, 73.3, 63.3, 60.9, 52.8, 45.0, 8.02; HRMS (ESI-Q-TOF) *m*/*z* [M + H]^+^ for C_19_H_24_N_5_O_5_S_2_ calculated 466.1213, found 466.1225; purity ≥95%.

General Procedure of Sonogashira Coupling for the Synthesis of **13**. To a microwave vial equipped with a septum, containing **11** (1 equiv), CuI (25 mol %), and PdCl_2_(PPh_3_)_2_ (10 mol %) a degassed mixture of DMF-Et_3_N (6.1 mL/mmol, 4:1) was added. The resulting reaction mixture was degassed with nitrogen for 5 min before adding corresponding alkyne (1.1 equiv) and heated in a microwave at 50 °C for 1 h. The reaction mixture was partitioned between ethyl acetate and water. The combined organic layer was dried over MgSO_4_, filtered, and evaporated. The resulting residue was purified by silica gel chromatography to afford the respective compounds.

#### 3.2.22. 7-((3aR,4R,6R,6aS)-6-(((*tert*-butyldiphenylsilyl)oxy)methyl)-2,2-dimethyltetrahydrothieno[3,4-d][1,3]dioxol-4-yl)-5-((trimethylsilyl)ethynyl)-7H-pyrrolo[2,3-d]pyrimidin-4-amine (**13**)

The desired compound **13** was obtained from **11** (1.67 g, 2.43 mmol) in 93% yield as sticky mass; silica gel column chromatography (hexane/ethyl acetate, 4:1); [α]^25^_D_ −74.11 (*c* 0.65, CH_3_OH); UV (CH_3_OH) *λ*_max_ 284.59 nm; ^1^H NMR (CDCl_3_, 500 MHz): *δ* 8.24 (s, 1H), 7.65–7.62 (m, 4H), 7.42–7.39 (m, 3H), 7.37–7.34 (m, 4H), 6.19 (d, *J* = 2.6 Hz, 1H), 5.73 (br s, 2H), 4.89 (dd, *J* = 5.6, 2.6 Hz, 1H), 4.78 (dd, *J* = 5.6, 2.3 Hz, 1H), 3.87–3.84 (m, 1H), 3.79–3.73 (m, 2H), 1.57 (s, 3H), 1.27 (s, 3H), 1.07 (s, 9H), 0.24 (s, 9H); ^13^C NMR (CDCl_3_, 100 MHz): *δ* 157.3, 153.0, 149.7, 135.7, 135.6, 133.0, 132.9, 130.0, 127.9, 126.9, 112.5, 104.0, 98.4, 97.7, 96.2, 89.3, 84.2, 66.5, 65.2, 56.0, 27.4, 26.9, 25.2, 19.3, 0.01; HRMS (ESI-Q-TOF) *m*/*z* [M + H]^+^ for C_35_H_45_N_4_O_3_SSi_2_ calculated 657.2745, found 657.2739.

#### 3.2.23. ((3aS,4R,6R,6aR)-6-(4-Amino-5-ethynyl-7H-pyrrolo[2,3-d]pyrimidin-7-yl)-2,2-dimethyltetrahydrothieno[3,4-d][1,3]dioxol-4-yl)methanol (**14**)

To the stirred solution of **13** (0.58 g, 0.88 mmol) in anhydrous THF (5.8 mL) a solution of 1 M TBAF in THF (2.64 mL, 2.64 mmol) was added under nitrogen atmosphere and the resulting reaction mixture was stirred at room temperature for 40 min. The reaction was quenched with saturated aqueous NH_4_Cl solution (5 mL) and extracted with ethyl acetate (3 × 150 mL). The combined organic layer was washed with brine (100 mL), dried over MgSO_4_, filtered, and concentrated to give the residue. Upon silica gel column chromatography (methylene chloride/methanol, 97:3), compound **14** (0.27 g, 90%) was obtained as pale yellow syrup; [α]^25^_D_ −51.62 (*c* 1.15, CH_3_OH); UV (CH_3_OH) *λ*_max_ 280.95 nm; ^1^H NMR (CD_3_OD, 500 MHz): *δ* 8.12 (s, 1H), 7.78 (s, 1H), 6.27 (d, *J* = 3.0 Hz, 1H), 5.14 (dd, *J* = 5.3, 3.1 Hz, 1H), 4.97 (dd, *J* = 5.4, 2.2 Hz, 1H), 3.79–3.74 (m, 2H), 3.73 (s, 1H), 3.71–3.68 (m, 1H), 1.58 (s, 3H), 1.32 (s, 3H); ^13^C NMR (CD_3_OD, 125 MHz): *δ* 159.9, 154.4, 151.2, 129.7, 114.1, 105.2, 97.7, 91.1, 87.4, 77.9, 68.7, 65.8, 57.7, 28.6, 26.2; HRMS (ESI-Q-TOF) *m*/*z* [M + H]^+^ for C_16_H_19_N_4_O_3_S calculated 347.1172, found 347.1168.

#### 3.2.24. (2R,3R,4S,5R)-2-(4-Amino-5-ethynyl-7H-pyrrolo[2,3-d]pyrimidin-7-yl)-5-(hydroxymethyl)tetrahydrothiophene-3,4-diol (**1g**)

To a solution of **14** (0.21 g, 0.60 mmol) in THF (6 mL) 2 N HCl solution (6 mL) was added dropwise at 0 °C and the reaction mixture was stirred at room temperature for 15 h. A weakly basic anion-exchange resin (Dowex^®^ 66 free base) was added to the resulting solution to neutralize HCl and stirred for additional 3 h. The solution was filtered, evaporated, and the residue was purified by silica gel column chromatography (methylene chloride/methanol, 47:3) to afford **1g** (0.13 g, 72%) as white solid; mp 235–237 °C; [α]^25^_D_ −47.30 (*c* 0.05, CH_3_OH); UV (CH_3_OH) *λ*_max_ 280.59 nm; ^1^H NMR (DMSO-*d*_6_, 500 MHz): *δ* 8.12 (s, 1H), 7.93 (s, 1H), 6.06 (d, *J* = 6.9 Hz, 1H), 5.43 (d, *J* = 6.3 Hz, 1H), 5.27 (d, *J* = 4.3 Hz, 1H), 5.17 (t, *J* = 5.3 Hz, 1H), 4.45–4.42 (m, 1H), 4.28 (s, 1H), 4.16–4.14 (m, 1H), 3.76–3.71 (m, 1H), 3.61–3.57 (m, 1H), 3.28–3.25 (m, 1H); ^13^C NMR (DMSO-*d*_6_, 150 MHz): *δ* 157.3, 152.6, 149.8, 127.6, 102.2, 94.0, 83.0, 77.34, 77.30, 73.2, 63.1, 61.1, 53.0; HRMS (ESI-Q-TOF) *m*/*z* [M + H]^+^ for C_13_H_15_N_4_O_3_S calculated 307.0859, found 307.0863; purity ≥95%. 

#### 3.2.25. 7-((3aR,4R,6R,6aS)-6-(((*tert*-butyldiphenylsilyl)oxy)methyl)-2,2-dimethyltetrahydrothieno[3,4-d][1,3]dioxol-4-yl)-5-(prop-1-yn-1-yl)-7H-pyrrolo[2,3-d]pyrimidin-4-amine (**15a**)

Following the procedure described to synthesize **13**, compound **11** (200 mg, 0.29 mmol) afforded **15a** (164 mg, 96%) as sticky mass; silica gel column chromatography (hexane/ethyl acetate, 3:1); [α]^25^_D_ −63.53 (*c* 0.195, CH_3_OH); UV (CH_3_OH) *λ*_max_ 282.77 nm; ^1^H NMR (CDCl_3_, 400 MHz): *δ* 8.22 (s, 1H), 7.66–7.63 (m, 4H), 7.41–7.40 (m, 2H), 7.38–7.34 (m, 4H), 7.30 (s, 1H), 6.20 (d, *J* = 2.8 Hz, 1H), 5.74 (br s, 2H), 4.90 (dd, *J* = 5.6, 2.8 Hz, 1H), 4.80 (dd, *J* = 6.0, 5.8 Hz, 1H), 3.88 (dd, *J* = 10, 6.8 Hz, 1H), 3.82–3.75 (m, 2H), 2.07 (s, 3H), 1.58 (s, 3H), 1.28 (s, 3H), 1.08 (s, 9H); ^13^C NMR (CDCl_3_, 100 MHz): *δ* 157.0, 152.3, 149.4, 135.7, 135.6, 133.0, 132.9, 129.9, 127.8, 125.8, 112.6, 103.8, 97.0, 89.1, 88.4, 84.1, 72.5, 66.3, 65.2, 55.9, 27.4, 26.9, 25.2, 19.3, 4.6; HRMS (ESI-Q-TOF) *m*/*z* [M + H]^+^ for C_33_H_39_N_4_O_3_SSi calculated 599.2507, found 599.2512.

#### 3.2.26. 5-(But-1-yn-1-yl)-7-((3aR,4R,6R,6aS)-6-(((*tert*-butyldiphenylsilyl)oxy)methyl)-2,2-dimethyltetrahydrothieno[3,4-d][1,3]dioxol-4-yl)-7H-pyrrolo[2,3-d]pyrimidin-4-amine (**15b**)

It was obtained from **11** (234 mg, 0.34 mmol) as described for **13**, as sticky mass (189 mg, 91%); silica gel column chromatography (hexane/ethyl acetate, 7:3); [α]^25^_D_ −71.76 (*c* 0.06, CH_3_OH); UV (CH_3_OH) *λ*_max_ 283.50 nm; ^1^H NMR (CDCl_3_, 500 MHz): *δ* 8.22 (s, 1H), 7.65–7.62 (m, 4H), 7.42–7.39 (m, 2H), 7.37–7.34 (m, 4H), 7.30 (s, 1H), 6.19 (d, *J* = 2.7 Hz, 1H), 5.68 (br s, 2H), 4.89 (dd, *J* = 5.7, 2.8 Hz, 1H), 4.79 (dd, *J* = 5.6, 2.8 Hz, 1H), 3.87–3.85 (m, 1H), 3.80–3.77 (m, 1H), 3.76–3.74 (m, 1H), 2.43 (q, *J* = 14.9, 7.5 Hz, 2H), 1.57 (s, 3H), 1.27 (s, 3H), 1.23 (t, *J* = 7.5 Hz, 3H), 1.06 (s, 9H); ^13^C NMR (CDCl_3_, 100 MHz): *δ* 157.0, 152.4, 149.4, 135.6, 135.5, 132.99, 132.9, 129.8, 127.8, 127.7, 125.6, 112.5, 103.8, 96.8, 94.1, 89.0, 84.0, 72.7, 66.1, 65.1, 55.8, 27.3, 26.8, 25.1, 19.2, 13.9, 13.2; HRMS (ESI-Q-TOF) *m*/*z* [M + H]^+^ for C_34_H_41_N_4_O_3_SSi calculated 613.2663, found 613.2669.

#### 3.2.27. 7-((3aR,4R,6R,6aS)-6-(((*tert*-butyldiphenylsilyl)oxy)methyl)-2,2-dimethyltetrahydrothieno[3,4-d][1,3]dioxol-4-yl)-5-(3,3-dimethylbut-1-yn-1-yl)-7H-pyrrolo[2,3-d]pyrimidin-4-amine (**15c**)

For the synthesis of **15c**, compound **11** (300 mg, 0.43 mmol) was treated as described for **13**, yielding sticky mass (224 mg, 80%); silica gel column chromatography, hexane/ethyl acetate, 13:7; [α]^25^_D_ −67.09 (*c* 0.265, CH_3_OH); UV (CH_3_OH) *λ*_max_ 283.86 nm; ^1^H NMR (CDCl_3_, 500 MHz): *δ* 8.21 (s, 1H), 7.64 (t, *J* = 6.4 Hz, 4H), 7.42–7.40 (m, 2H), 7.38–7.34 (m, 4H), 7.32 (s, 1H), 6.20 (d, *J* = 2.6 Hz, 1H), 5.83 (br s, 2H), 4.88 (dd, *J* = 5.6, 2.7 Hz, 1H), 4.77 (dd, *J* = 5.5, 2.5 Hz, 1H), 3.87 (dd, *J* = 9.7, 6.2 Hz, 1H), 3.79–3.73 (m, 2H), 1.57 (s, 3H), 1.31 (s, 9H), 1.26 (s, 3H), 1.07 (s, 9H); ^13^C NMR (CDCl_3_, 100 MHz): *δ* 157.2, 152.6, 149.6, 135.7, 135.6, 133.0, 132.9, 129.9, 127.9, 125.3, 112.5, 103.9, 101.1, 96.9, 89.2, 84.1, 72.1, 66.2, 65.2, 55.9, 31.0, 28.3, 27.4, 26.9, 25.2, 19.3; HRMS (ESI-Q-TOF) *m*/*z* [M + H]^+^ for C_36_H_45_N_4_O_3_SSi calculated 641.2976, found 641.2991.

#### 3.2.28. 7-((3aR,4R,6R,6aS)-6-(((*tert*-butyldiphenylsilyl)oxy)methyl)-2,2-dimethyltetrahydrothieno[3,4-d][1,3]dioxol-4-yl)-5-(cyclopropylethynyl)-7H-pyrrolo[2,3-d]pyrimidin-4-amine (**15d**)

It was obtained from **11** (214 mg, 0.31 mmol) as described for **13**, as sticky mass (180 mg, 92%); silica gel column chromatography, hexane/ethyl acetate, 13:7; [α]^25^_D_ −70.38 (*c* 0.10, CH_3_OH); UV (CH_3_OH) *λ*_max_ 284.50 nm; ^1^H NMR (CDCl_3_, 400 MHz): *δ* 8.21 (s, 1H), 7.66–7.63 (m, 4H), 7.44–7.40 (m, 2H), 7.39–7.34 (m, 4H), 7.31 (s, 1H), 6.19 (d, *J* = 3.2 Hz, 1H), 5.93 (br s, 2H), 4.88 (dd, *J* = 5.9, 3.2 Hz, 1H), 4.79 (dd, *J* = 5.9, 2.7 Hz, 1H), 3.89–3.85 (m, 1H), 3.82–3.73 (m, 2H), 1.58 (s, 3H), 1.49–1.44 (m, 1H), 1.27 (s, 3H), 1.07 (s, 9H), 0.92–0.85 (m, 2H), 0.79–0.75 (m, 2H); ^13^C NMR (CDCl_3_, 100 MHz): *δ* 156.4, 149.0, 135.6, 135.5, 132.9, 132.8, 129.9, 127.8, 126.2, 112.5, 103.8, 97.1, 89.1, 84.1, 68.0, 66.3, 65.1, 55.8, 27.3, 26.8, 25.1, 19.2, 8.7, 0.2; HRMS (ESI-Q-TOF) *m*/*z* [M + H]^+^ for C_35_H_41_N_4_O_3_SSi calculated 625.2663, found 625.2667.

#### 3.2.29. (2R,3R,4S,5R)-2-(4-Amino-5-(prop-1-yn-1-yl)-7H-pyrrolo[2,3-d]pyrimidin-7-yl)-5-(hydroxymethyl)tetrahydrothiophene-3,4-diol (**1h**)

Compound **15a** (150 mg, 0.25 mmol) was converted to **1h** (66.4 mg, 83%) yielding white solid, using the procedure described for **1a**; silica gel column chromatography (CH_2_Cl_2_/MeOH, 47:3); mp 190–192 °C; [α]^25^_D_ −51.14 (*c* 0.05, CH_3_OH); UV (CH_3_OH) *λ*_max_ 282.77 nm; ^1^H NMR (DMSO-*d*_6_, 500 MHz): *δ* 8.10 (s, 1H), 7.75 (s, 1H), 6.05 (d, *J* = 6.7 Hz, 1H), 5.40 (d, *J* = 6.2 Hz, 1H), 5.25 (d, *J* = 4.1 Hz, 1H), 5.16 (t, *J* = 5.3 Hz, 1H), 4.42–4.39 (m, 1H), 4.16–4.13 (m, 1H), 3.75–3.70 (m, 1H), 3.61–3.56 (m, 1H), 3.27–3.24 (m, 1H), 2.08 (s, 3H); ^13^C NMR (DMSO-*d*_6_, 125 MHz): *δ* 157.4, 152.5, 149.7, 125.8, 102.1, 95.7, 88.4, 77.3, 73.2, 72.6, 63.1, 61.1, 52.9, 4.2; HRMS (ESI-Q-TOF) *m*/*z* [M + H]^+^ for C_14_H_17_N_4_O_3_S calculated 321.1016, found 321.1013; purity ≥95%.

#### 3.2.30. (2R,3R,4S,5R)-2-(4-Amino-5-(but-1-yn-1-yl)-7H-pyrrolo[2,3-d]pyrimidin-7-yl)-5-(hydroxymethyl)tetrahydrothiophene-3,4-diol (**1i**)

Following the procedure described for **1a**; compound **15b** (176 mg, 0.28 mmol) yielded **1i** (80.4 mg, 86%) as white solid; silica gel column chromatography (CH_2_Cl_2_/MeOH, 47:3); mp 161–162 °C; [α]^25^_D_ −32.48 (*c* 0.05, CH_3_OH); UV (CH_3_OH) *λ*_max_ 283.86 nm; ^1^H NMR (DMSO-*d*_6_, 500 MHz): *δ* 8.10 (s, 1H), 7.76 (s, 1H), 6.06 (d, *J* = 6.8 Hz, 1H), 5.40 (*J* = 6.3 Hz, 1H), 5.26 (*J* = 4.4 Hz, 1H), 5.16 (t, *J* = 5.4 Hz, 1H), 4.43–4.40 (m, 1H), 4.16–4.13 (m, 1H), 3.75–3.70 (m, 1H), 3.60–3.56 (m, 1H), 3.27–3.24 (m, 1H), 2.47 (q, *J* = 14.9, 7.4 Hz, 2H), 1.17 (t, *J* = 7.4 Hz, 3H); ^13^C NMR (CD_3_OD, 100 MHz): *δ* 157.6, 151.6, 149.2, 126.0, 103.1, 97.0, 93.8, 78.8, 74.2, 72.0, 63.0, 62.5, 52.7, 12.9, 12.5; HRMS (ESI-Q-TOF) *m*/*z* [M + H]^+^ for C_15_H_19_N_4_O_3_S calculated 335.1172, found 335.1157; purity ≥95%.

#### 3.2.31. (2R,3R,4S,5R)-2-(4-Amino-5-(3,3-dimethylbut-1-yn-1-yl)-7H-pyrrolo[2, 3-d]pyrimidin-7-yl)-5-(hydroxymethyl)tetrahydrothiophene-3,4-diol (**1j**)

Compound **15c** (200 mg, 0.31 mmol) was converted to **1j** (91.8 mg, 82%) as white solid, by following the procedure described for **1a**; silica gel column chromatography (CH_2_Cl_2_/MeOH, 47:3); mp 144–146 °C; [α]^25^_D_ −61.72 (*c* 0.06, CH_3_OH); UV (CH_3_OH) *λ*_max_ 287.50 nm; ^1^H NMR (DMSO-*d*_6_, 500 MHz): *δ* 8.11 (s, 1H), 7.76 (s, 1H), 6.06 (d, *J* = 7.0 Hz, 1H), 5.39 (d, *J* = 6.4 Hz, 1H), 5.27 (d, *J* = 4.4 Hz, 1H), 5.16 (t, *J* = 5.5 Hz, 1H), 4.45–4.41 (m, 1H), 4.16–4.13 (m, 1H), 3.76–3.71 (m, 1H), 3.61–3.55 (m, 1H), 3.27–3.24 (m, 1H), 1.31 (s, 9H); ^13^C NMR (DMSO-*d*_6_, 100 MHz): *δ* 157.5, 152.6, 149.8, 125.7, 102.2, 100.3, 95.3, 77.3, 73.2, 72.5, 63.2, 60.9, 52.9, 30.6, 27.8; HRMS (ESI-Q-TOF) *m*/*z* [M + H]^+^ for C_17_H_23_N_4_O_3_S calculated 363.1485, found 363.1492; purity ≥95%.

#### 3.2.32. (2R,3R,4S,5R)-2-(4-Amino-5-(cyclopropylethynyl)-7H-pyrrolo[2,3-d]pyrimidin-7-yl)-5-(hydroxymethyl)tetrahydrothiophene-3,4-diol (**1k**)

Compound **15d** (156 mg, 0.24 mmol) was converted to **1k** (71.4 mg, 86%) as white solid, by following the procedure described for **1a**; silica gel column chromatography (CH_2_Cl_2_/MeOH, 47:3); mp 166–168 °C; [α]^25^_D_ −50.34 (*c* 0.05, CH_3_OH); UV (CH_3_OH) *λ*_max_ 284.22 nm; ^1^H NMR (CD_3_OD, 400 MHz): *δ* 8.09 (s, 1H), 7.67 (s, 1H), 6.11 (d, *J* = 5.6 Hz, 1H), 4.44 (dd, *J* = 5.6, 3.6 Hz, 1H), 4.24 (merged dd, *J*_1_ = *J*_2_ = 4.0 Hz, 1H), 3.89–3.79 (m, 2H), 3.49–3.44 (m, 1H), 1.57–1.50 (m, 1H), 0.94–0.88 (m, 2H), 0.78–0.74 (m, 2H); ^13^C NMR (CD_3_OD, 100 MHz): *δ* 159.2, 153.2, 150.7, 127.8, 104.7, 98.3, 97.1, 80.2, 75.6, 69.1, 64.5, 64.0, 54.2, 9.1, 0.9; HRMS (ESI-Q-TOF) *m*/*z* [M + H]^+^ for C_16_H_19_N_4_O_3_S calculated 347.1172, found 347.1159; purity ≥95%.

### 3.3. Cell Proliferation Inhibition Assay (SRB Assay)

Human lung cancer cells (A549), colorectal cancer (HCT116) cells, breast cancer cells (MDA-MB-231), liver cancer cells (SK-HEP-1), and prostate cancer cells (PC-3) were purchased from the American Type Culture Collection (Manassas, VA, USA). Human gastric cancer cells (SNU-638) were purchased from the Korean Cell Line Bank (Seoul, Korea). Cells were cultured in medium (Dulbecco’s modified Eagle’s medium for MDA-MB-231 and SK-HEP-1 cells; Roswell Park Memorial Institute 1640 for A549, HCT116, SNU-638, PC-3 cells) supplemented with penicillin-streptomycin and 10% fetal bovine serum at 37 °C in a humidified incubator with 5% carbon dioxide. Cells were seeded at a density of 4–7 × 10^4^ cells/mL in 96-well culture plates, and then treated with indicated compounds for 72 h. At the end of the experiment, cells were fixed with 10% trichloroacetic acid (TCA) solution and subjected to sulforhodamine B (SRB) assay to determine cell proliferation [42]. The percentage of cell proliferation was calculated with the following formula:

Cell proliferation (%) = 100 × [(A treated − A zero day)/(A control − A zero day)],

where A is the average absorbance. The IC_50_ values were calculated through non-linear regression analysis using TableCurve 2D v5.01 (Systat Software Inc., San Jose, CA, USA). All experiments were performed in triplicate and data shown are representative of two or three independent experiments.

#### Cell Culture

The human colon cancer (KM12) and renal cancer (ACHN) cell lines were obtained from the Korean Cell Line Bank (Seoul, Korea). Cells were grown in DMEM supplemented with 10% fetal bovine serum (FBS) and antibiotics-antimycotics (PSF: 100 units/mL penicillin G sodium, 100 µg/mL streptomycin and 250 ng/mL amphotericin B). All cells were incubated at 37 °C in a humidified atmosphere containing 5% CO_2_ and subcultured twice a week.

### 3.4. Kinome Scan Assays

The kinome scan assays were carried out at Eurofins DiscoverX Corporation. For kinome scan profiling of compound **1g**, it was screened at 1μM against 96 kinases (*N* = 2 independent experiments) [26]. The results for binding interactions are reported as % inhibition, where higher values indicate strong affinity; see the Supporting Information, Table S1 for full kinome profile. For kinase inhibition profile of compounds **1a**–**k** [see the Supporting information, Table S2, whose IC_50_ values were determined using an 11-point 3-fold serial dilution of each test compound using their KINOMEscan assay and *K*_i_ was determined by the Cheng-Prusoff equation.^.^

### 3.5. Metabolic Stability 

Phosphate buffer (0.1 M, pH 7.4) containing human liver microsomes (0.5 mg/mL) and test compound (a final concentration of 1 μM) were pre-incubated for 5 min at 37 °C. NADPH regeneration system solution was added to it and incubated for 30 min at 37 °C. Acetonitrile solution containing chlorpropamide was added at the end of the reaction. The sample was centrifuged for 5 min (14,000× *g* rpm, 4 °C) and the supernatant was injected into the LC-MS/MS system for the analysis. The amount of substrate that remained after the reaction was analyzed using the Shimadzu Nexera XR system and TSQ vantage (Thermo). Kinetex C18 column (2.1 × 100 mm, 2.6 μm particle size; Phenomenex) was used for HPLC. The mobile phase used contained 0.1% formic acid in distilled water (A) and 0.1% formic acid containing acetonitrile (B). Xcalibur (version 1.6.1) was used for data analysis. Verapamil was used as a positive control [48,49].

### 3.6. CYP Inhibition Assay 

Human liver microsomes (0.25 mg/mL), 0.1 M phosphate buffer (pH 7.4), a cocktail of five coenzyme substrates (Phenacetin 50 μM, Diclofenac 10 μM, S-mephenytoin 100 μM, Dextromethorphan 5 μM, Midazolam 2.5 μM), and test compound (10 μM concentration) was pre-incubated for 5 min at 37 °C. NADPH generation system solution was added and incubated for 15 min at 37 °C. In order to terminate the reaction, acetonitrile solution containing an internal standard (Terfenadine) was added and centrifuged for 5 min (14,000× *g* rpm, 4 °C). The supernatant was injected into the LC-MS/MS system to analyze the metabolites of the substrates simultaneously. Metabolites of each substrate produced during the reaction were analyzed using the Shimadzu Nexera XR system and TSQ vantage (Thermo). Kinetex C18 column (2.1 × 100 mm, 2.6 μm particle size; Phenomenex, USA) was used for HPLC. The mobile phase used contained 0.1% formic acid in distilled water (A) and 0.1% formic acid containing acetonitrile (B). The generated metabolites were quantified using MRM (Multiple Reaction Monitoring) and Xcalibur (version 1.6.1) was used for data analysis [50,51].

### 3.7. Computational Docking Simulation

Ligand binding site for docking was defined as a 30 Å^3^ grid box for DYRK1A and 20 × 24 × 20 Å^3^ grid box for TRKA centered on the centroid of co-crystallized native ligands. The crystal structures of DYRK1A (PDB ID: 7A51) [46] and TRKA (PDB ID: 5JFV) [47] were downloaded from RCSB PDB and computational docking was performed using AutoDock Vina version 1.5.6 (The Scripps Research Institute, La Jolla, CA, USA) [52]. For the macromolecule-ligand pair, the binding model of the ligand with the lowest binding free energy (kcal/mol) was used for further analysis. Figure to show the molecular modeling results were visualized using PyMOL (Schrödinger, LLC, New York, NY, USA) [53]. LIGPLOT^+^ (version 2.2.4) was used to view the interactions between amino acid residues of enzyme and compound [54].

## 4. Conclusions

Protein kinases represent a promising target for the development of anticancer agents due to their association with cancer growth and progression [10,11,12]. In the present study, we designed molecules using the nucleoside skeleton with the intention to simultaneously occupy the hinge and the hydrophobic region I (buried region), along with the ribose region of the ATP-binding site. We sought to identify whether the hydrophobic pocket I acts as a pharmacophore in kinase inhibition. Thus, we designed and synthesized 7-substituted 7-deaza-4′-thioadenosine derivatives **1** with a nucleoside skeleton by modifying the hydrophobic residue (R), based on ATP-kinase interactions. Among all the synthesized compounds, compound **1g** with acetylene at the 7-position of 7-deaza-4′-thioadenosine (R = acetylene) exhibited markedly potent anticancer activity in vitro against six different cancer cell lines and potent kinase inhibition of TRKA, DYRK1A/1B, and CK1δ at a concentration of 1 μM among the panel of 96 kinases. The results showed that the C-7 substituent of 7-deazaadenine was optimal for substituting extremely small and linear acetylene, indicating that a very small linear hydrophobic group is required to inhibit TRKA, DYRK1A/1B, and CK1δ. These results will contribute greatly to the further development of new anticancer agents with multi-kinase inhibition.

## Figures and Tables

**Table 1:**
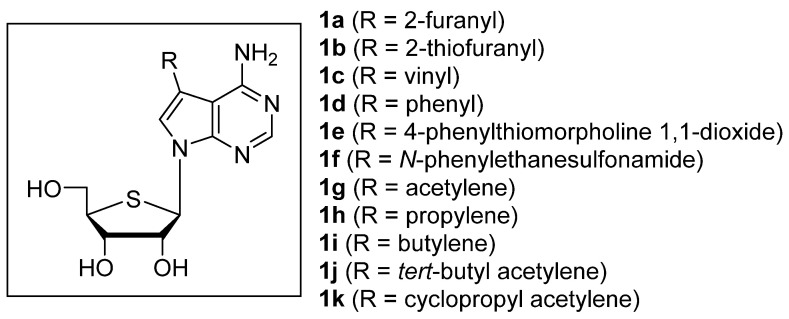
Anticancer activity of the final 7-substituted 7-deaza-4′-thionucleosides **1a**–**k** against several human cancer cell lines.

Compound No.	IC_50_ (μM) *^a^*					
	A549 *^b^*	HCT116 *^c^*	MDA-MB-231 *^d^*	SK-HEP-1 *^e^*	SNU638 *^f^*	PC-3 *^g^*
**1a**	1.02	2.16	3.31	3.38	1.95	2.46
**1b**	1.65	1.74	2.72	1.38	2.41	2.48
**1c**	0.97	0.56	0.31	0.22	0.47	0.2
**1d**	7.57	6.35	8.83	7.81	6.32	9.21
**1e**	>50	>50	>50	>50	>50	>50
**1f**	41.6	30.6	30.1	10.3	27.5	15.4
**1g**	0.06	0.03	0.05	0.05	0.03	0.004
**1h**	>50	>50	45.5	6.05	>50	>50
**1i**	>50	>50	>50	>50	>50	>50
**1j**	>50	>50	29.6	>50	>50	>50
**1k**	>50	>50	30.6	8.31	>50	>50
Etoposide *^h^*	0.36	1.11	4.9	0.91	0.41	23.4
Gemcitabine *^i^*	0.3	0.2	1.1	0.2	0.1	3.6

*^a^* Measured using SRB assay. *^b^* Human lung cancer cells. *^c^* Human colon cancer cells. *^d^* Human breast cancer cells. *^e^* Human liver cancer cells. *^f^* Human stomach cancer cell. *^g^* Human prostate cancer cells. *^h^* Etoposide was used as positive control. *^i^* Gemcitabine was used as positive control [44].

**Table 2:** Human liver microsomal stability and CYP isozyme inhibition activity of **1g**.

Compound No.	Metabolic Stability (%) *^a^*	CYP Inhibition, IC_50_ (μM) *^b^*				
		CYP1A2	CYP2C9	CYP2C19	CYP2D6	CYP3A4
**1g**	82.3	>20	>20	>20	>20	>20
Verapamil	15.3	-	-	-	-	-
Ketoconazole *^d^*	-	95.7 *^c^*	93.6 *^c^*	93.6 *^c^*	96.0 *^c^*	27.2 *^c^*

*^a^* % Remaining during 30 min. *^b^* IC_50_ < 1 μM—potent inhibition; 1 μM < IC_50_ < 10 μM—moderate inhibition; IC_50_ > 10 μM—no or weak inhibition. *^c^* Percentage of enzyme remaining after inhibition. *^d^* CYP3A4 inhibitor (0.1 μM).

## Data Availability

Data is contained within the article and supplementary files.

## Supplementary Materials

The following are available online at https://www.mdpi.com/article/10.3390/ph14121290/s1, ^1^H and ^13^C NMR spectra, HRMS (ESI-Q-TOF) data for **1g**, Figure S1: ORTEP diagram of compound **1g** showing thermal ellipsoid at 50% probability, X-ray crystallographic data for **1g**, HPLC chromatograms, Table S1: Kinome scan data of compound **1g**, Table S2: Kinase inhibition profile of **1a**–**k** against TRKA, CK1δ, and DYRK1A/1B.
