# Supplementary material for: Discovery of a Novel Template, 7-Substituted 7-Deaza-4′-Thioadenosine Derivatives as Multi-Kinase Inhibitors
Source: Pharmaceuticals (Basel). 2021 Dec 10;14(12):1290. doi: 10.3390/ph14121290 (PMC8708872; doi:10.3390/ph14121290)
Supplement: Supplementary file 1 [file pharmaceuticals-14-01290-s001.zip › pharmaceuticals-1438528-supplementary.pdf]

Supporting information

Discovery of a Novel Template, 7-Substituted 7-Deaza-4'-thioadenosine Derivatives as Multi-kinase Inhibitors

Karishma K. Mashelkar[1], Woong Sub Byun[1], Hyejin Ko[1], Kisu Sung[1], Sushil K. Tripathi[1], Seungchan An[1], Yun A Yum[1], Jee Youn Kwon[1], Minjae Kim[1], Gibae Kim[1], Eun-Ji Kwon,[1] Hyuk Woo Lee[2], Minsoo Noh[1], Sang Kook Lee[1], and Lak Shin Jeong[1,*]

[1]Research Institute of Pharmaceutical Sciences, College of Pharmacy, Seoul National University, Seoul 08826, Korea and [2]Future Medicine Co., Ltd, Seongnam, Gyeonggi-do, Korea,

* To whom correspondence should be addressed.

E-mail: lakjeong@snu.ac.kr

Contents

^{1}H and ^{13}C NMR Spectra……………………………………………………………………….S02-S59
HRMS (ESI-Q-TOF) data for compound 1g…………………………………..……………….…….S60
X-ray Crystallographic data for compound 1g…………………..………………...…………………….S61-S63
HPLC Chromatograms………………….....……………………………...…………………………..S64-S74
Kinome scan data of compound 1g………………………………………………………………..........S75-S78
Kinase inhibition profile of 1a-k against TRKA, CK1δ, and DYRK1A/1B (Table S2)……………………………..S79

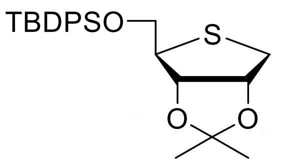

7

^{1}H NMR (500 MHz, $CDCl_3$)

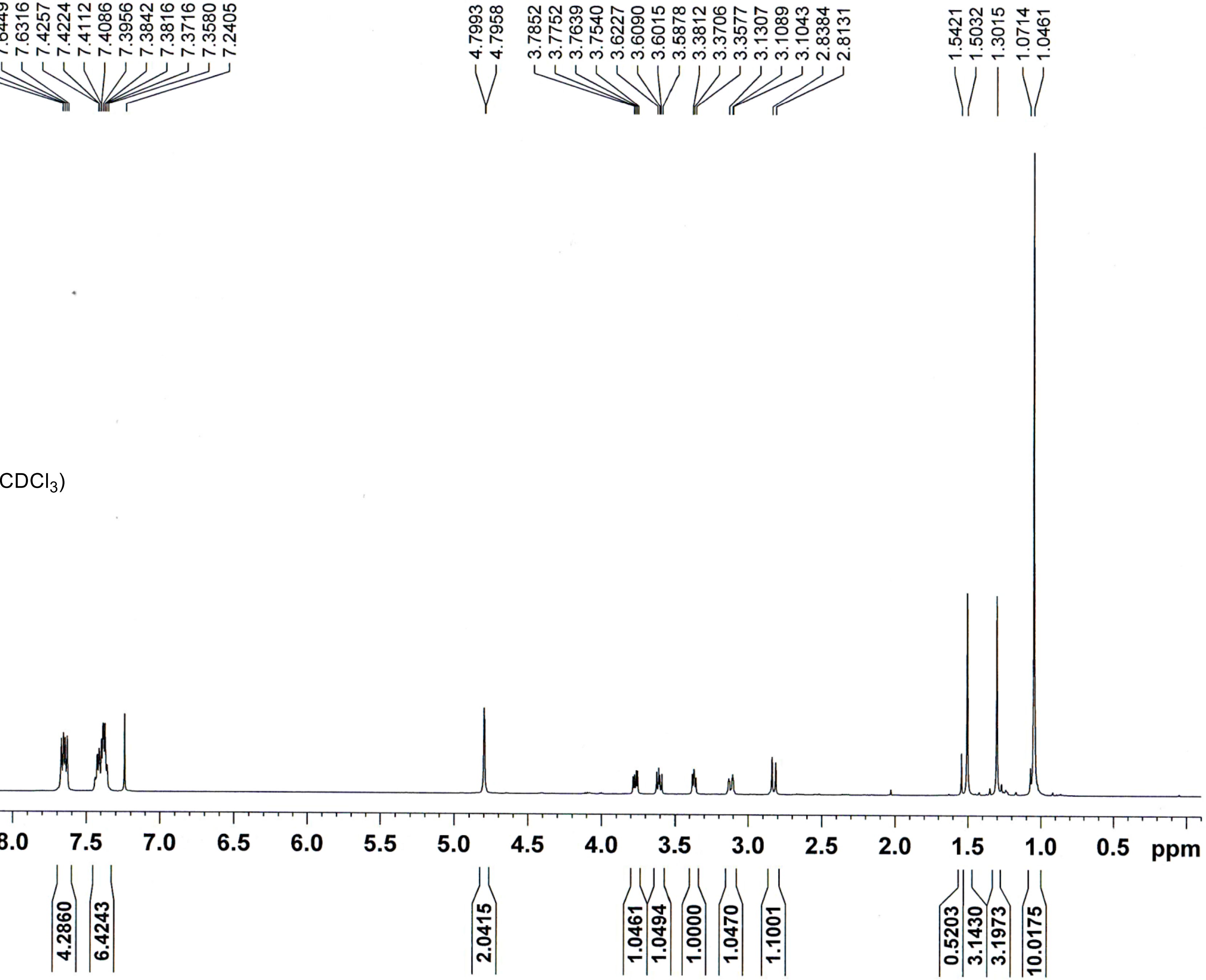

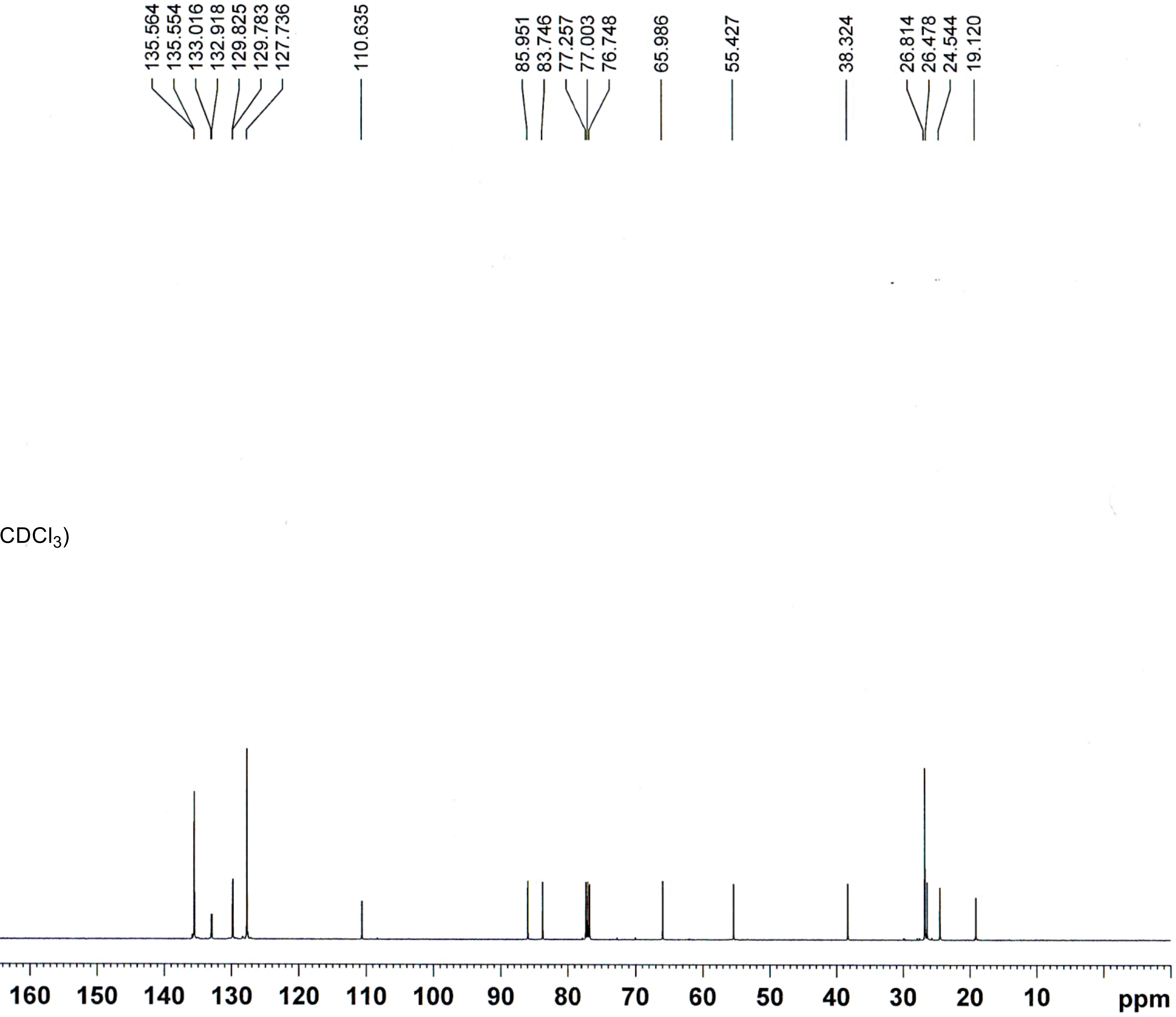

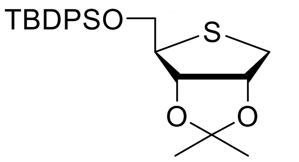

7

^{13}C NMR (125 MHz, $CDCl_3$)

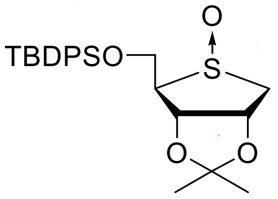

8

^1H NMR (500 MHz, $CDCl_3$)

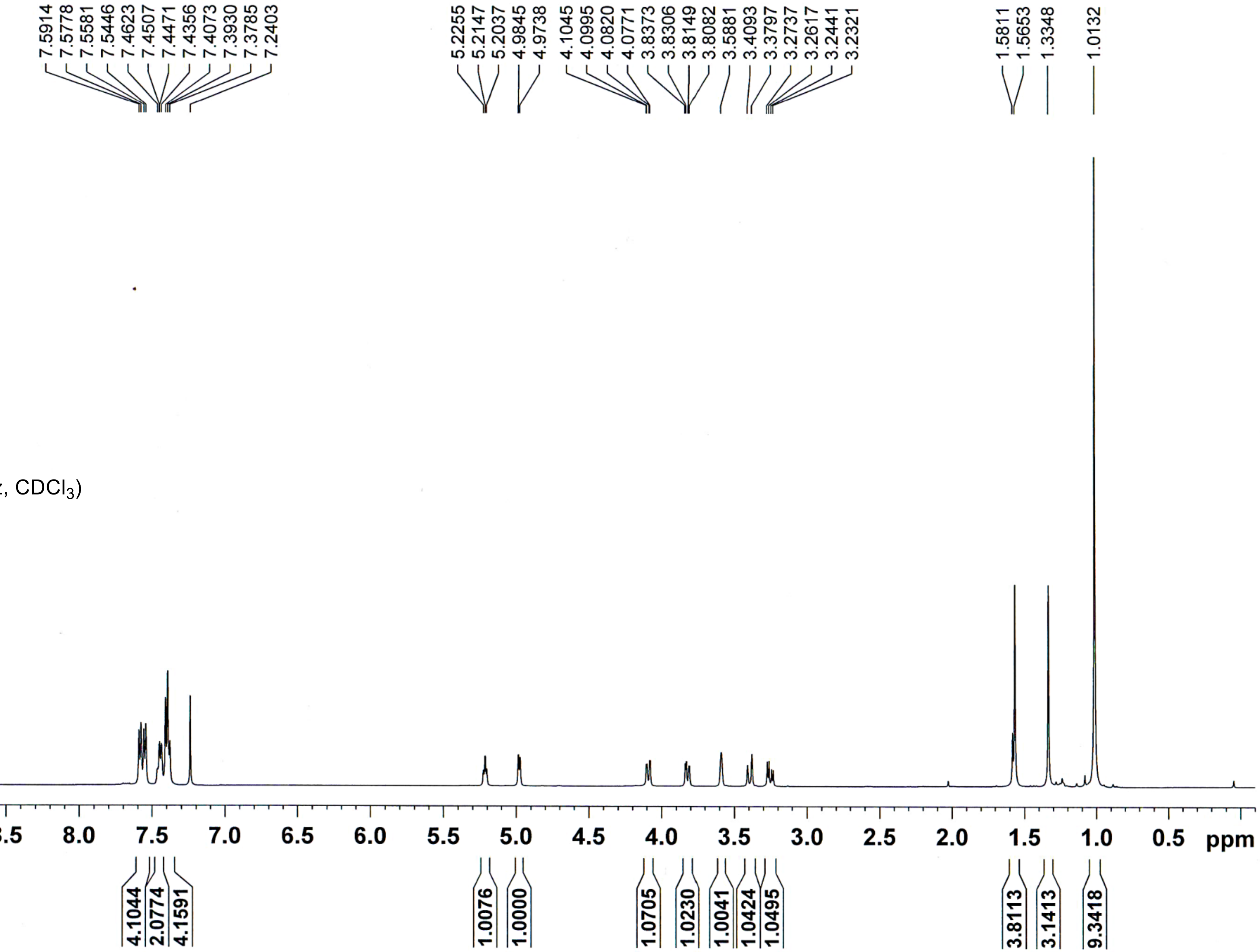

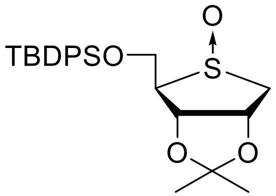

8

^{13}C NMR (125 MHz, $CDCl_3$)

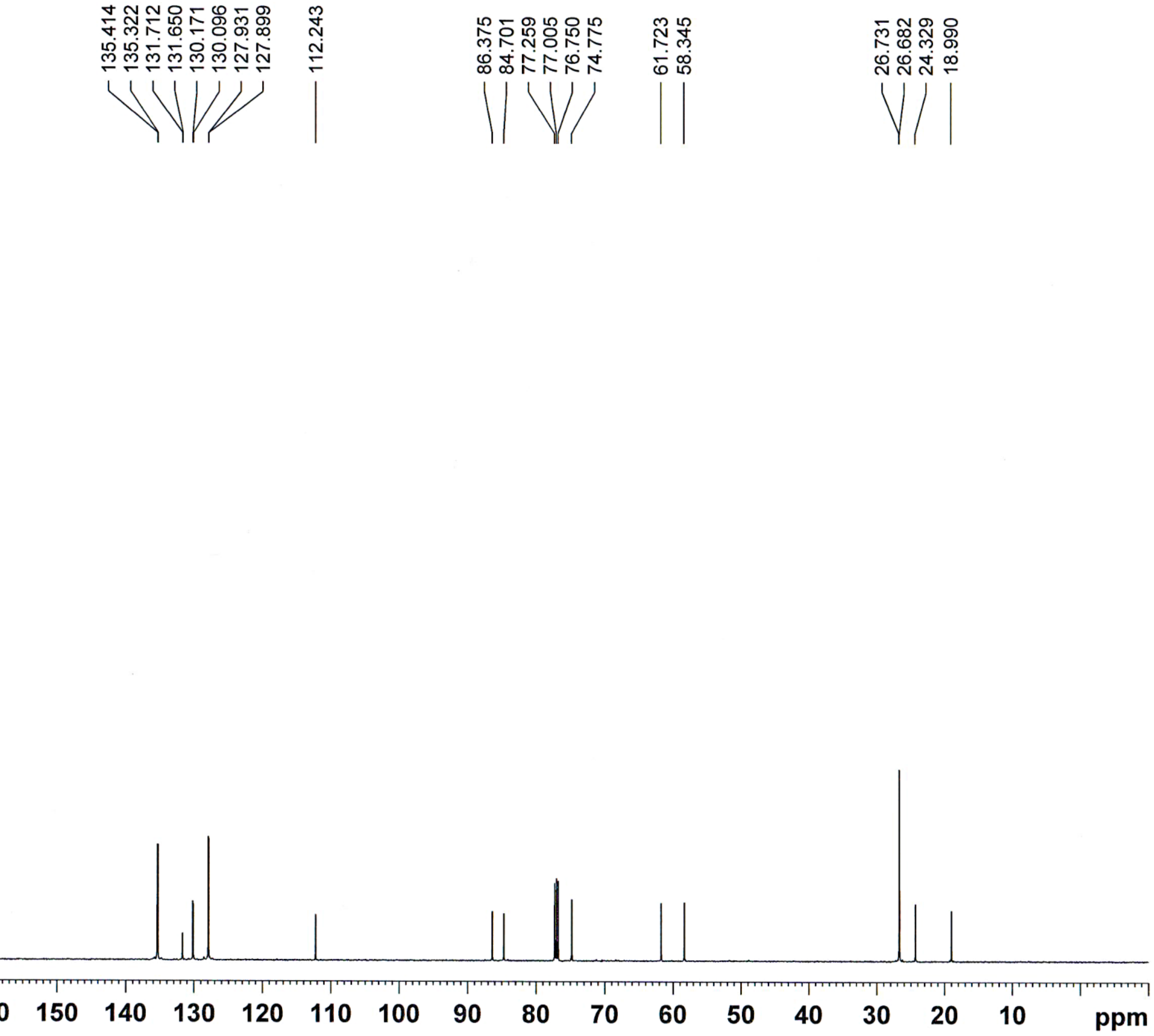

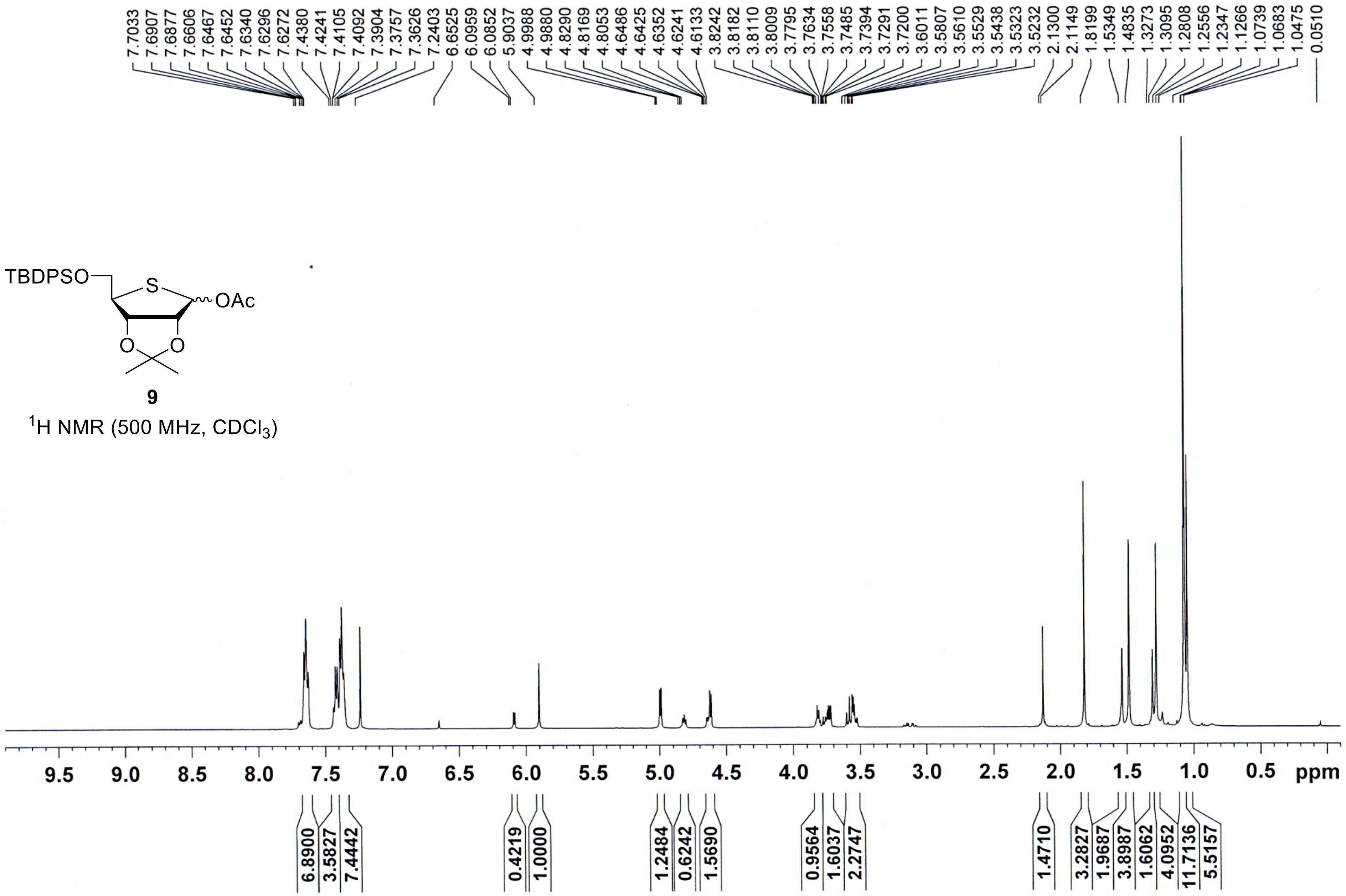

9

^{1}H NMR (500 MHz, $CDCl_3$)

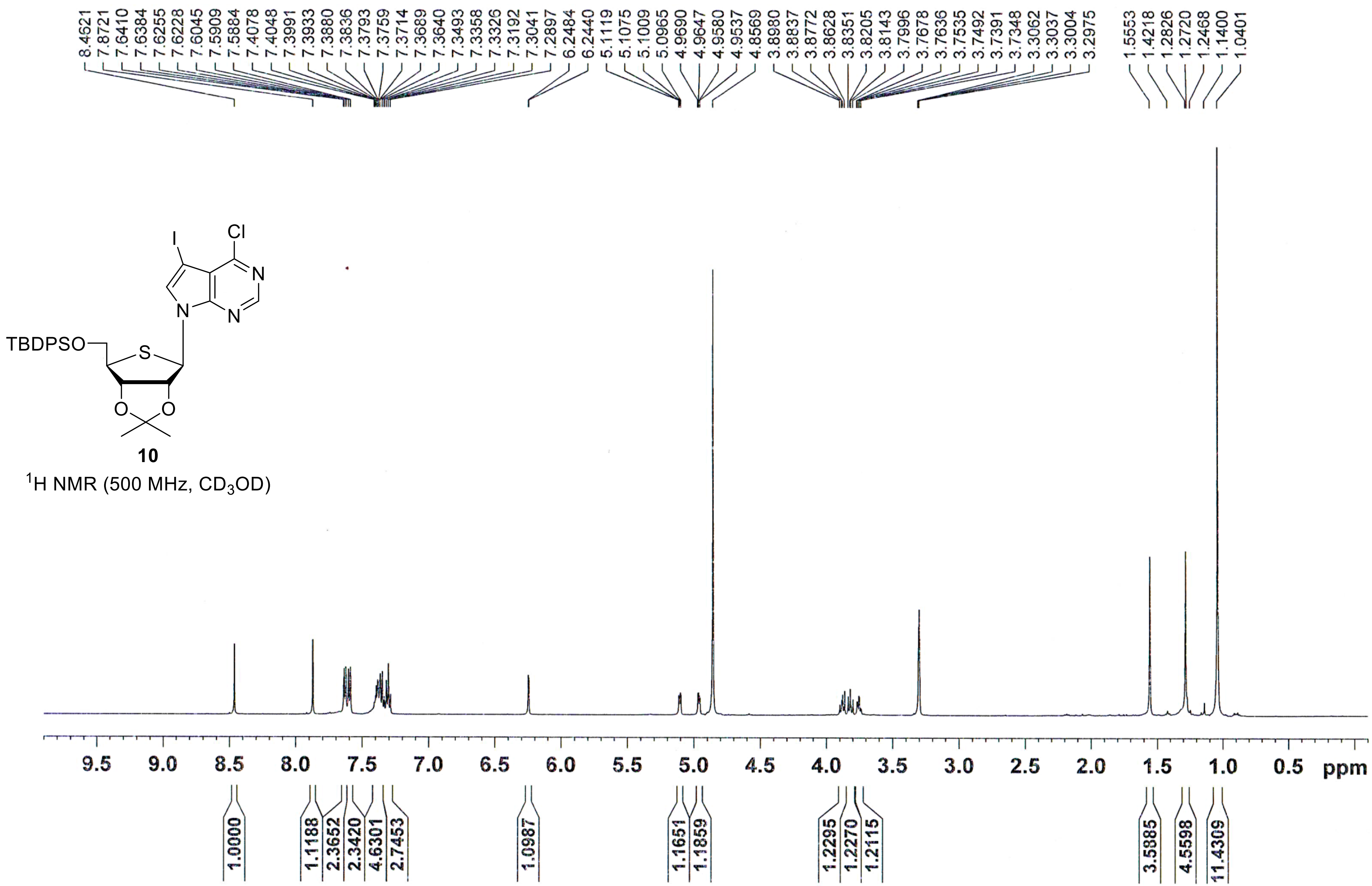

8.4521
7.8721
7.6410
7.6384
7.6255
7.6228
7.6045
7.5909
7.5884
7.4078
7.4048
7.3991
7.3933
7.3880
7.3836
7.3793
7.3759
7.3714
7.3689
7.3640
7.3493
7.3358
7.3326
7.3192
7.3041
7.2897
6.2484
6.2440
5.1119
5.1075
5.1009
5.0965
4.9690
4.9647
4.9580
4.9537
4.8569
3.8980
3.8837
3.8772
3.8628
3.8351
3.8205
3.8143
3.7996
3.7678
3.7636
3.7535
3.7492
3.7391
3.7348
3.3062
3.3037
3.3004
3.2975
1.5553
1.4218
1.2826
1.2720
1.2468
1.1400
1.0401
I
Cl
N
N
N
TBDPSO
S
O
O
10
^{1}H NMR (500 MHz, CD_3OD)
9.5
9.0
8.5
8.0
7.5
7.0
6.5
6.0
5.5
5.0
4.5
4.0
3.5
3.0
2.5
2.0
1.5
1.0
0.5
ppm
1.0000
1.1188
2.3652
2.3420
4.6301
2.7453
1.0987
1.1651
1.1859
1.2295
1.2270
1.2115
3.5885
4.5598
11.4309

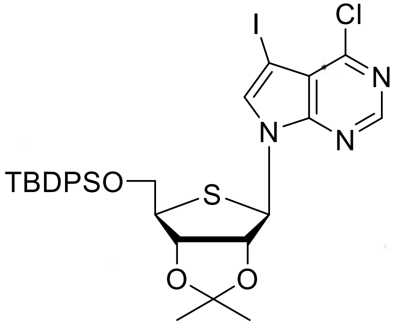

10

^{13}C NMR (100 MHz, CD_3OD)

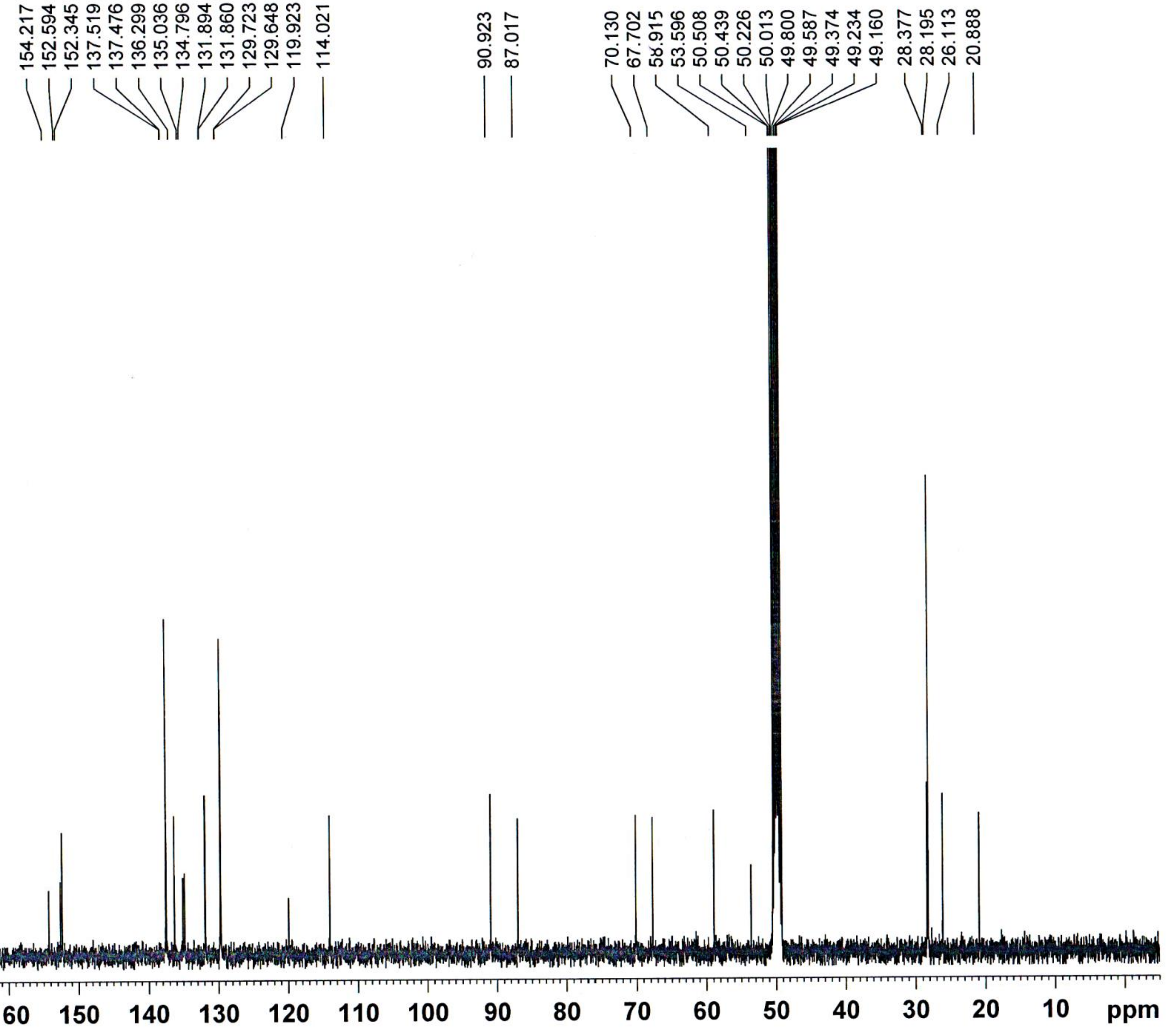

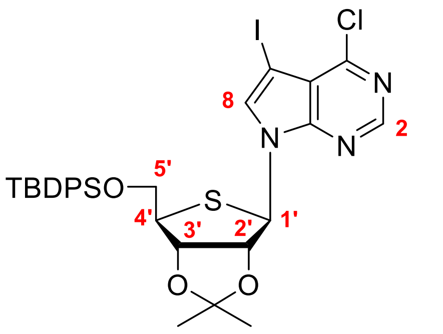

10

^{1}H-^{1}H NOESY (600 MHz, CD_3OD)

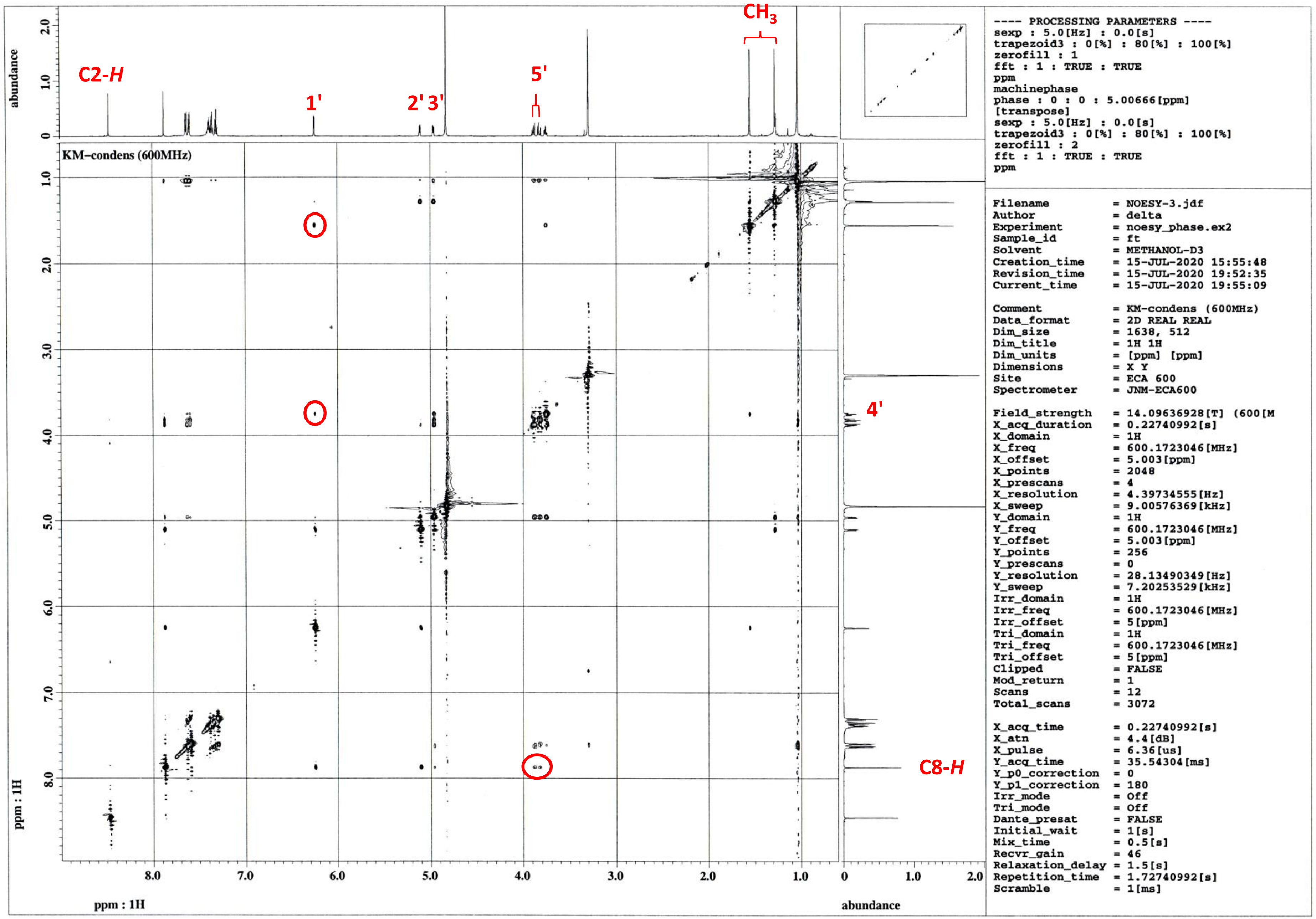

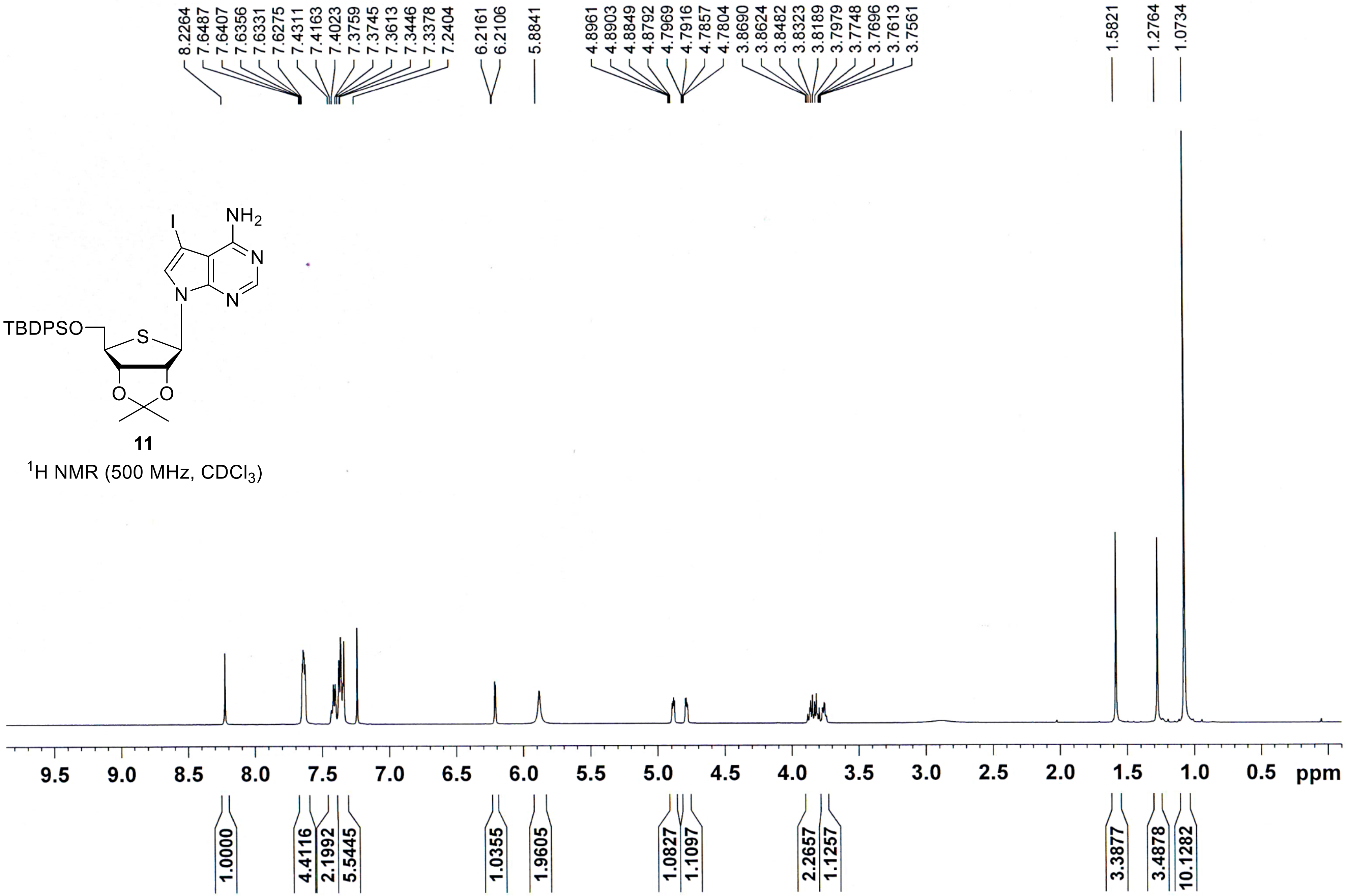

11

^{1}H NMR (500 MHz, $CDCl_3$)

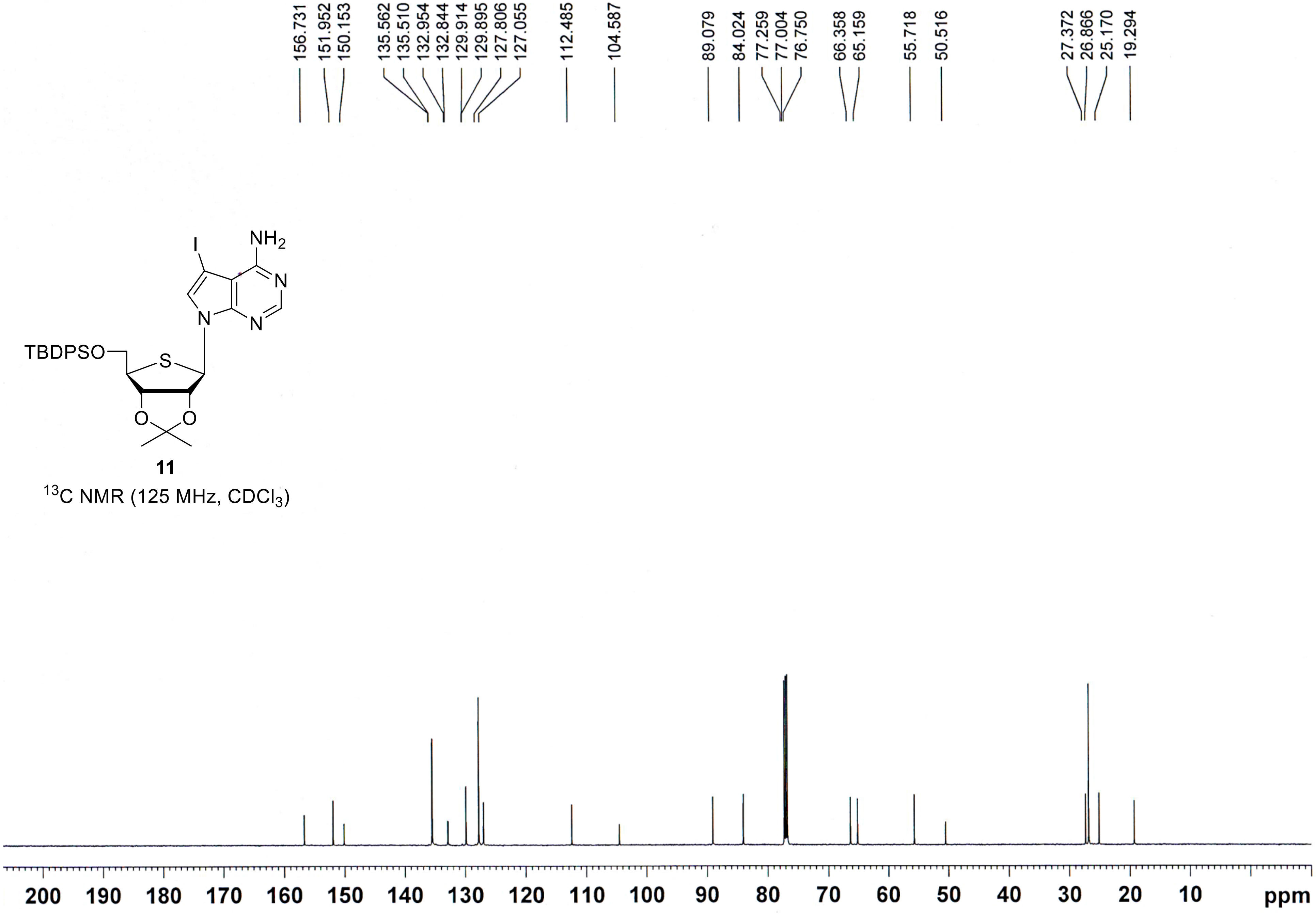

11

^{13}C NMR (125 MHz, $CDCl_3$)

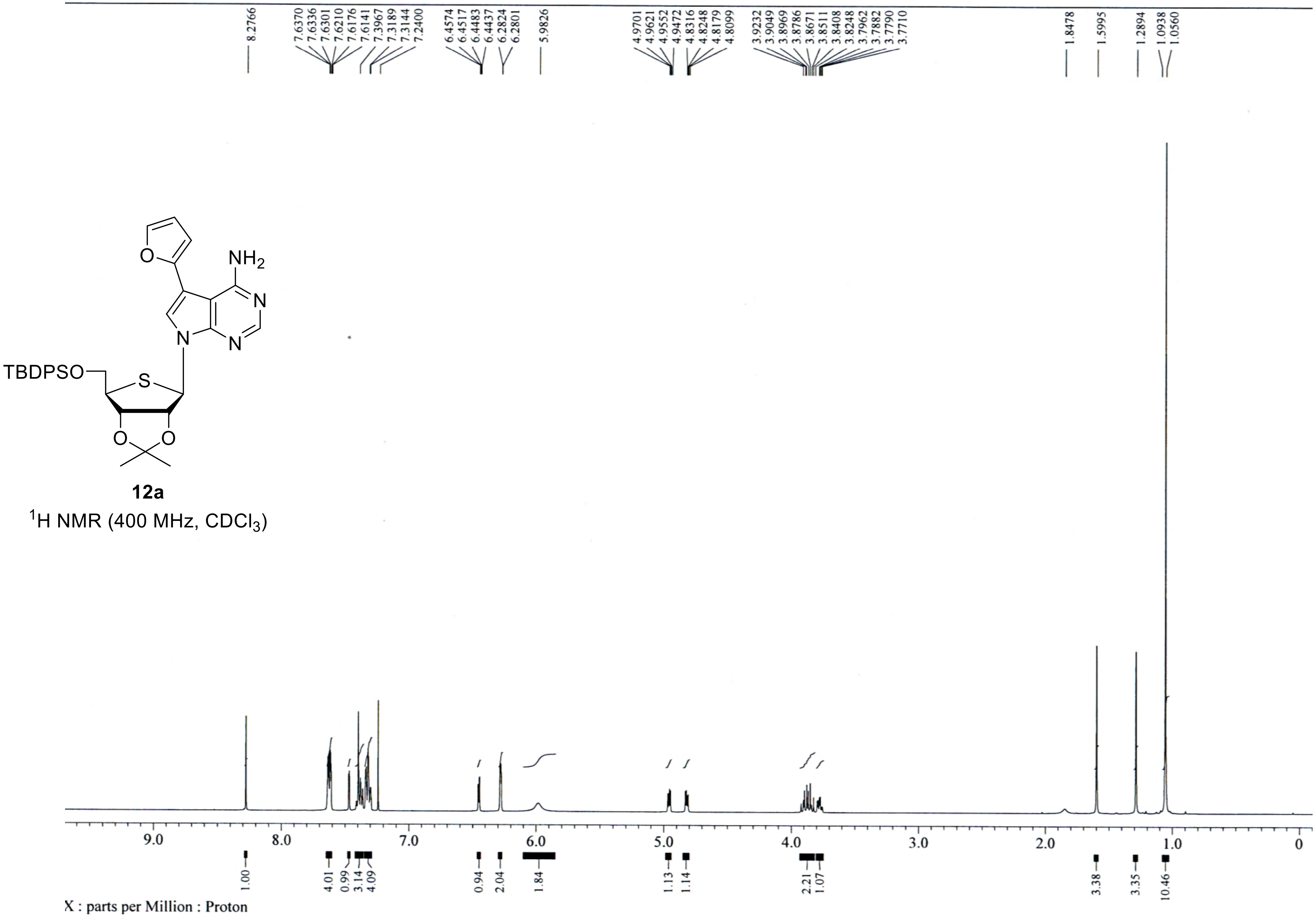

12a

^{1}H NMR (400 MHz, $CDCl_3$)

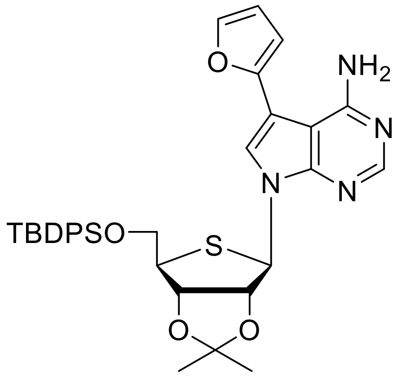

12a

^{13}C NMR (100 MHz, $CDCl_3$)

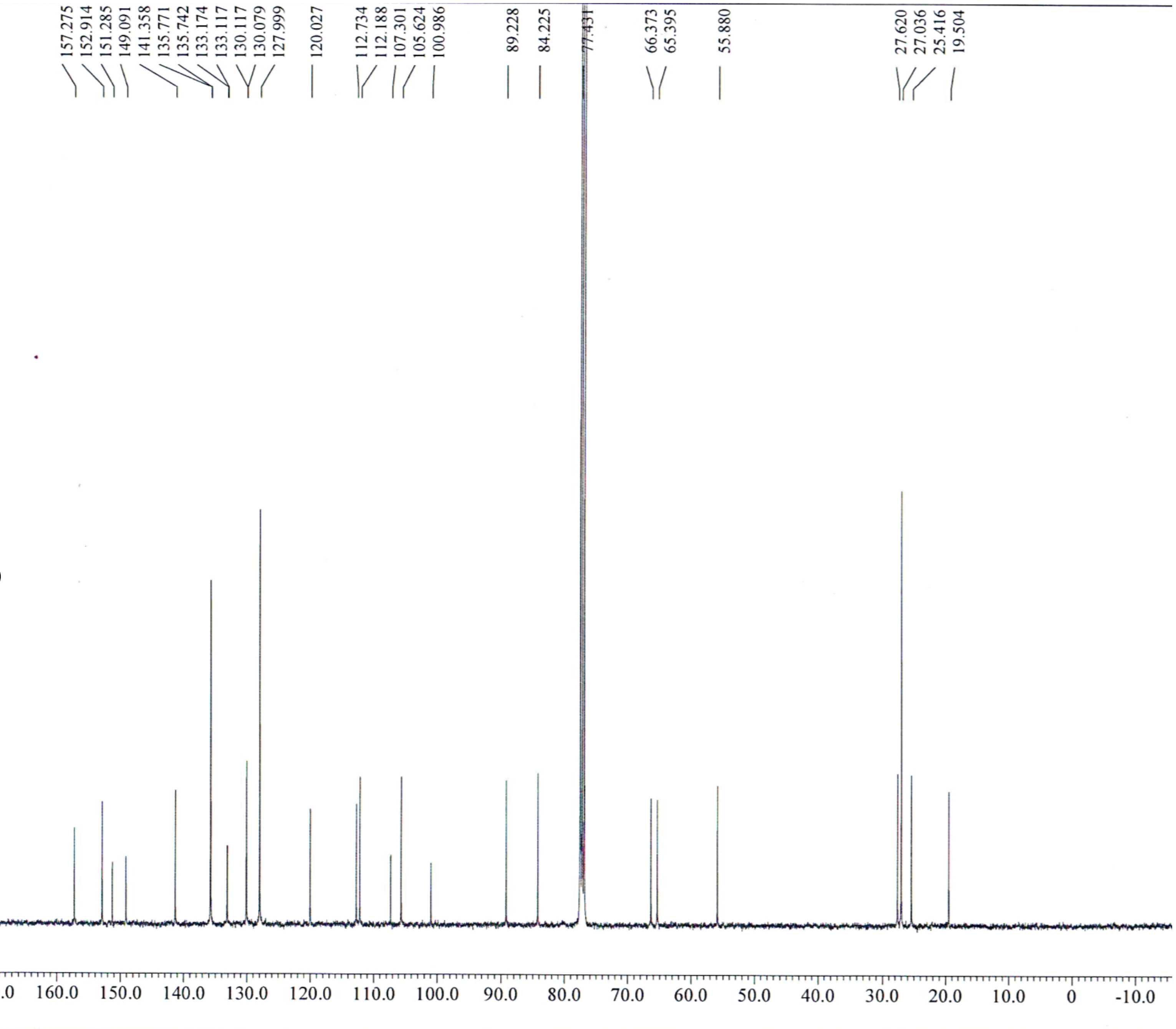

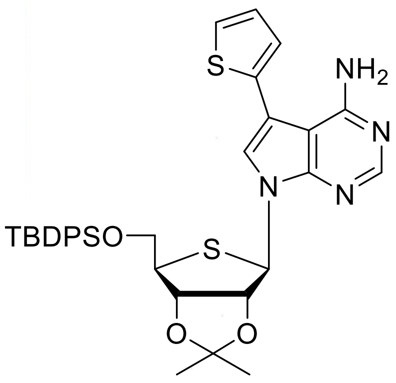

12b

^{1}H NMR (400 MHz, $CDCl_3$)

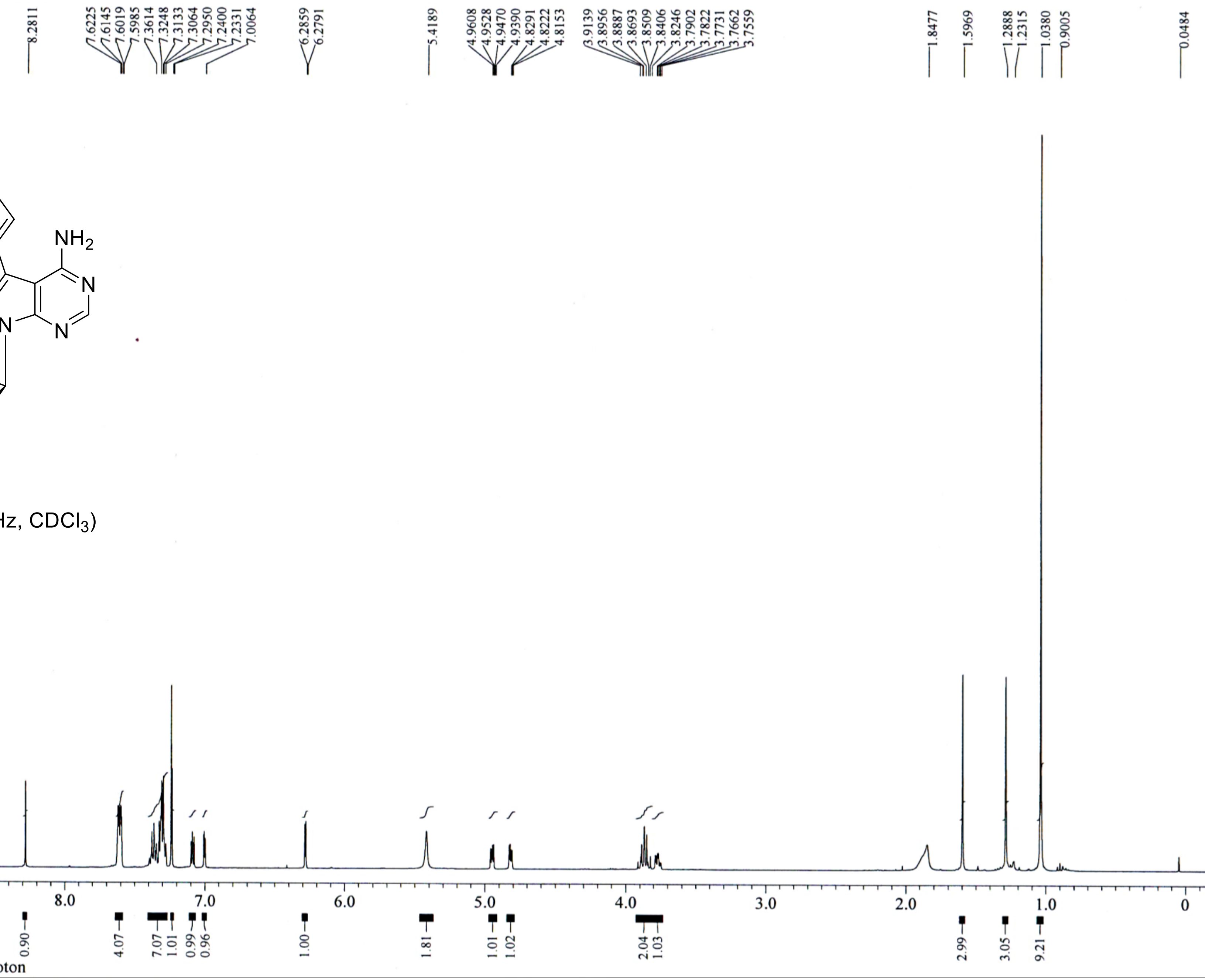

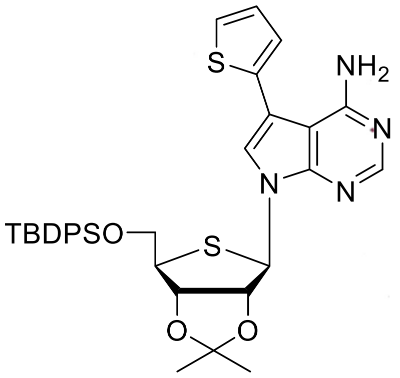

12b

^{13}C NMR (100 MHz, $CDCl_3$)

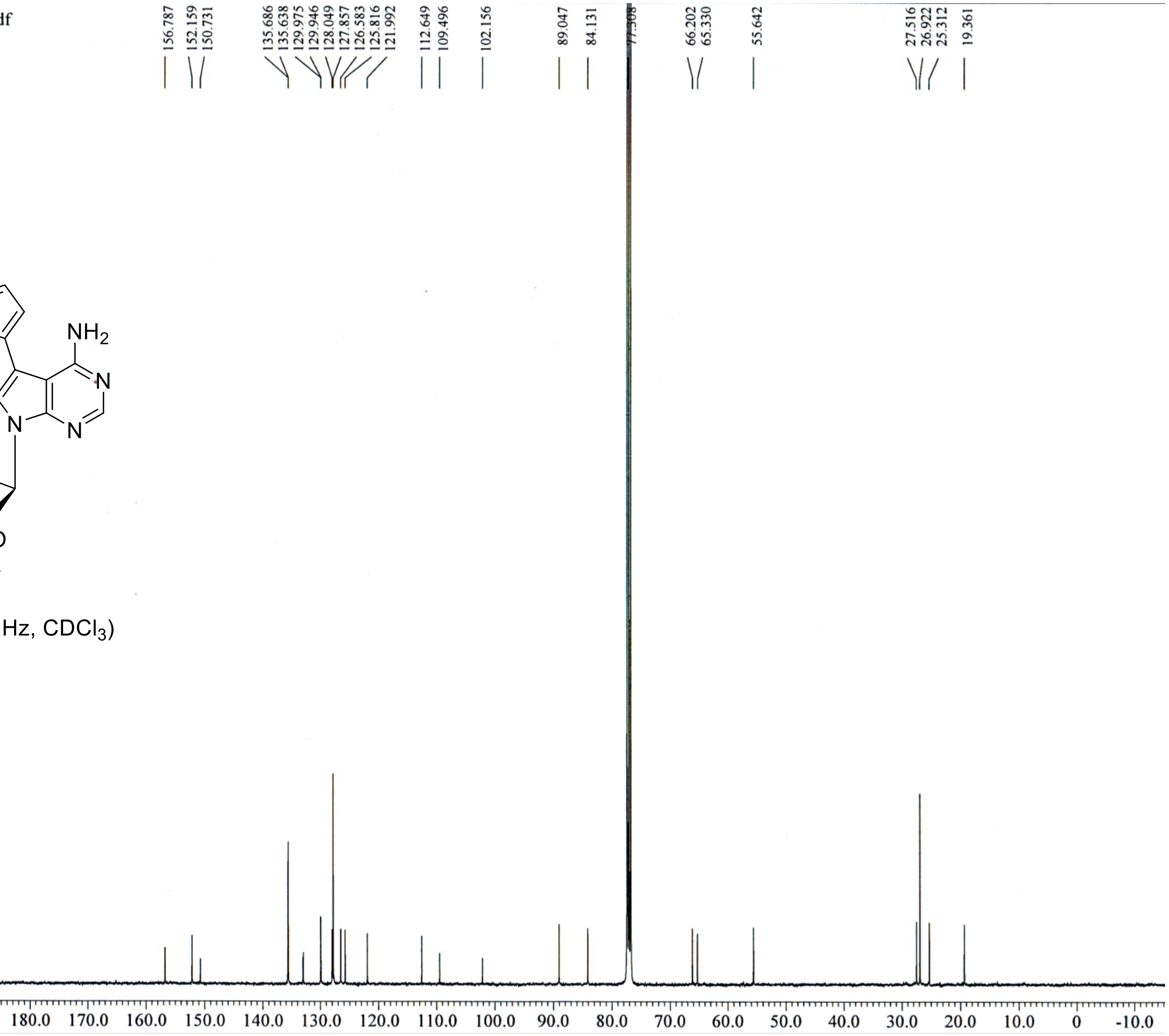

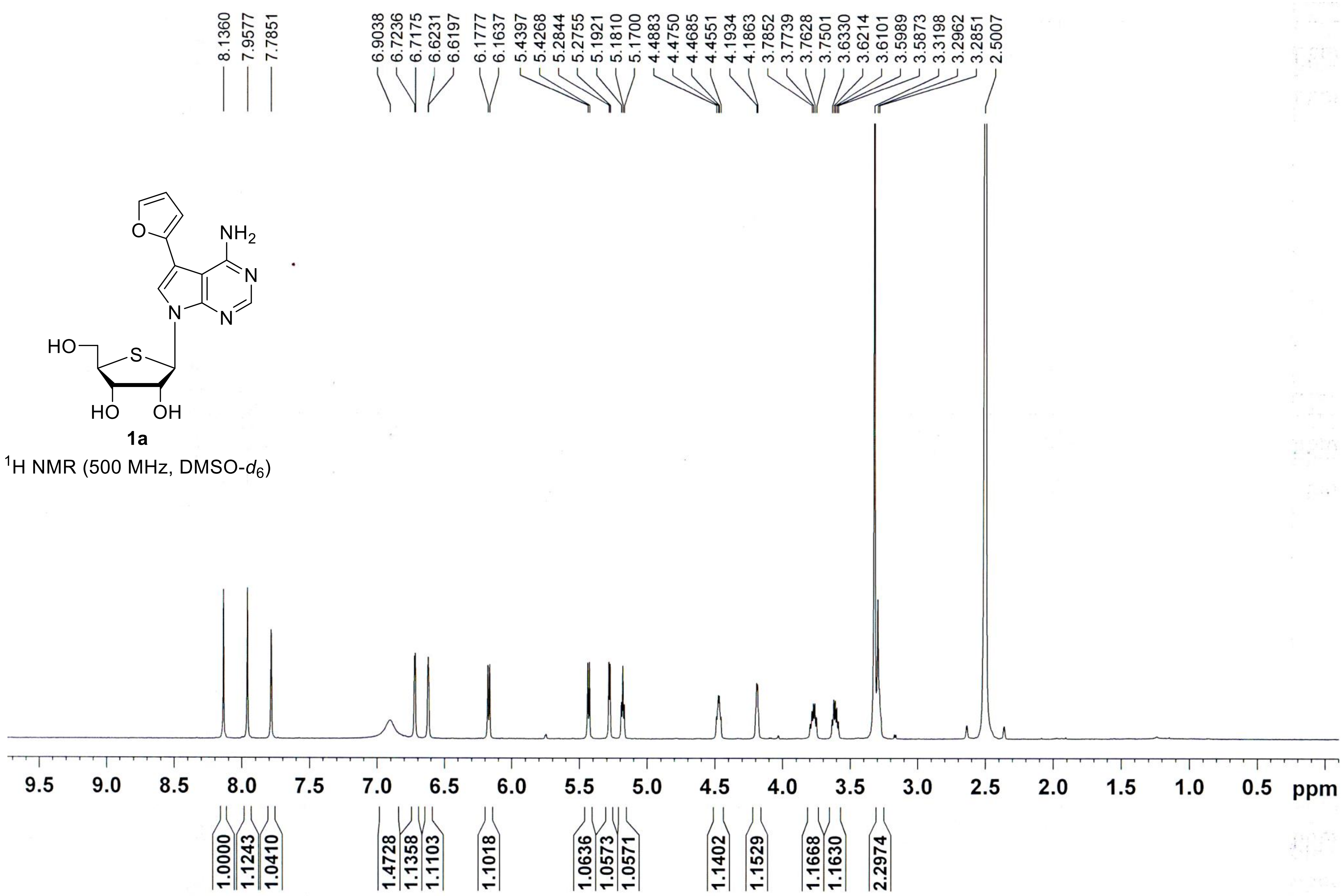

1a

^{1}H NMR (500 MHz, DMSO-d_6)

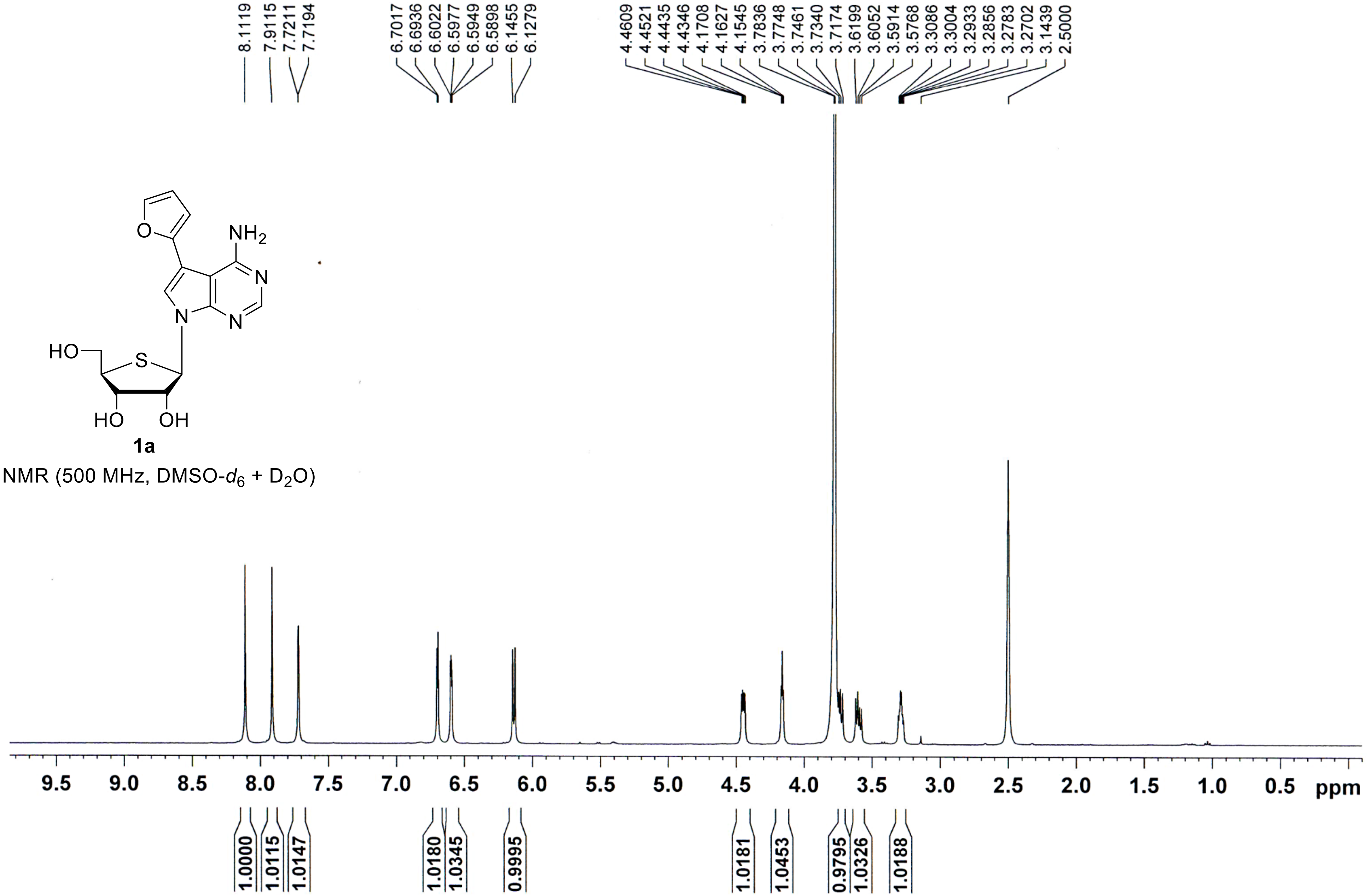

1a

^{1}H NMR (500 MHz, DMSO-d_6 + D_2O)

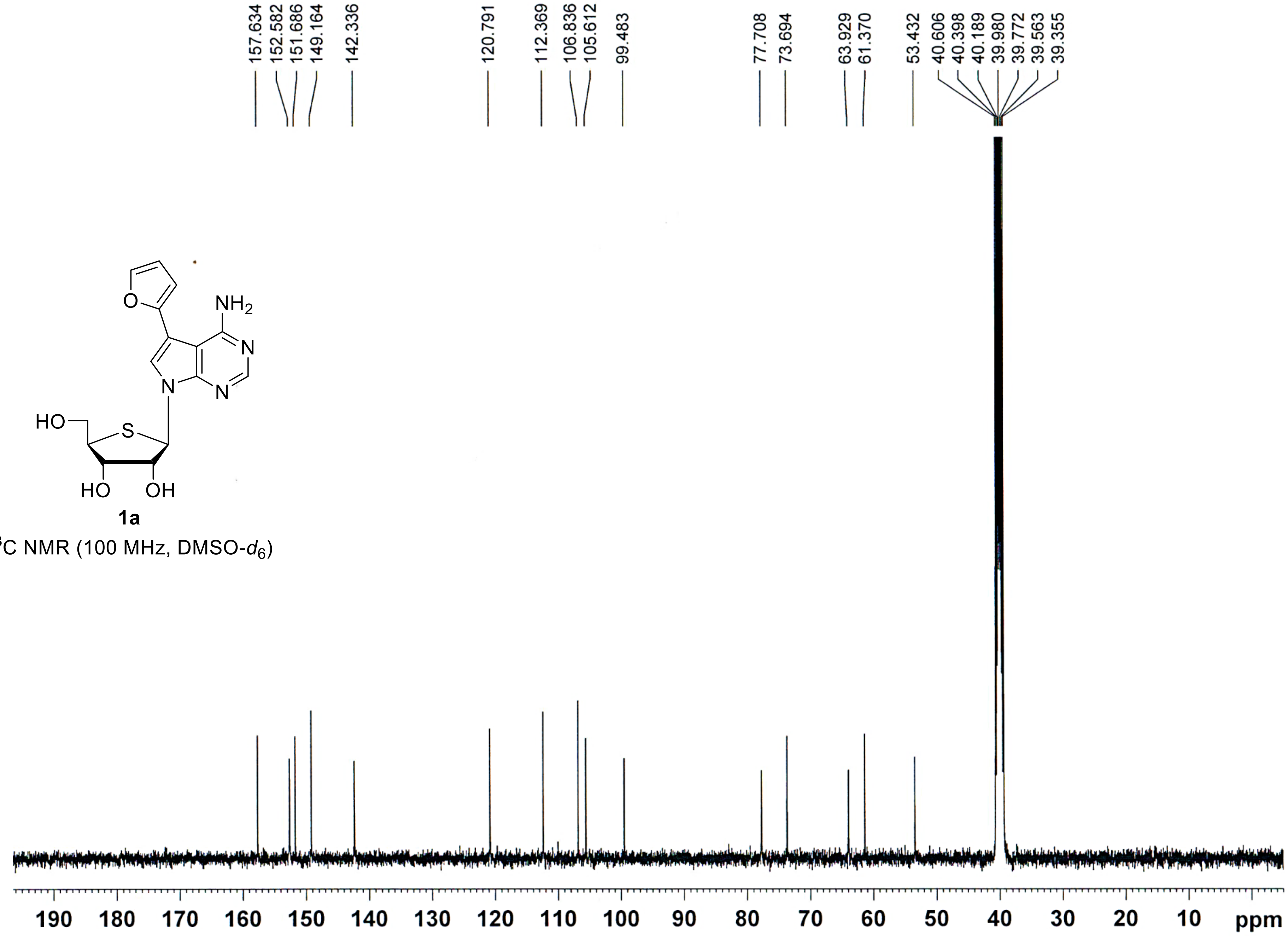

1a

^{13}C NMR (100 MHz, DMSO-*d*$_6$)

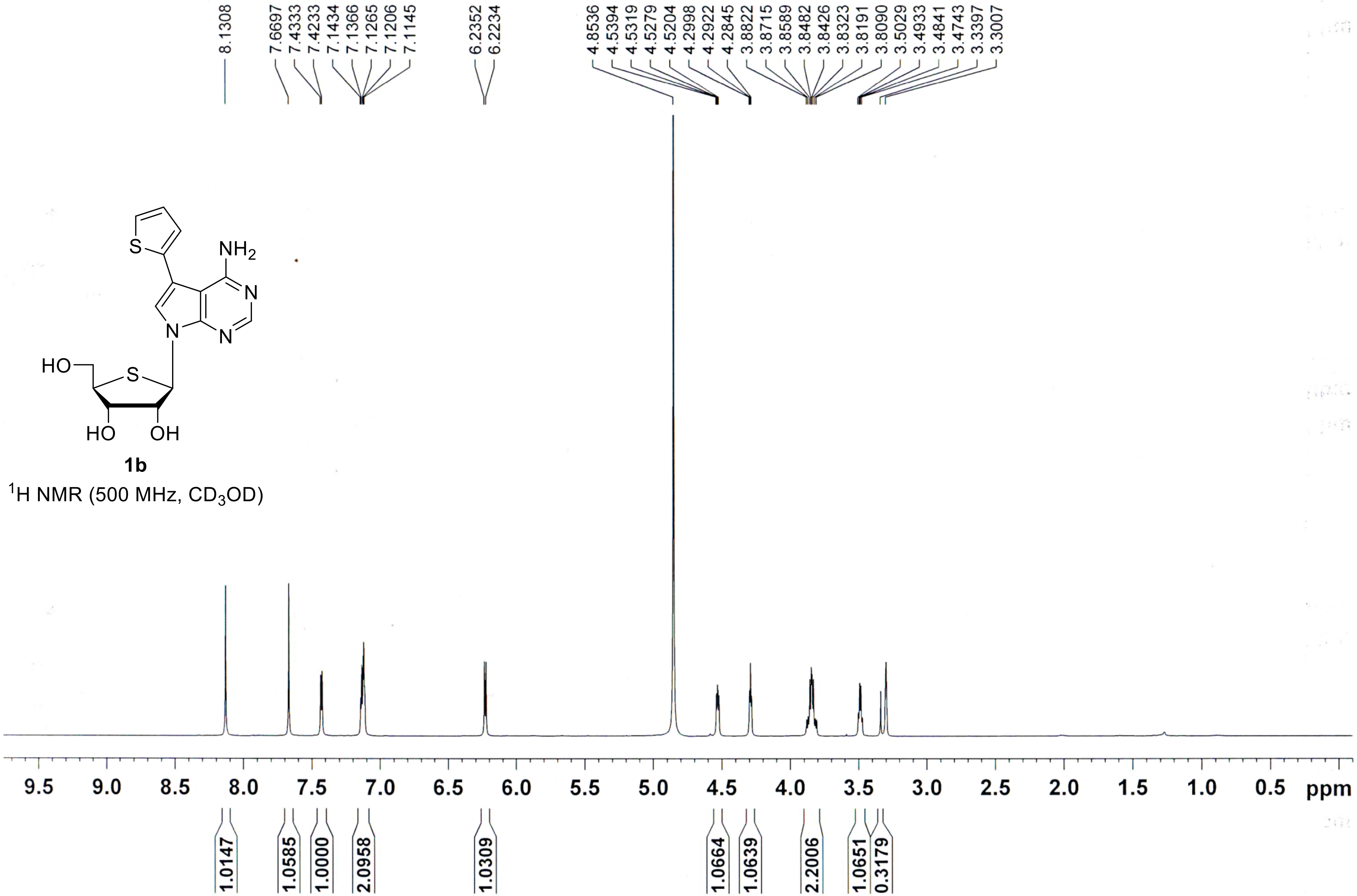
8.1308
7.6697
7.4333
7.4233
7.1434
7.1366
7.1265
7.1206
7.1145
6.2352
6.2234
4.8536
4.5394
4.5319
4.5279
4.5204
4.2998
4.2922
4.2845
3.8822
3.8715
3.8589
3.8482
3.8426
3.8323
3.8191
3.8090
3.5029
3.4933
3.4841
3.4743
3.3397
3.3007
NH2
N
N
N
S
HO
S
HO
OH
1b
1H NMR (500 MHz, CD3OD)
9.5 9.0 8.5 8.0 7.5 7.0 6.5 6.0 5.5 5.0 4.5 4.0 3.5 3.0 2.5 2.0 1.5 1.0 0.5 ppm
1.0147
1.0585
1.0000
2.0958
1.0309
1.0664
1.0639
2.2006
1.0651
0.3179

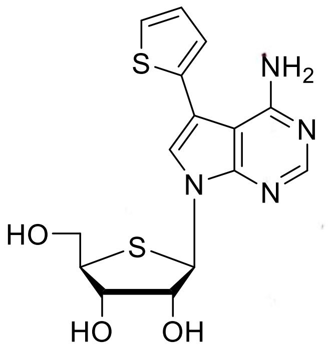

1b

^{13}C NMR (125 MHz, CD_3OD)

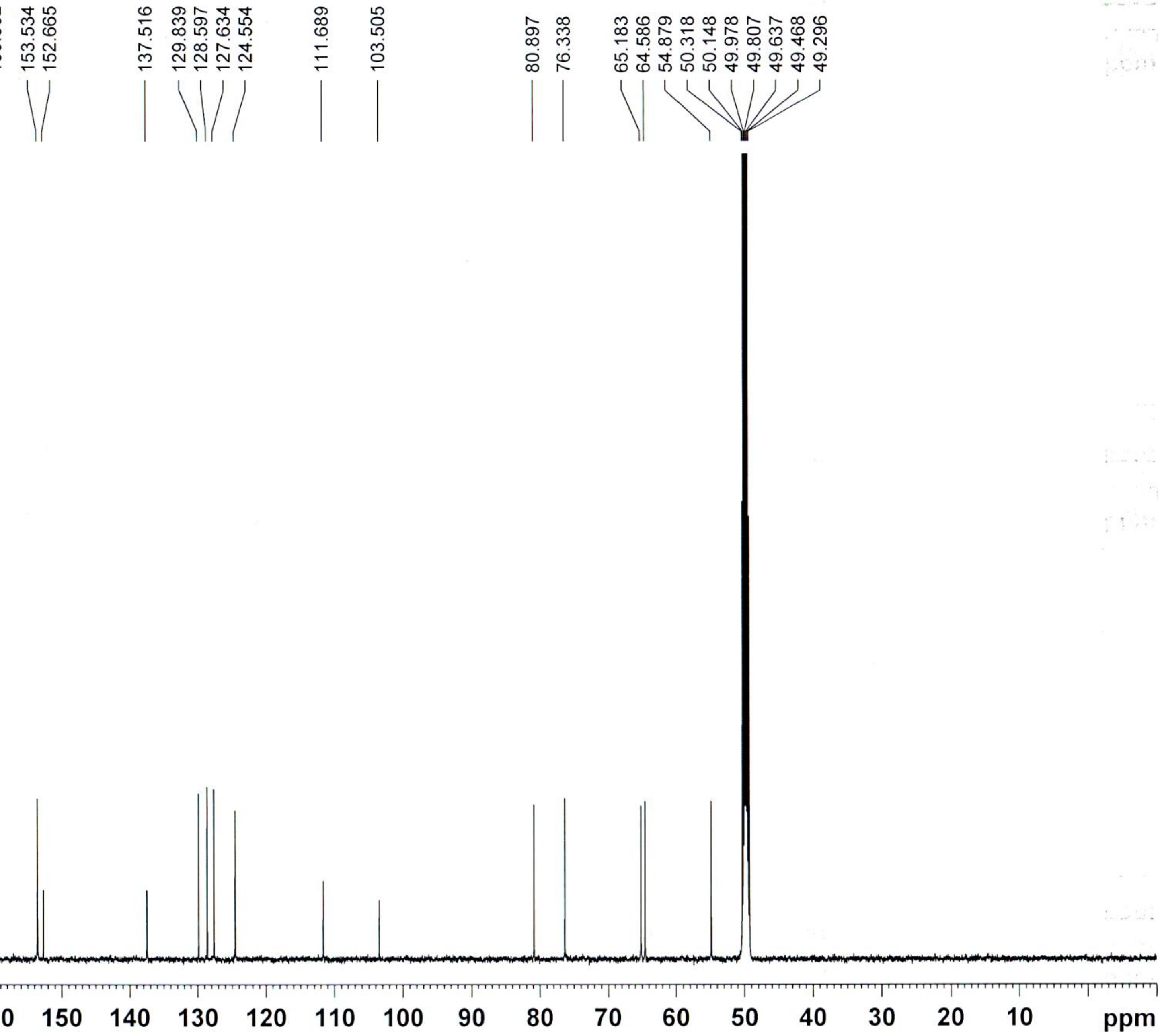

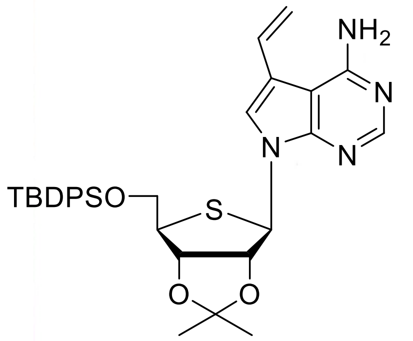

12c

^{1}H NMR (400 MHz, CD_3OD)

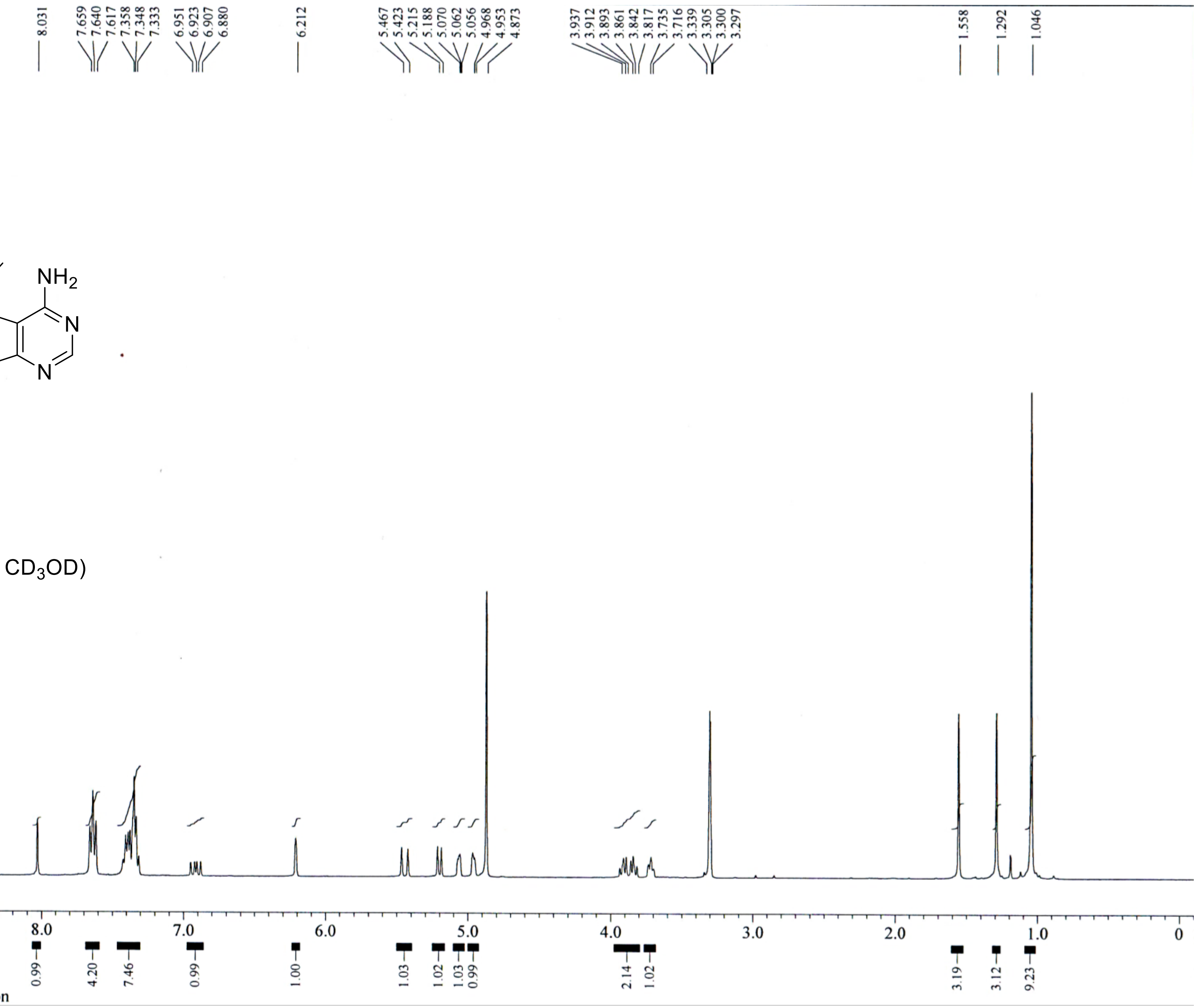

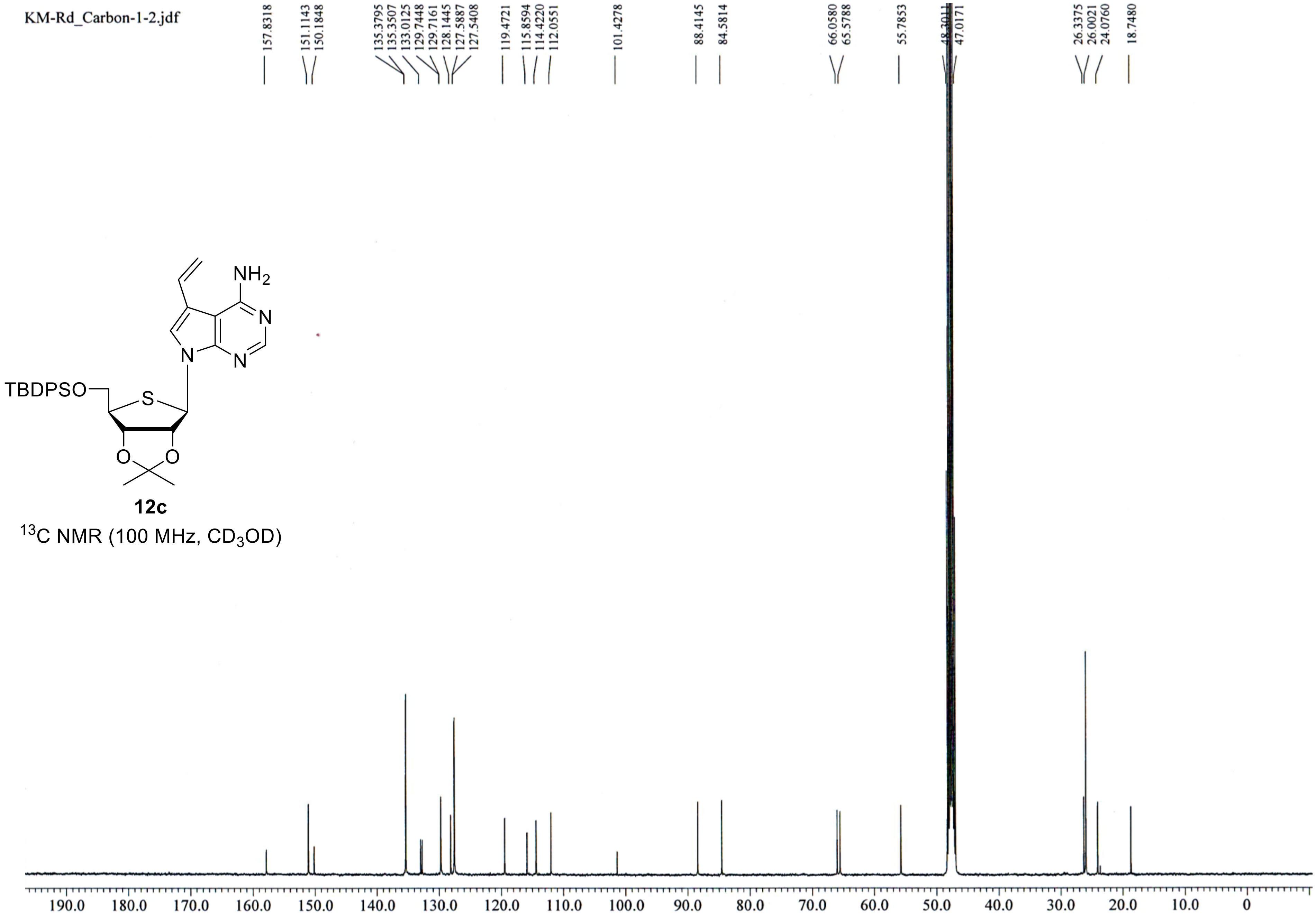

12c

^{13}C NMR (100 MHz, CD_3OD)

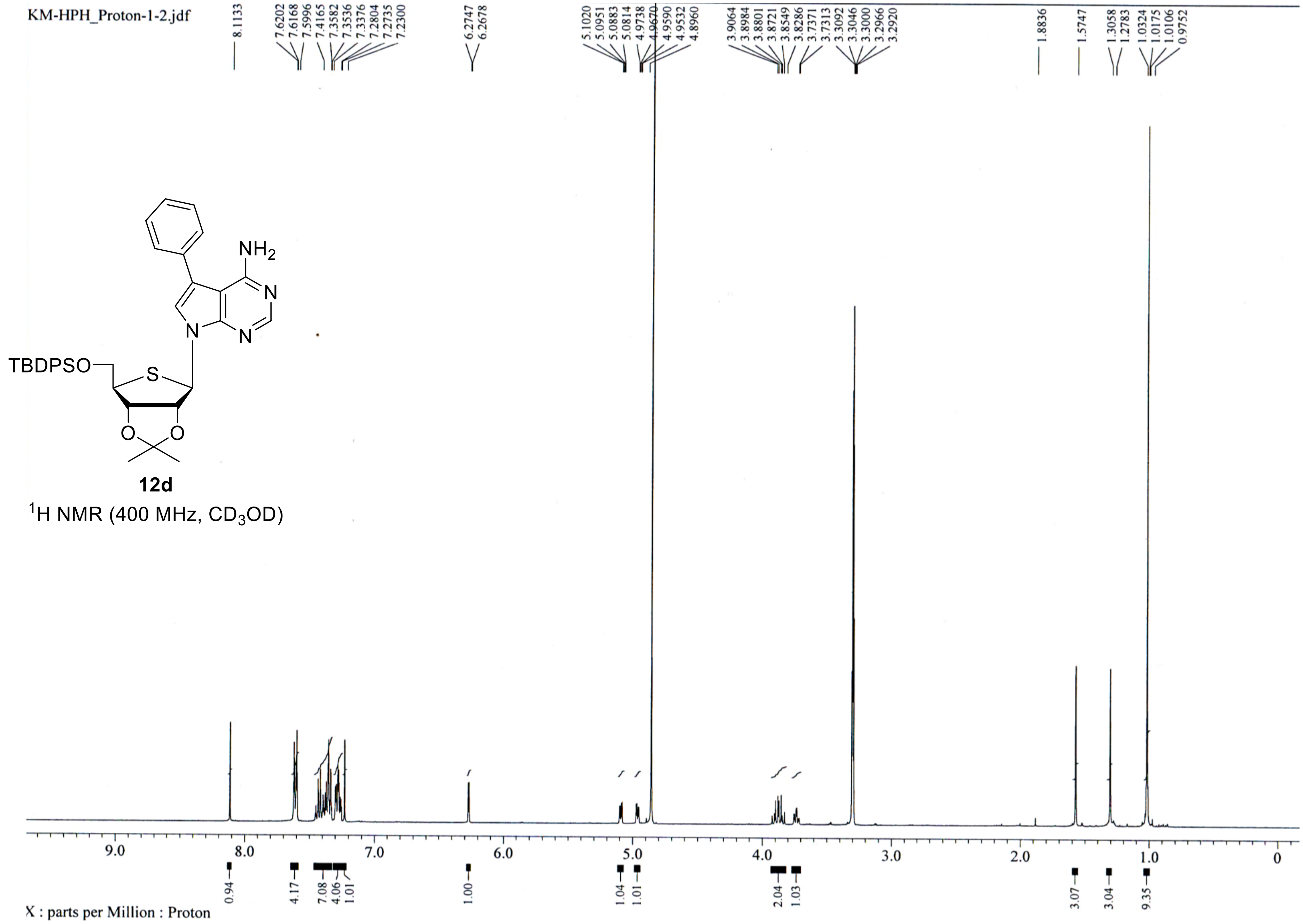

KM-HPH_Proton-1-2.jdf
8.1133
7.6202
7.6168
7.5996
7.4165
7.3582
7.3536
7.3376
7.2804
7.2735
7.2300
6.2747
6.2678
5.1020
5.0951
5.0883
5.0814
4.9738
4.9670
4.9590
4.9532
4.8960
3.9064
3.8984
3.8801
3.8721
3.8549
3.8286
3.7371
3.7313
3.3092
3.3046
3.3000
3.2966
3.2920
1.8836
1.5747
1.3058
1.2783
1.0324
1.0175
1.0106
0.9752
NH2
N
N
N
TBDPSO
S
O
O
12d
1H NMR (400 MHz, CD3OD)
9.0
8.0
7.0
6.0
5.0
4.0
3.0
2.0
1.0
0
0.94
4.17
7.08
4.06
1.01
1.00
1.04
1.01
2.04
1.03
3.07
3.04
9.35
X : parts per Million : Proton

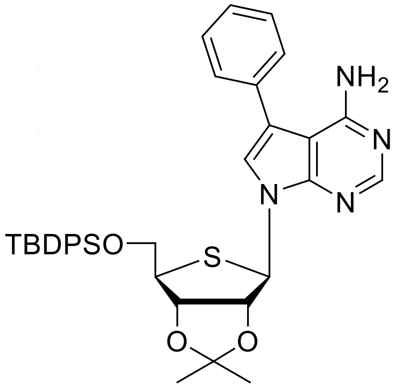

12d

^{13}C NMR (100 MHz, CD_3OD)

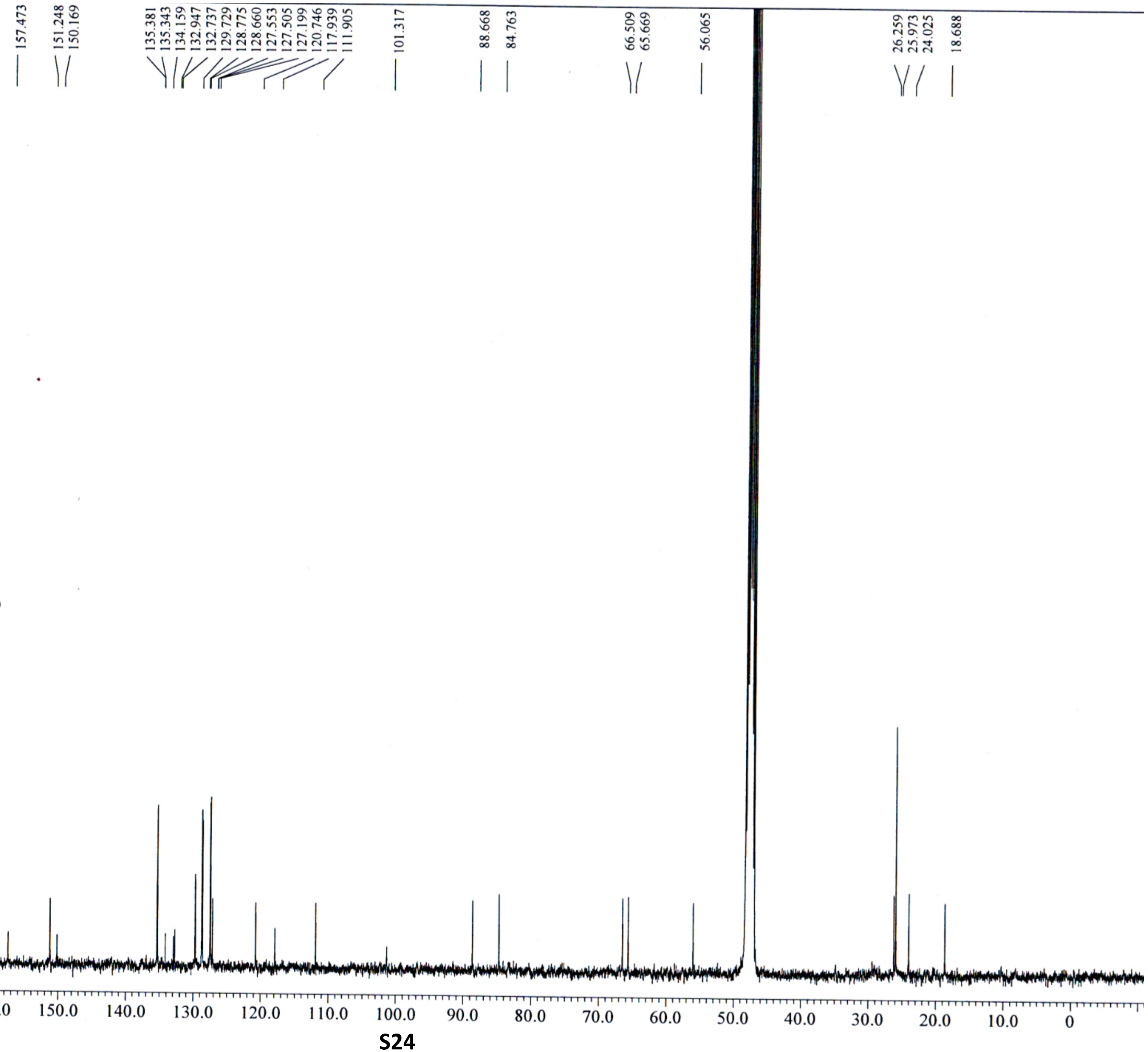

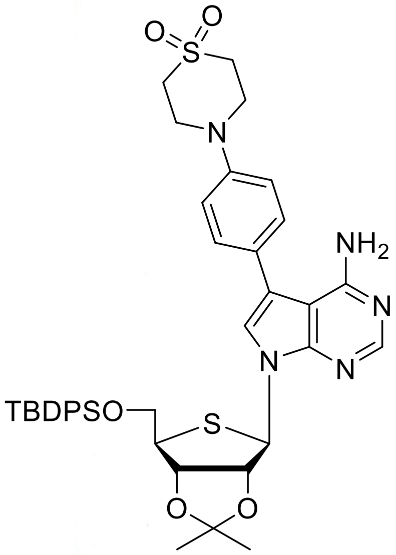

12e

^1H NMR (400 MHz, $CDCl_3$)

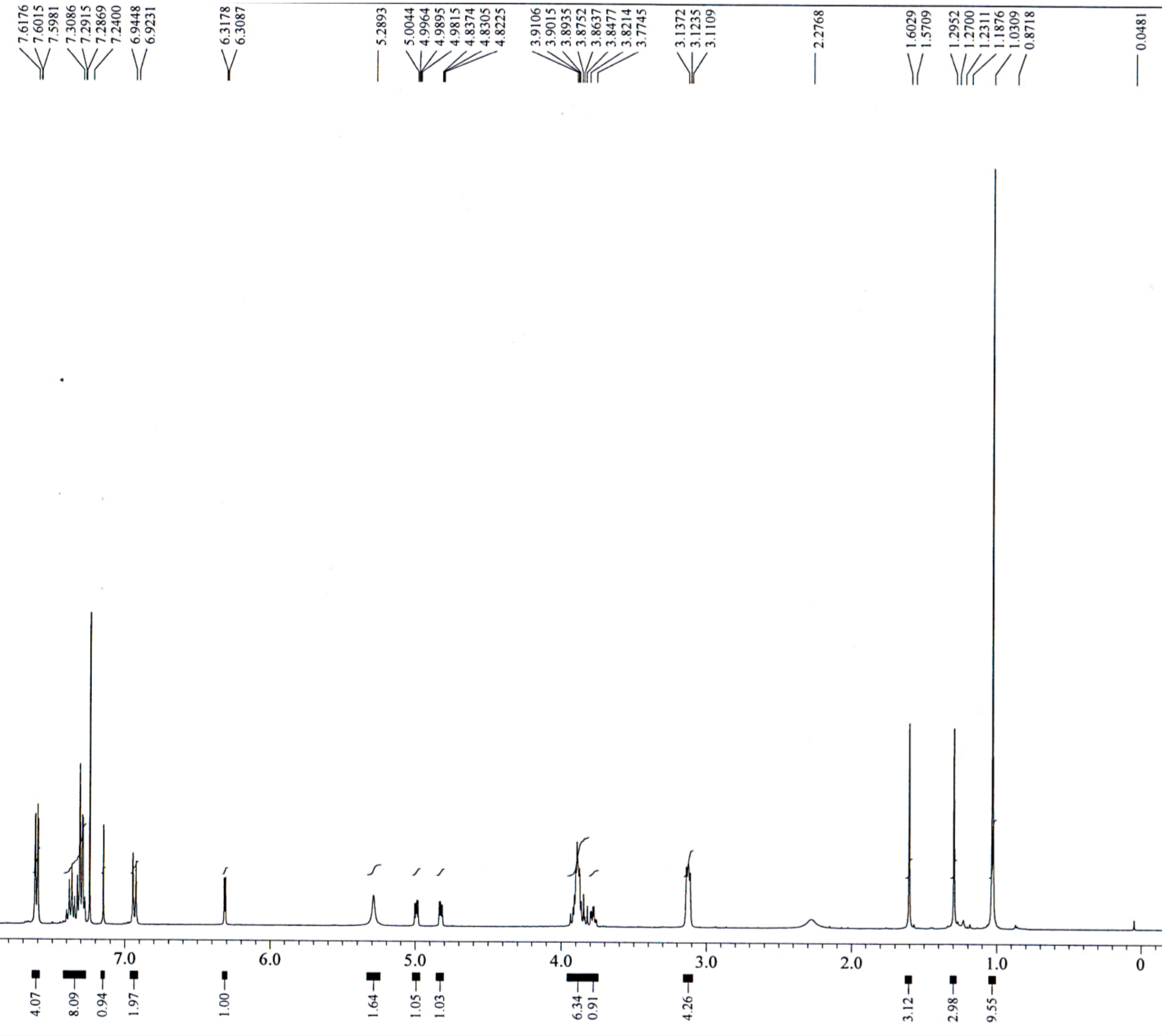

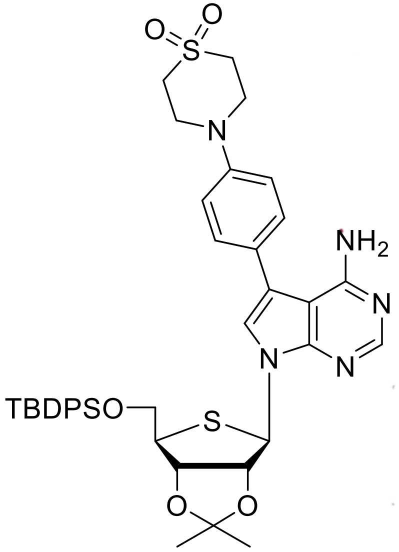

12e

^{13}C NMR (100 MHz, $CDCl_3$)

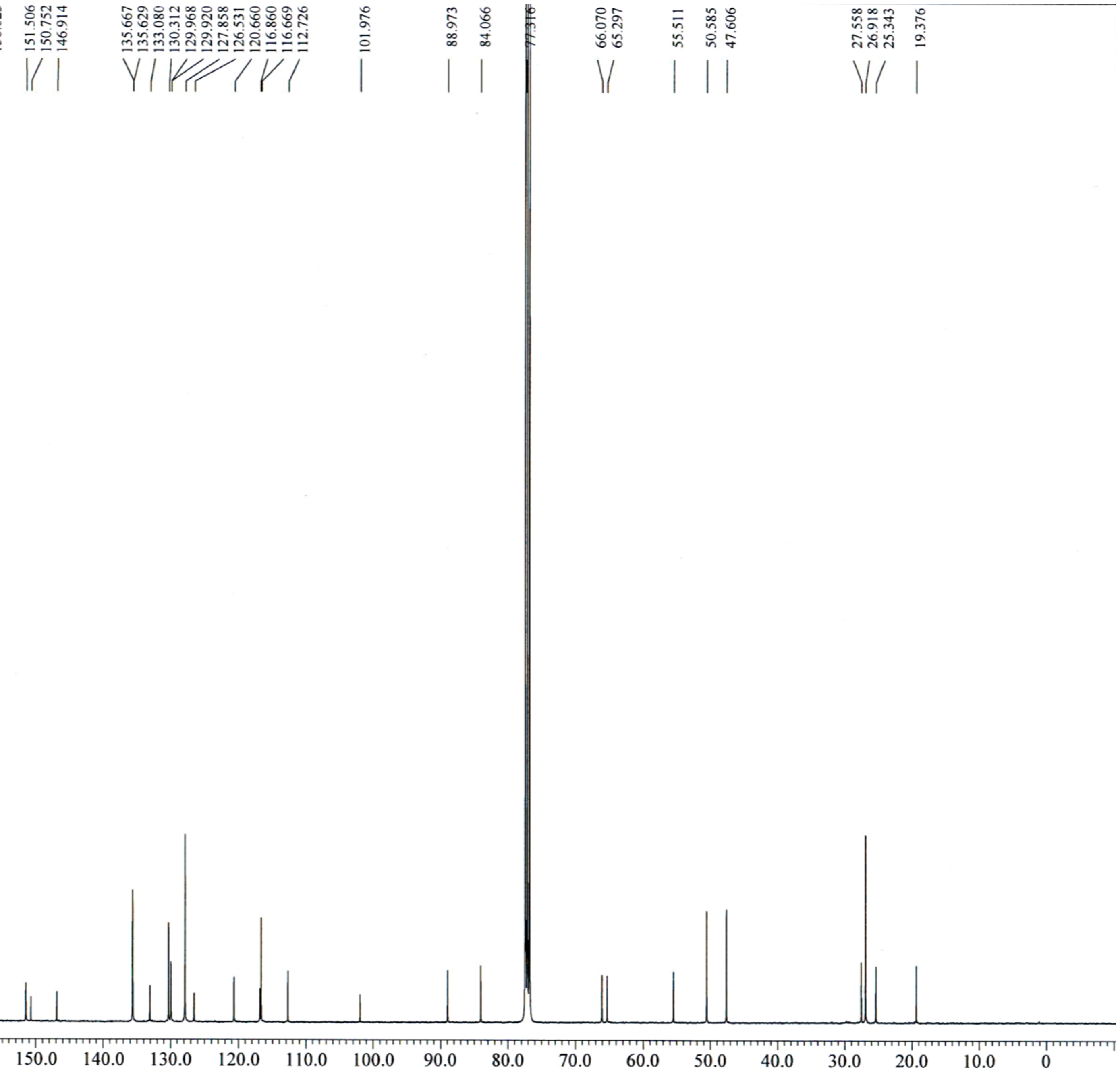

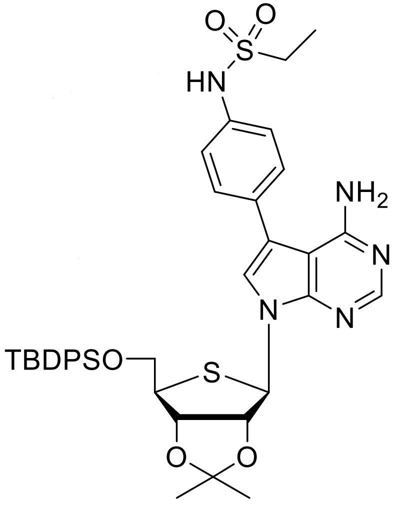

12f

^1H NMR (500 MHz, $CDCl_3$)

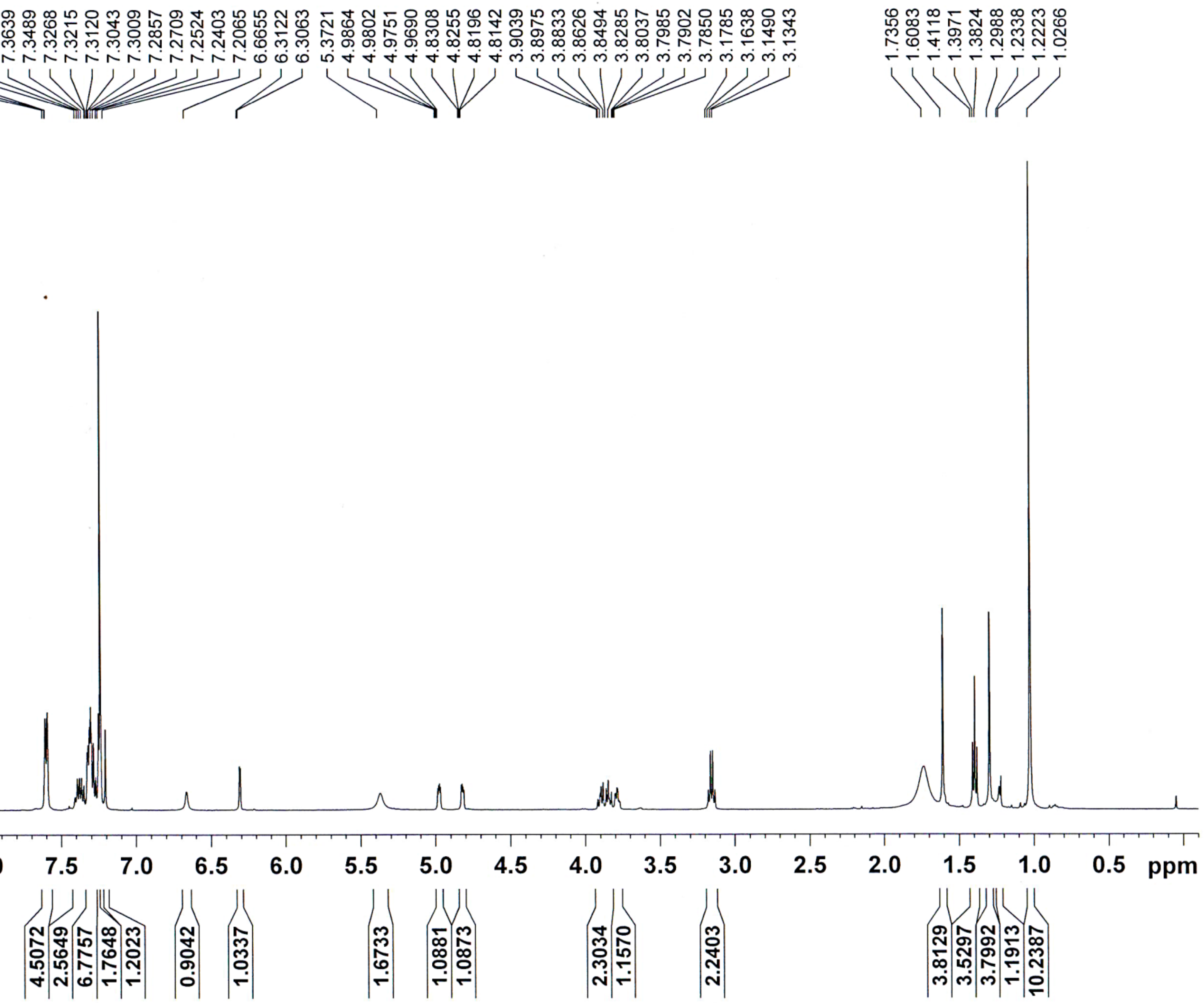

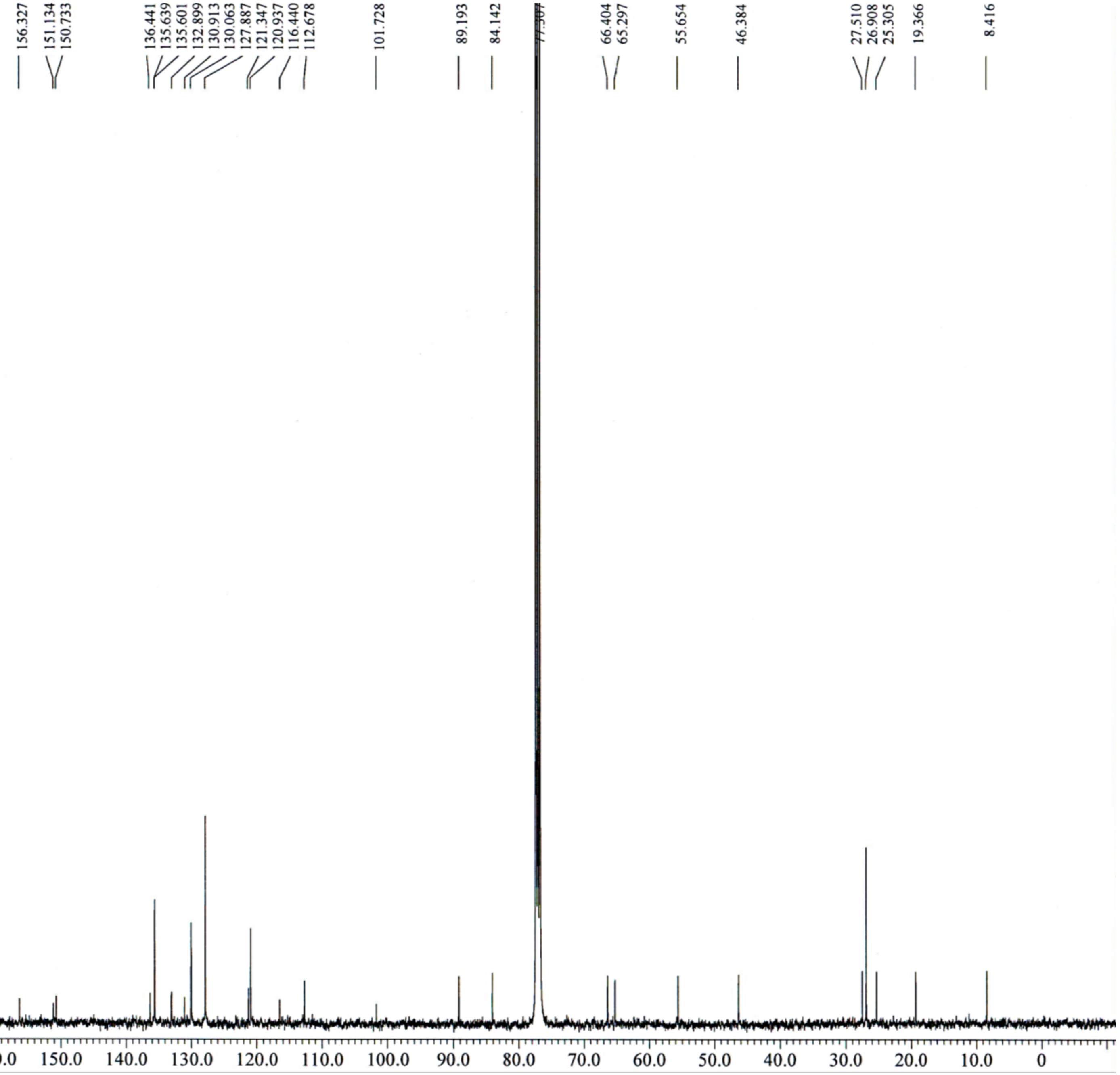

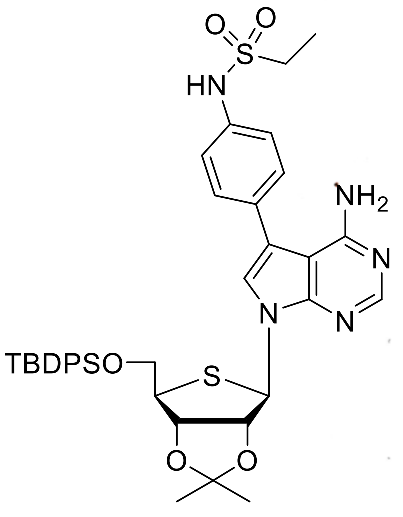

12f

^{13}C NMR (100 MHz, $CDCl_3$)

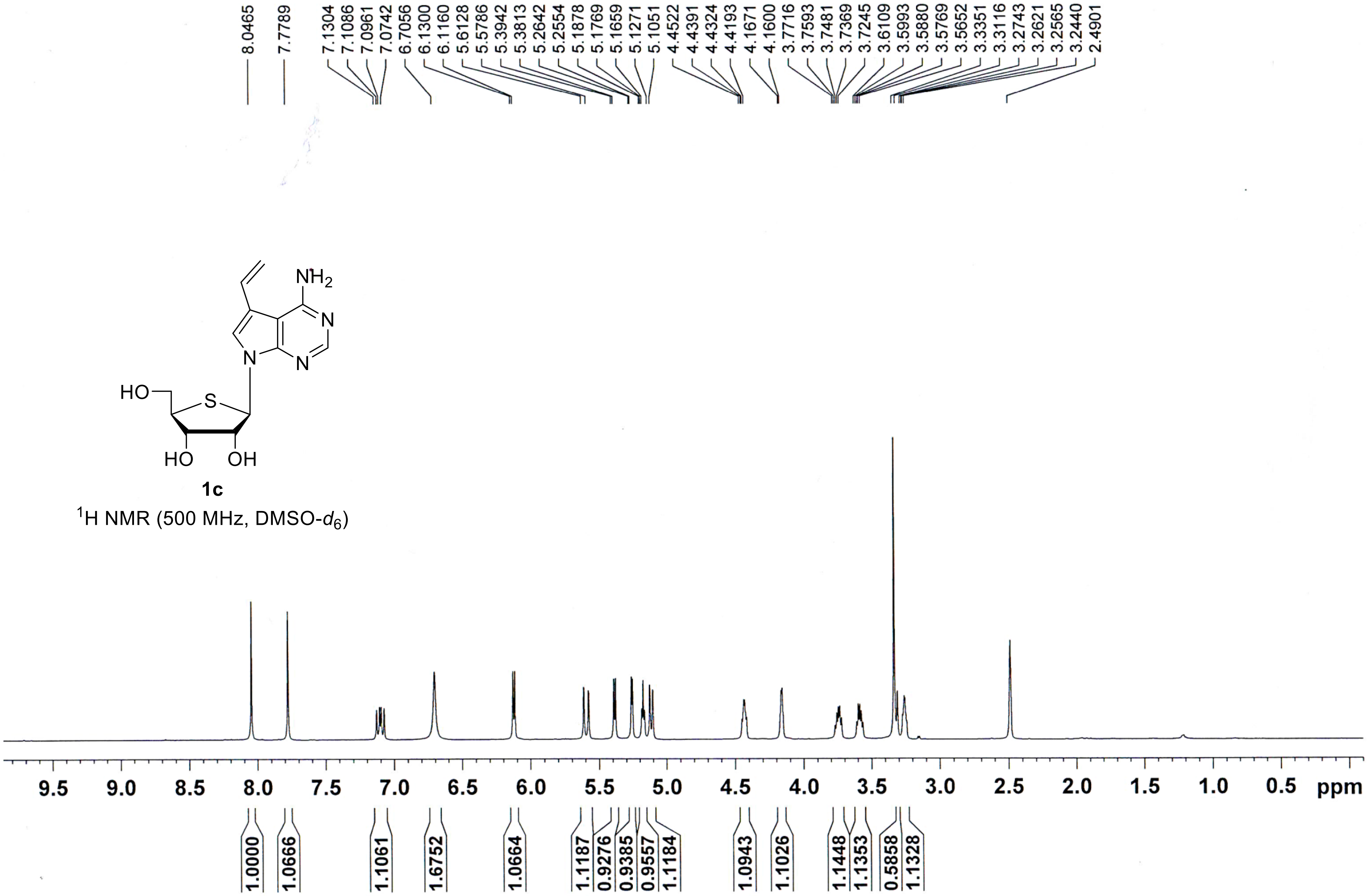
8.0465
7.7789
7.1304
7.1086
7.0961
7.0742
6.7056
6.1300
6.1160
5.6128
5.5786
5.3942
5.3813
5.2642
5.2554
5.1878
5.1769
5.1659
5.1271
5.1051
4.4522
4.4391
4.4324
4.4193
4.1671
4.1600
3.7716
3.7593
3.7481
3.7369
3.7245
3.6109
3.5993
3.5880
3.5769
3.5652
3.3351
3.3116
3.2743
3.2621
3.2565
3.2440
2.4901
NH2
N
N
N
HO
S
HO
OH
1c
1H NMR (500 MHz, DMSO-d6)
9.5
9.0
8.5
8.0
7.5
7.0
6.5
6.0
5.5
5.0
4.5
4.0
3.5
3.0
2.5
2.0
1.5
1.0
0.5
ppm
1.0000
1.0666
1.1061
1.6752
1.0664
1.1187
0.9276
0.9385
0.9557
1.1184
1.0943
1.1026
1.1448
1.1353
0.5858
1.1328

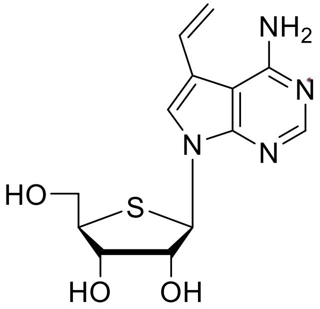

1c

^{1}H NMR (400 MHz, DMSO-d_6 + D_2O)

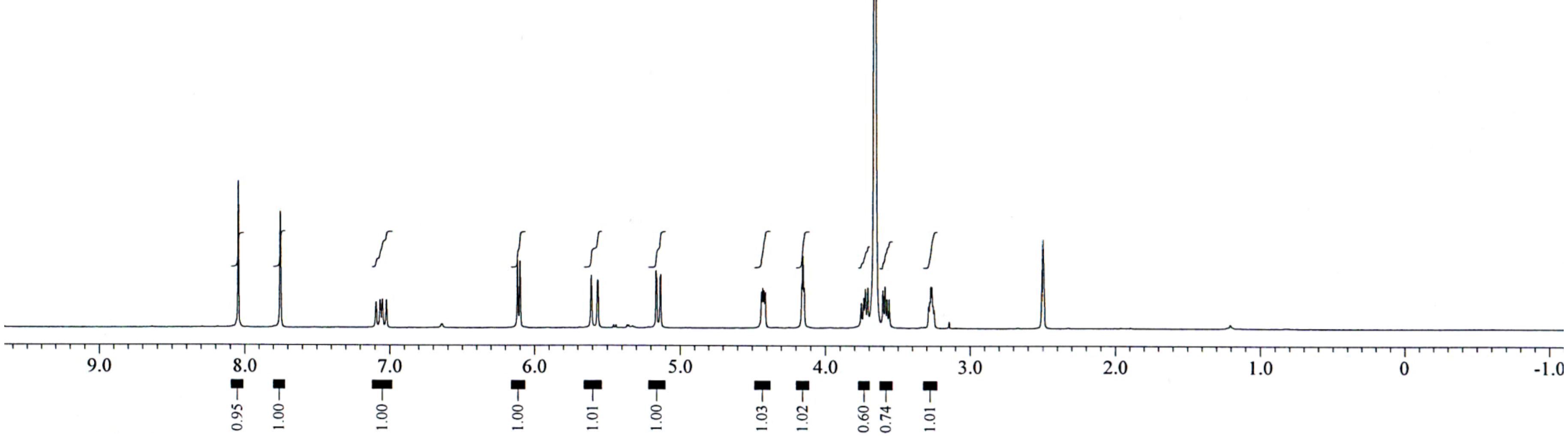

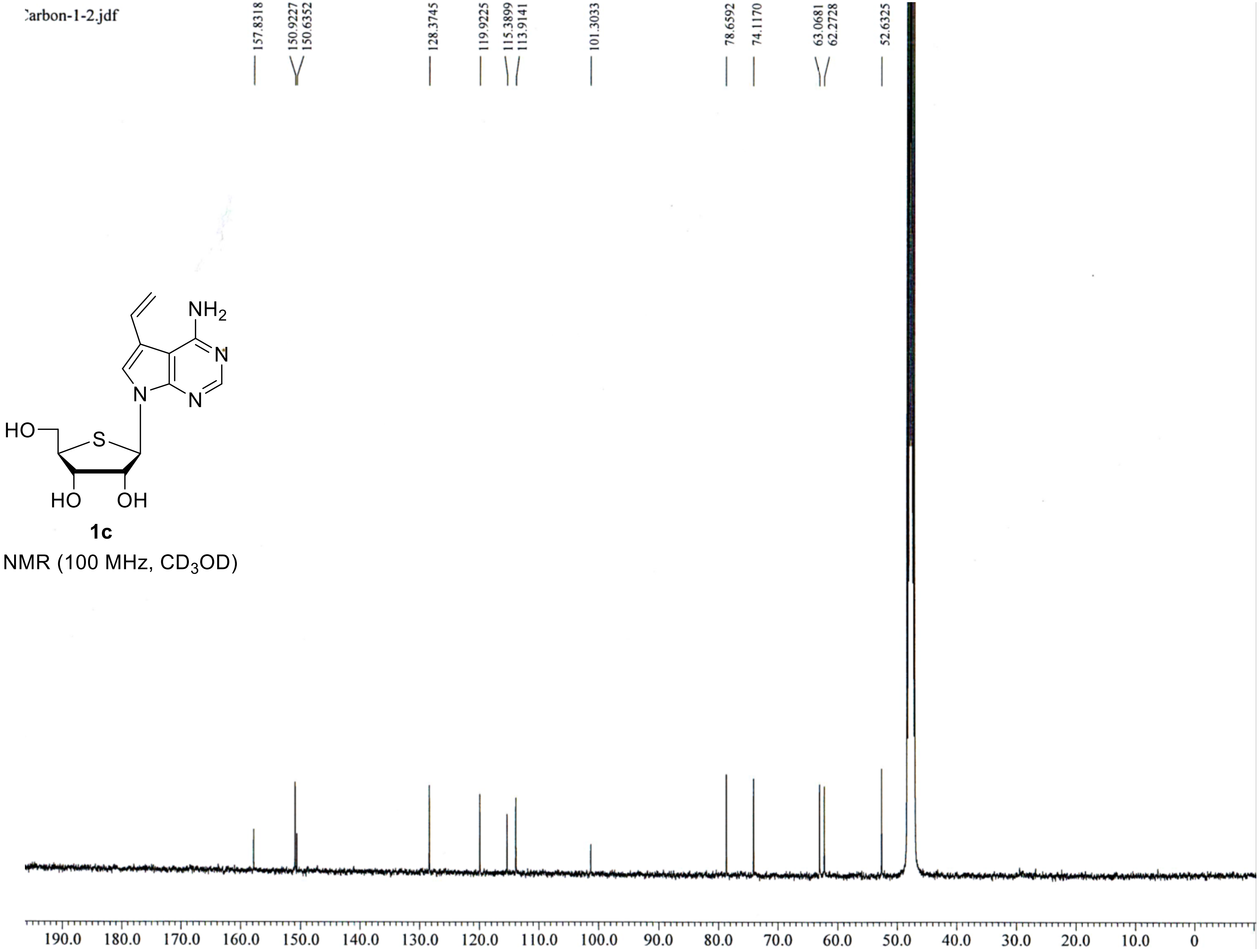

1c

^{13}C NMR (100 MHz, CD_3OD)

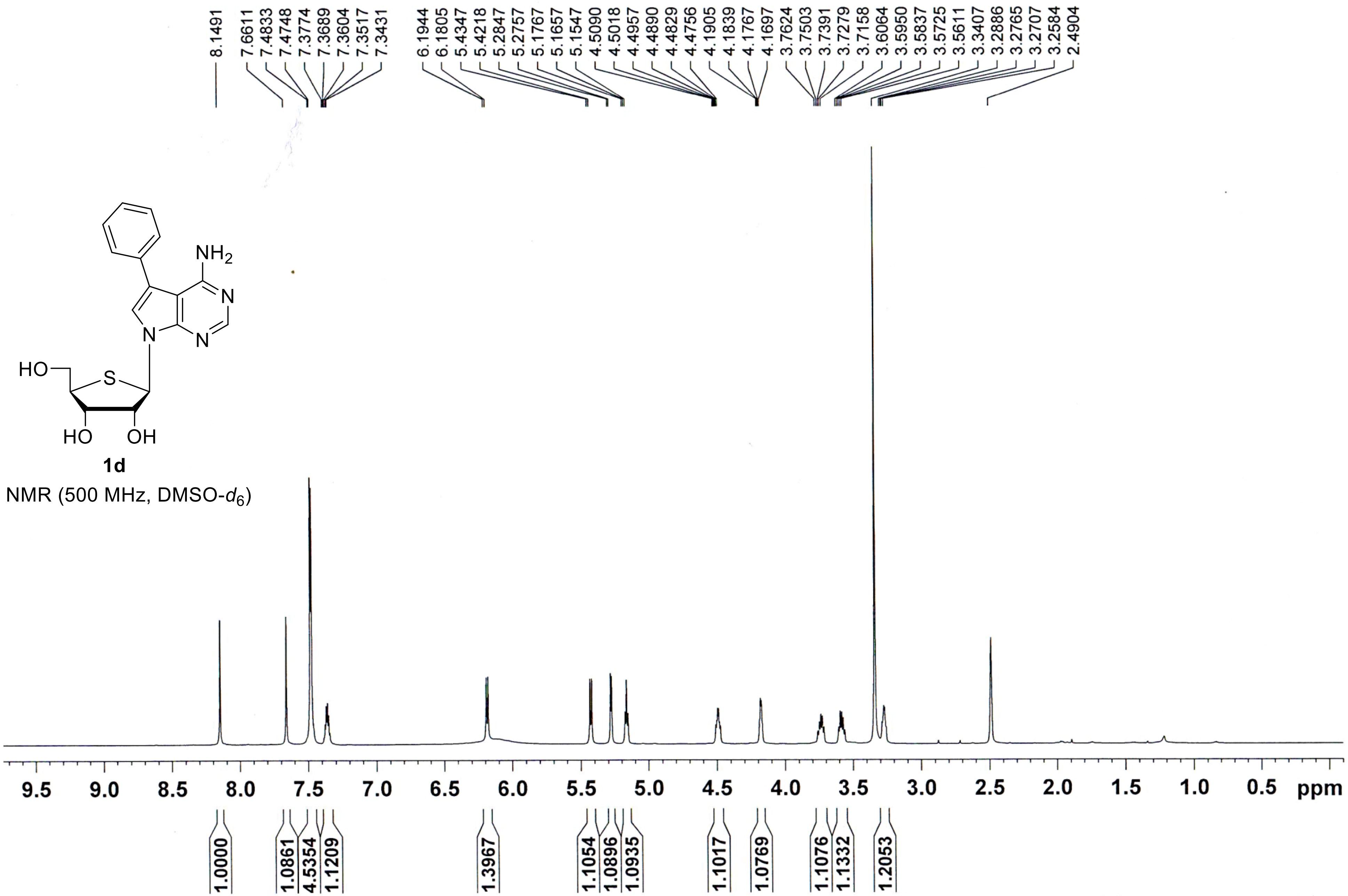

1d

^{1}H NMR (500 MHz, DMSO-d_6)

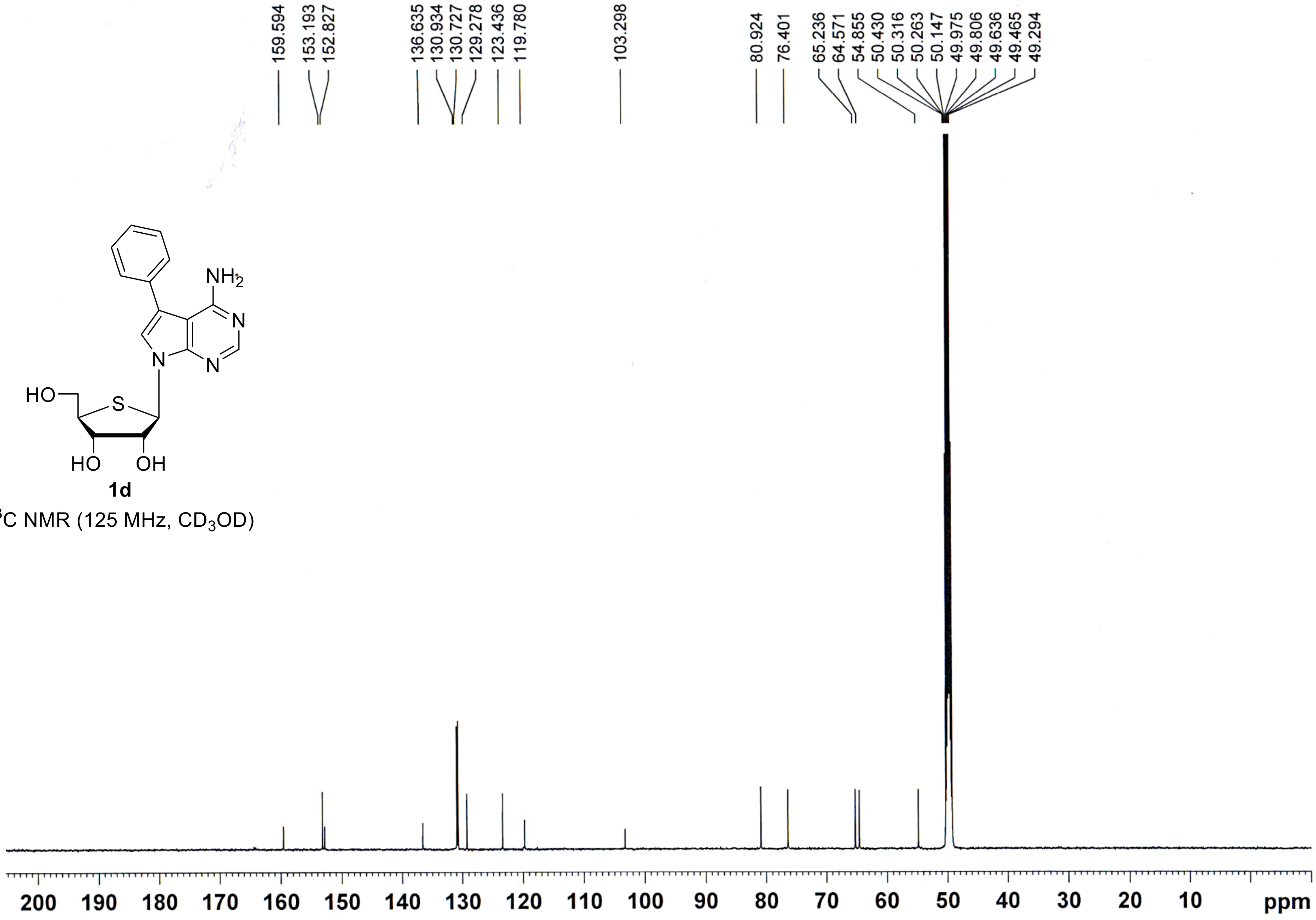

1d

^{13}C NMR (125 MHz, CD_3OD)

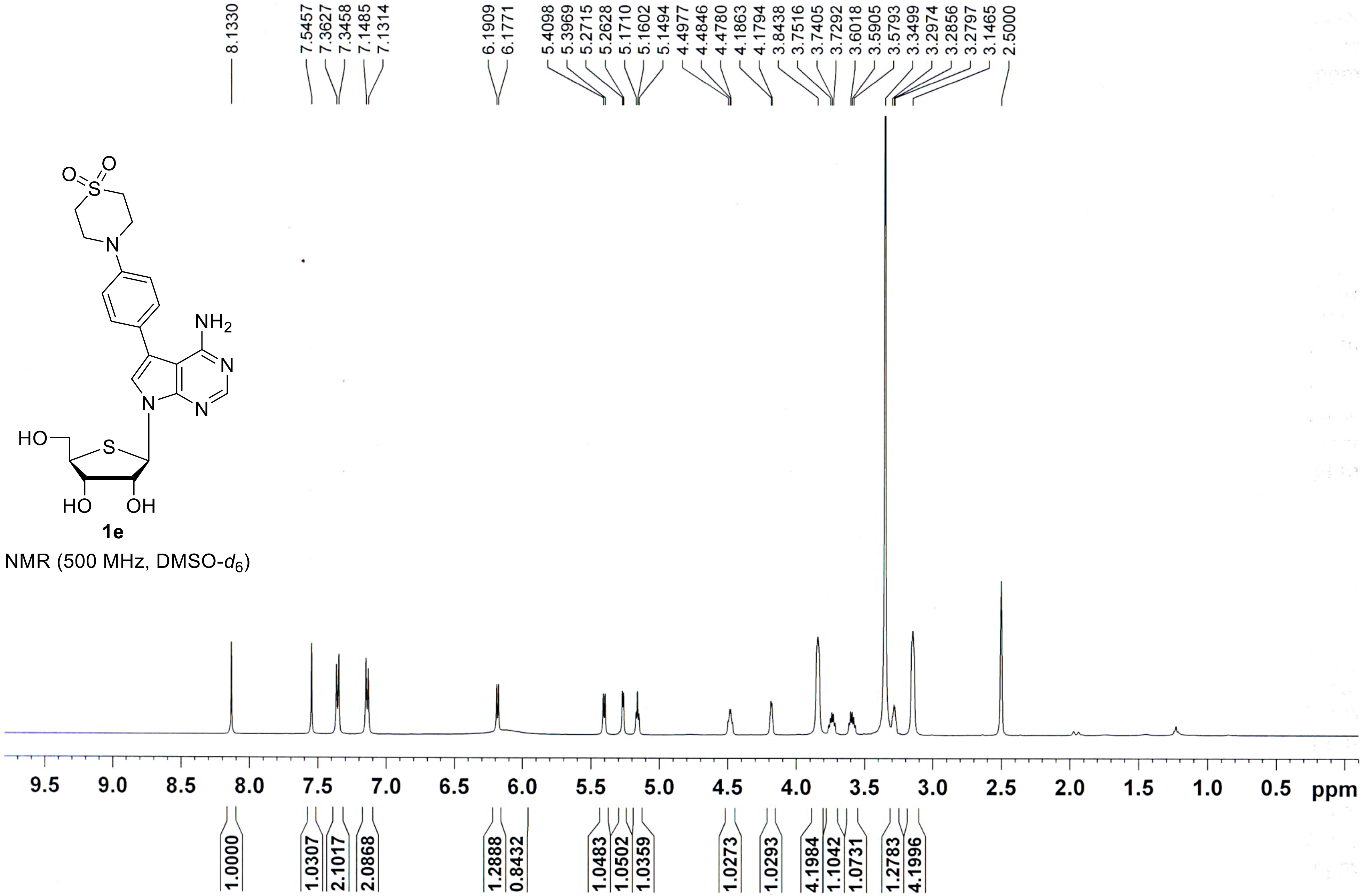

1e

^{1}H NMR (500 MHz, DMSO-d_6)

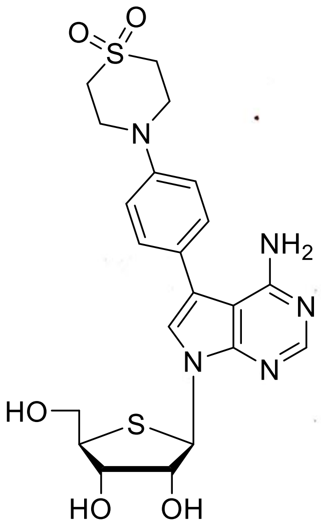

1e

^{13}C NMR (125 MHz, DMSO-d_6)

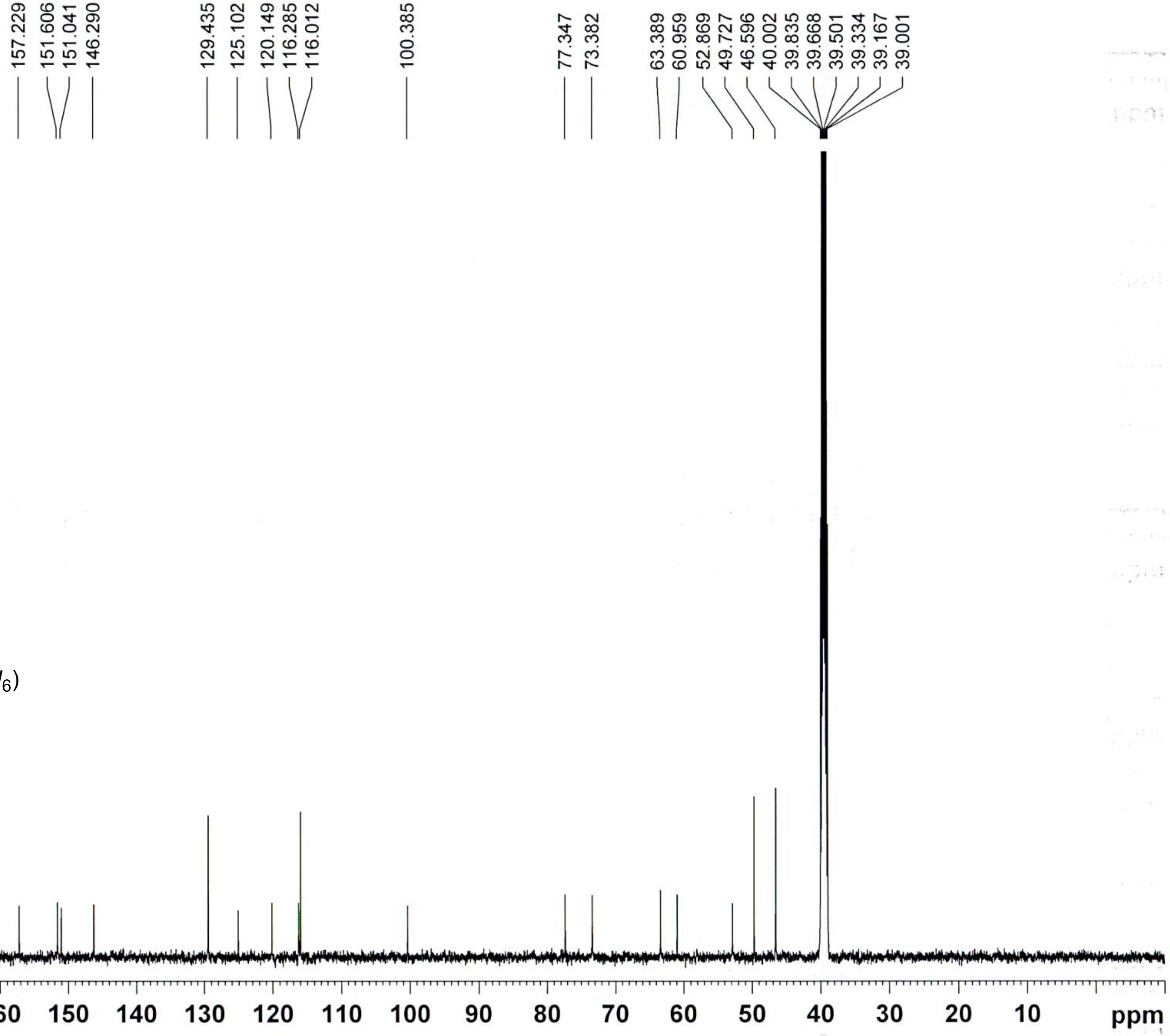

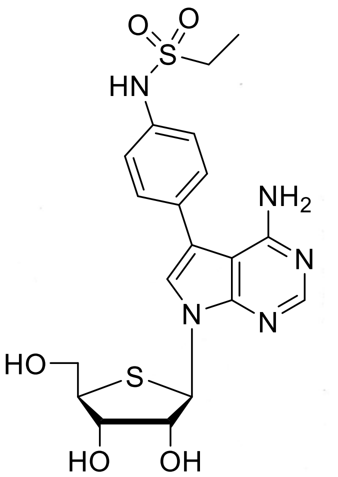

1f

^{1}H NMR (500 MHz, DMSO-d_6)

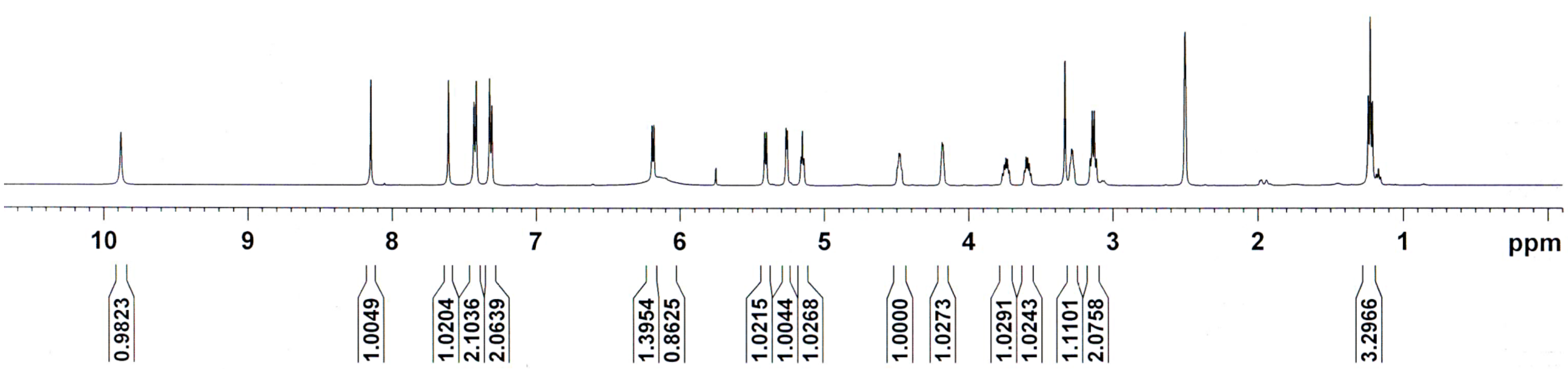

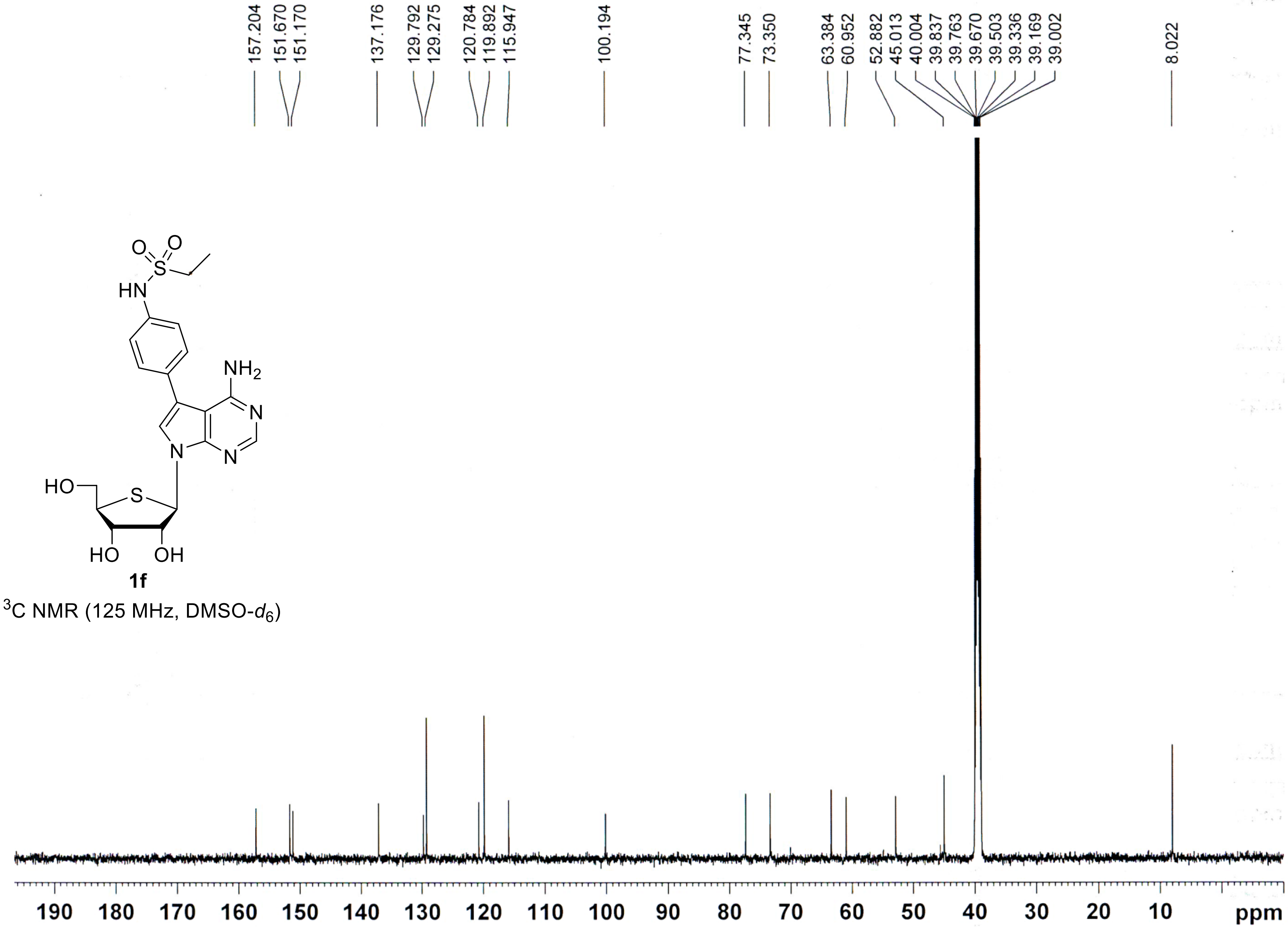

1f

^{13}C NMR (125 MHz, DMSO-d_6)

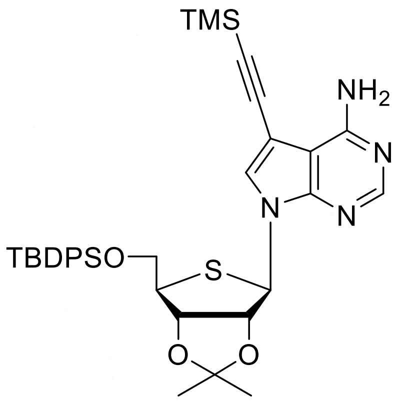

13

^{1}H NMR (500 MHz, $CDCl_3$)

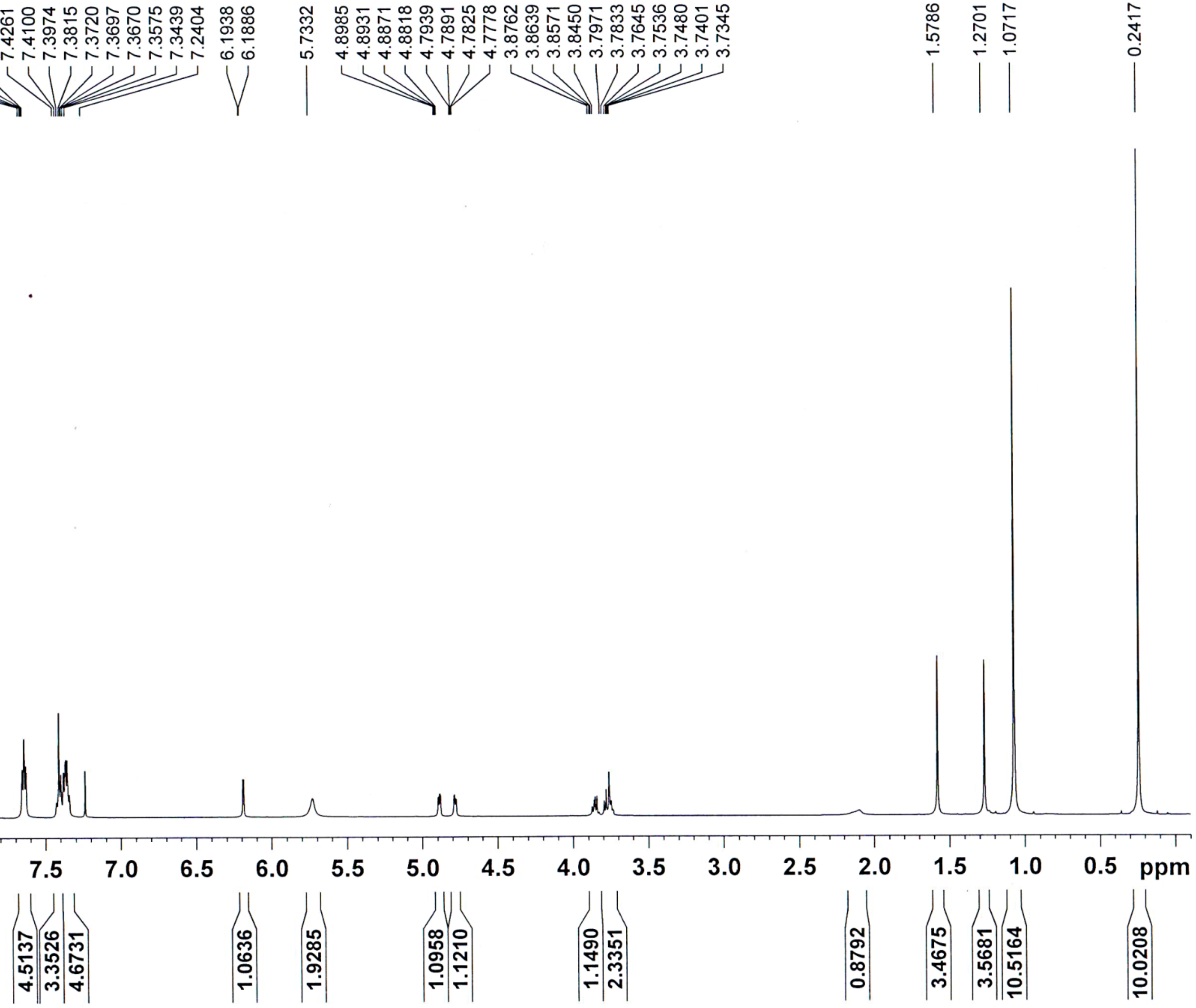

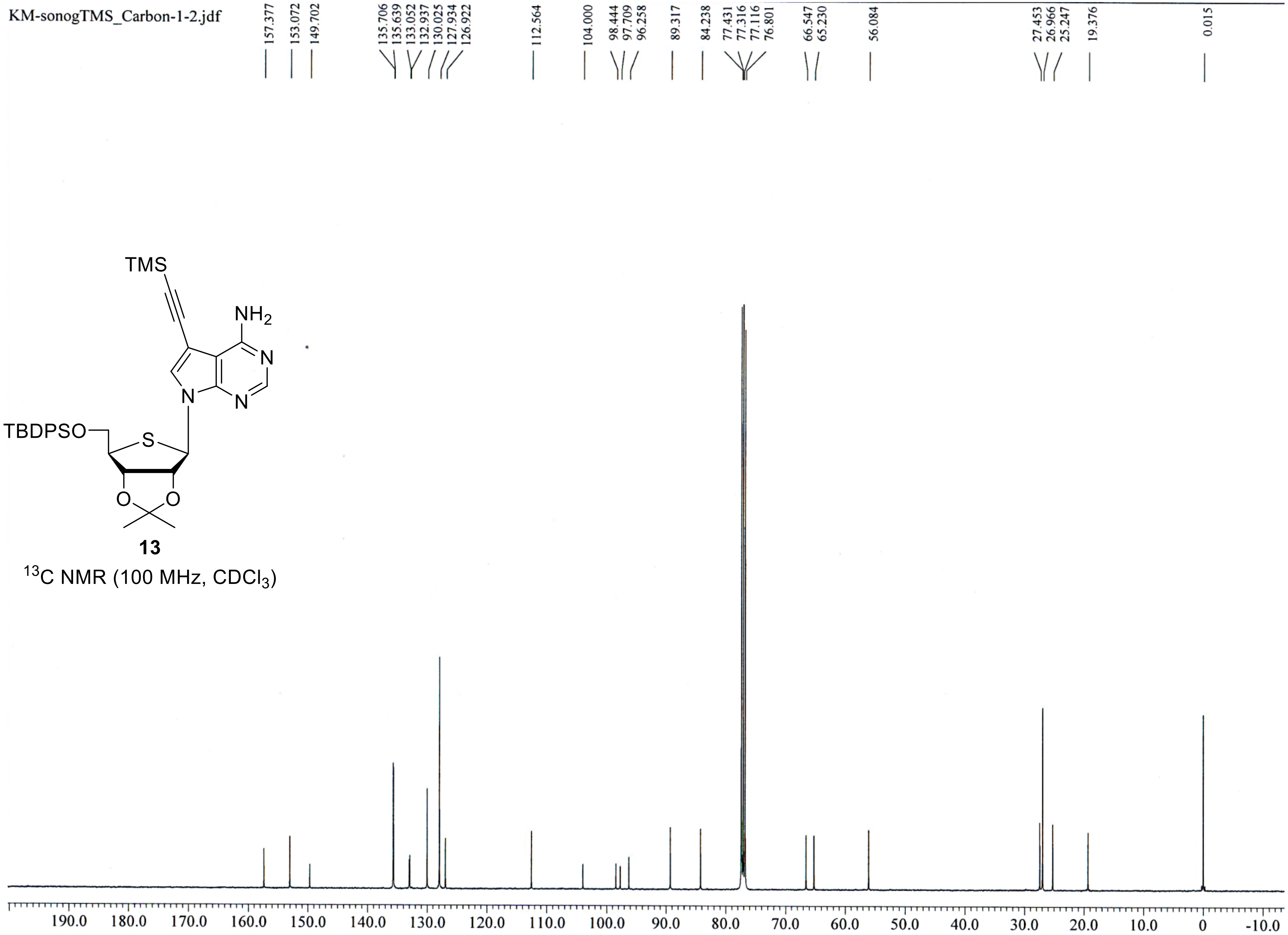

13

^{13}C NMR (100 MHz, $CDCl_3$)

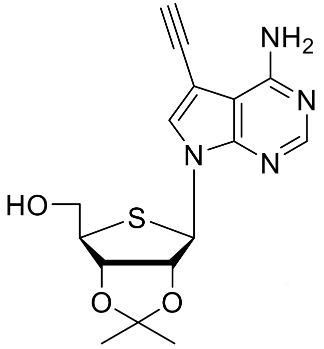

14

^1H NMR (500 MHz, CD_3OD)

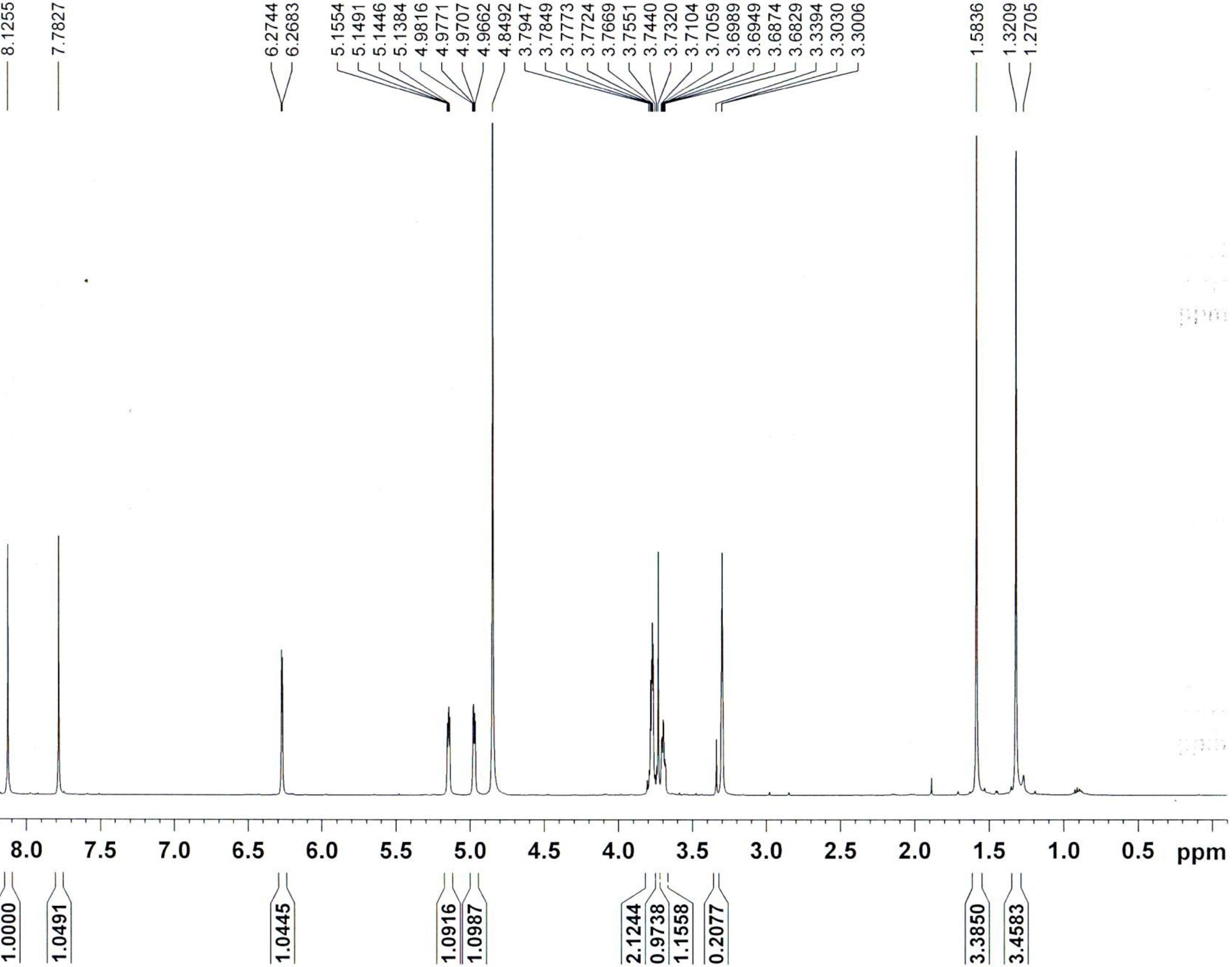

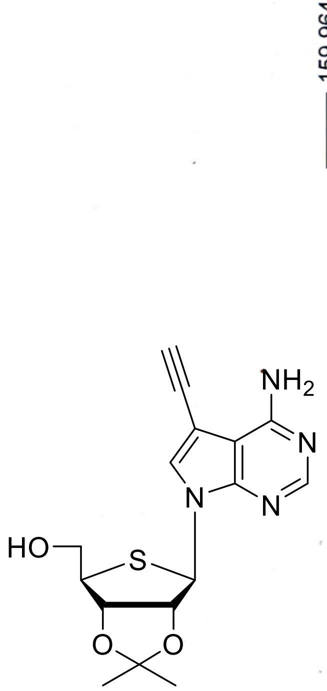

14

^{13}C NMR (125 MHz, CD_3OD)

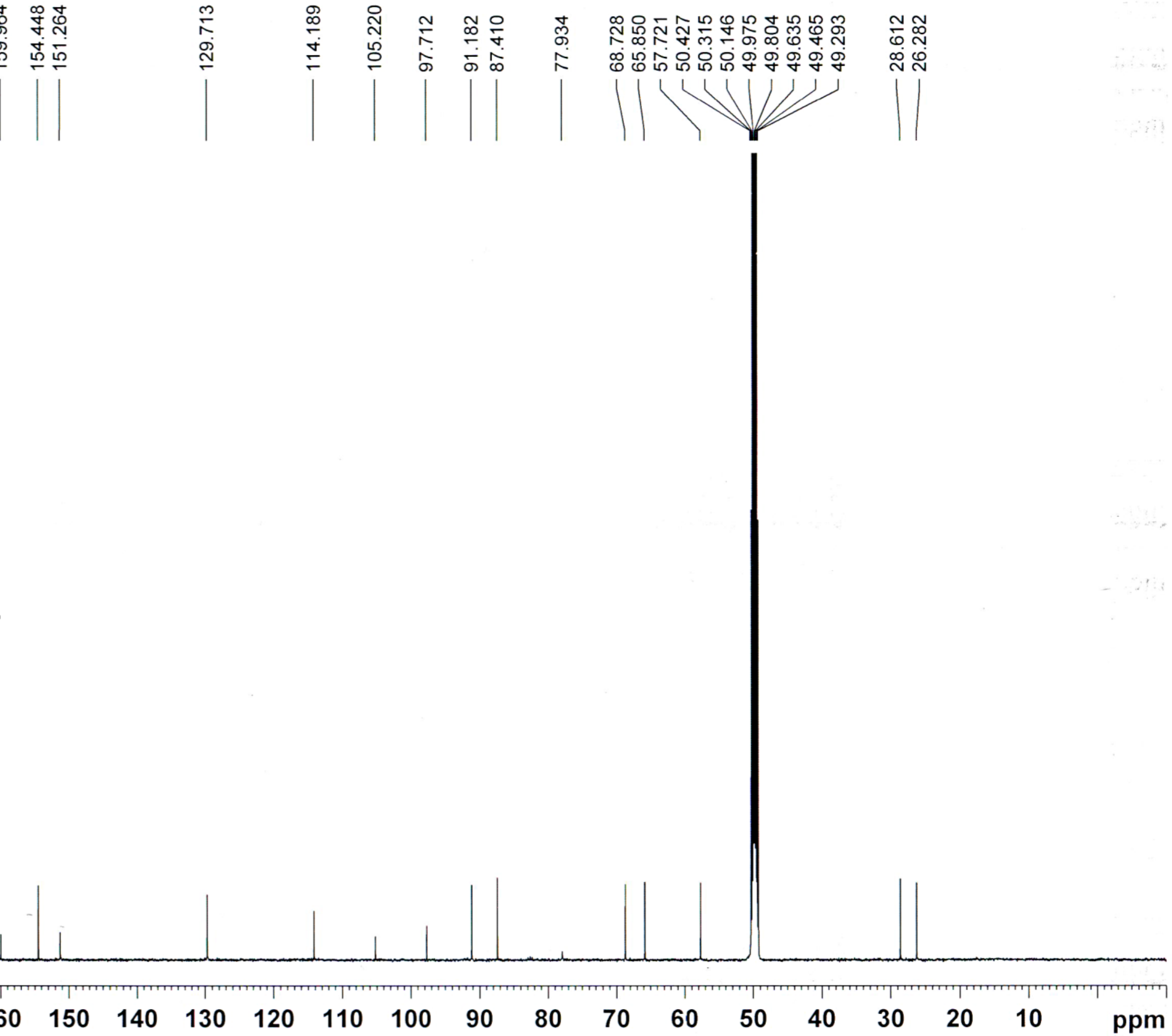

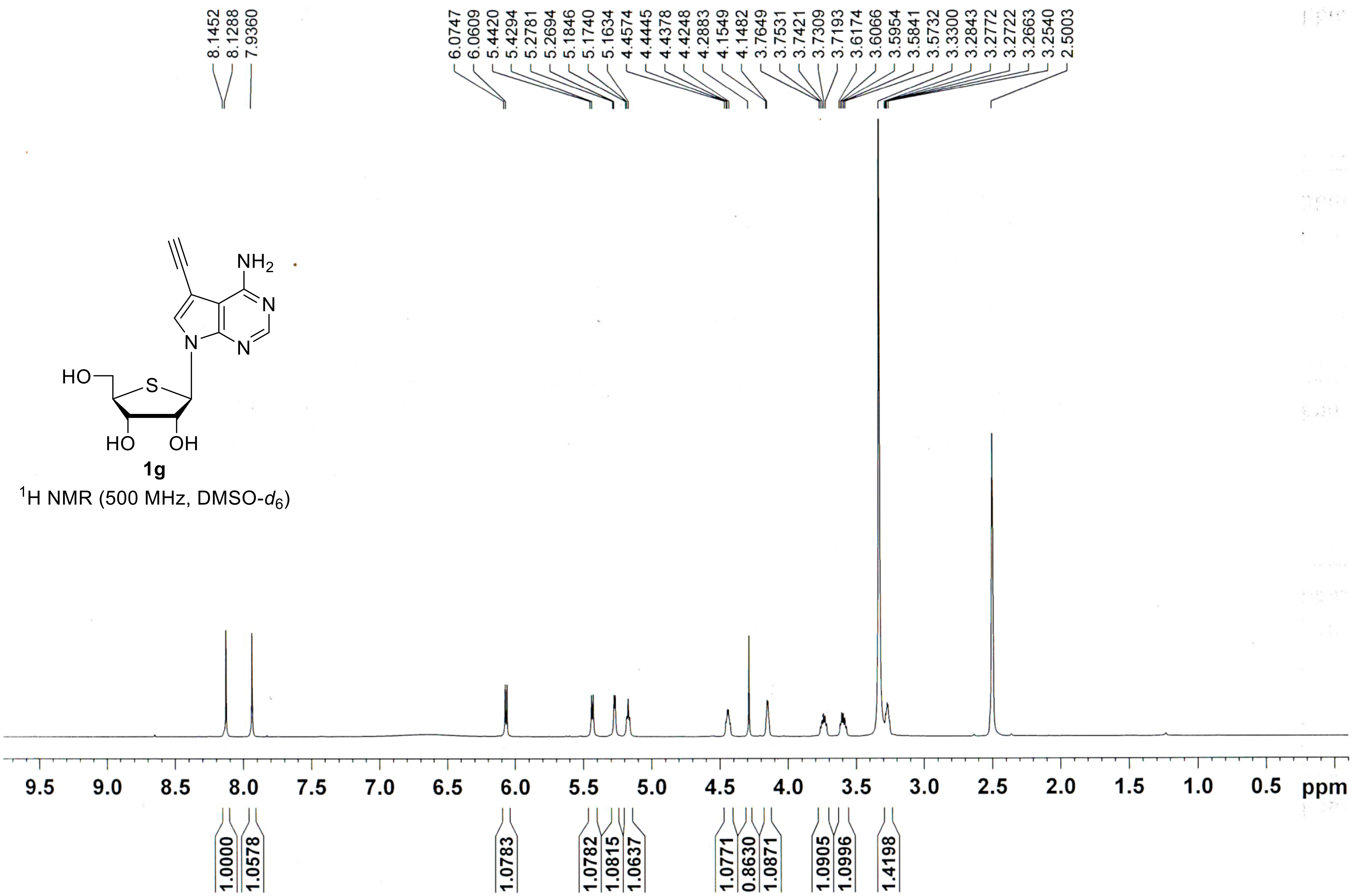

1g

^{1}H NMR (500 MHz, DMSO-d_6)

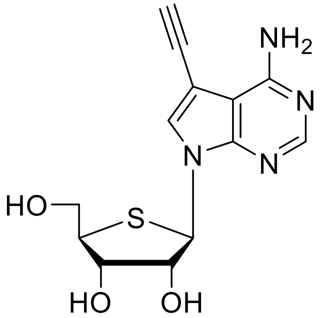

1g

^{13}C NMR (150 MHz, DMSO-d_6)

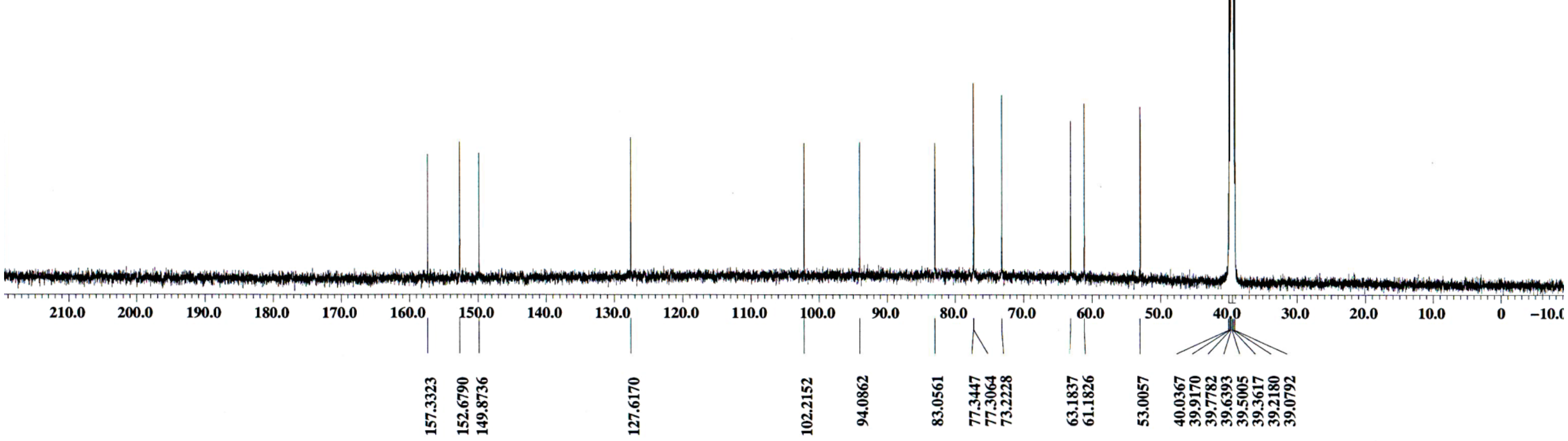

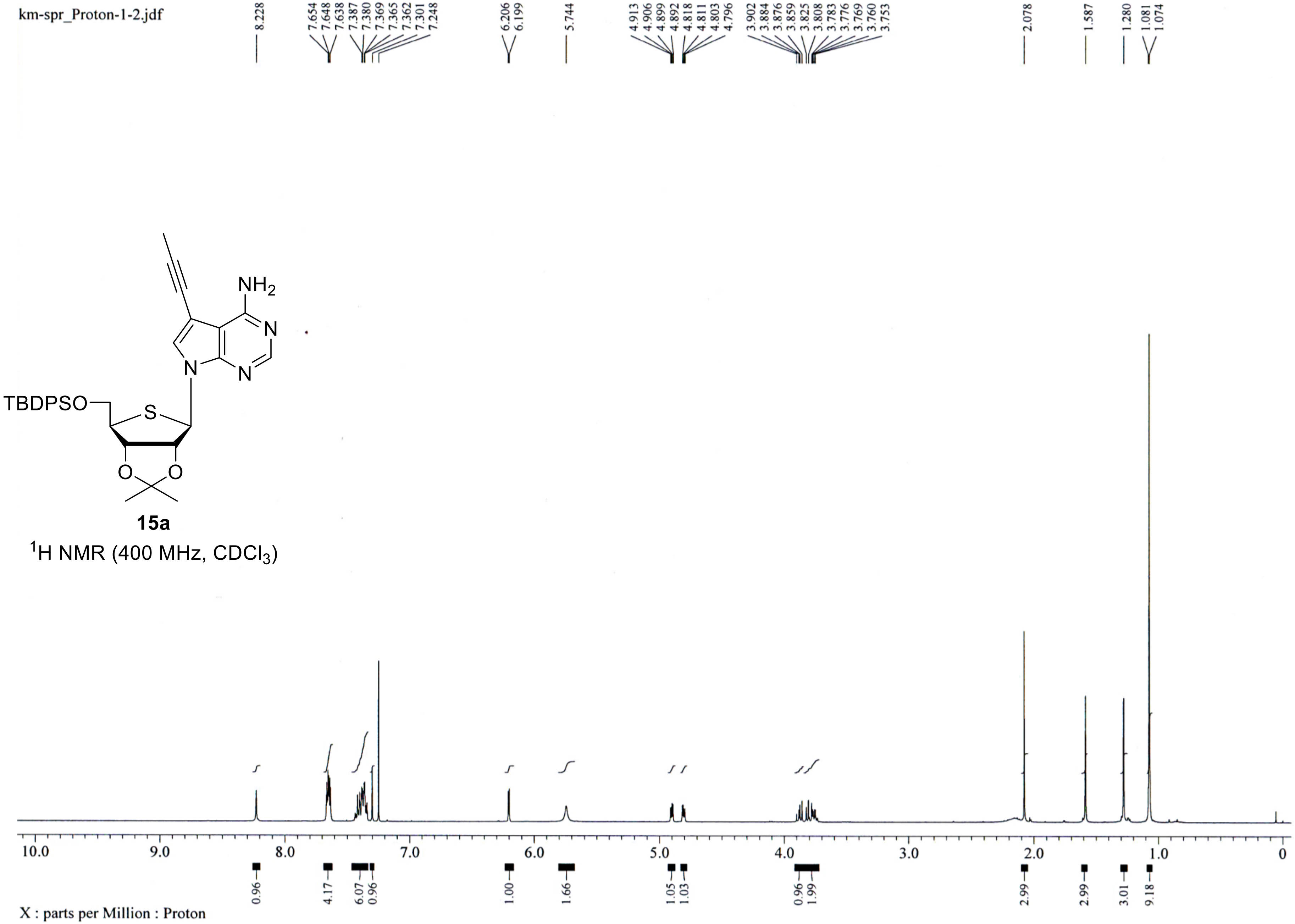

15a

^1H NMR (400 MHz, $CDCl_3$)

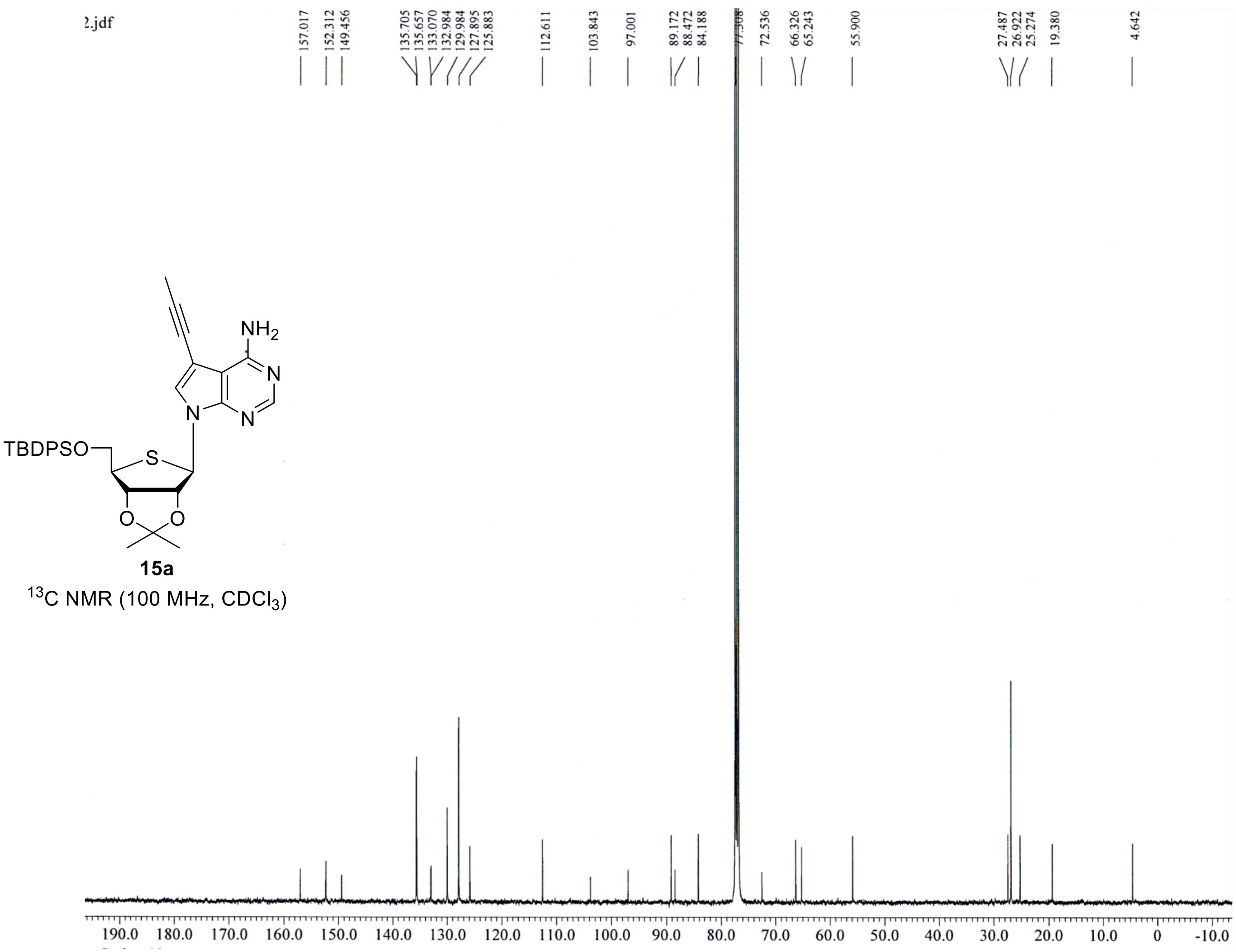

15a

^{13}C NMR (100 MHz, $CDCl_3$)

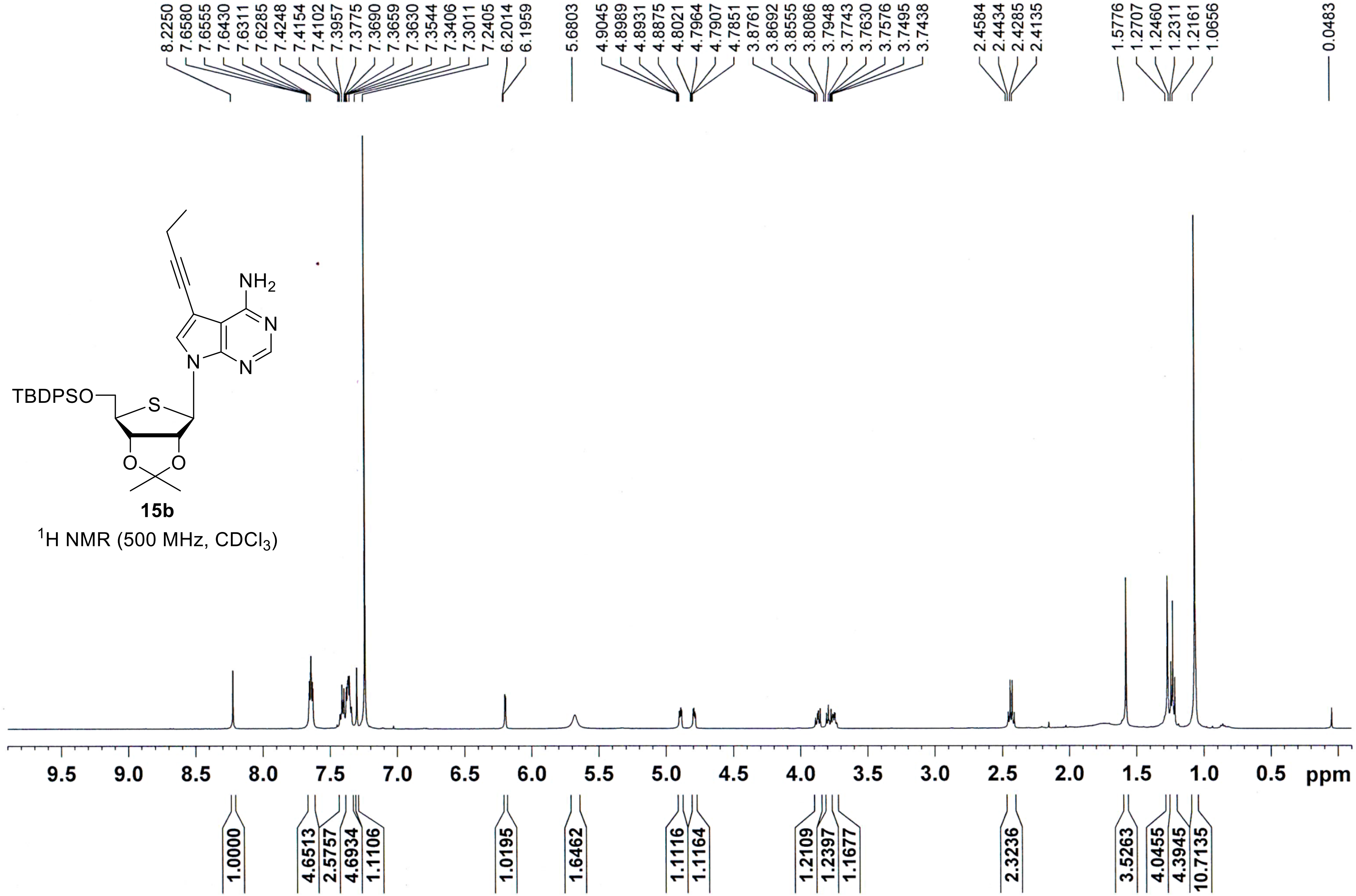

15b

^{1}H NMR (500 MHz, $CDCl_3$)

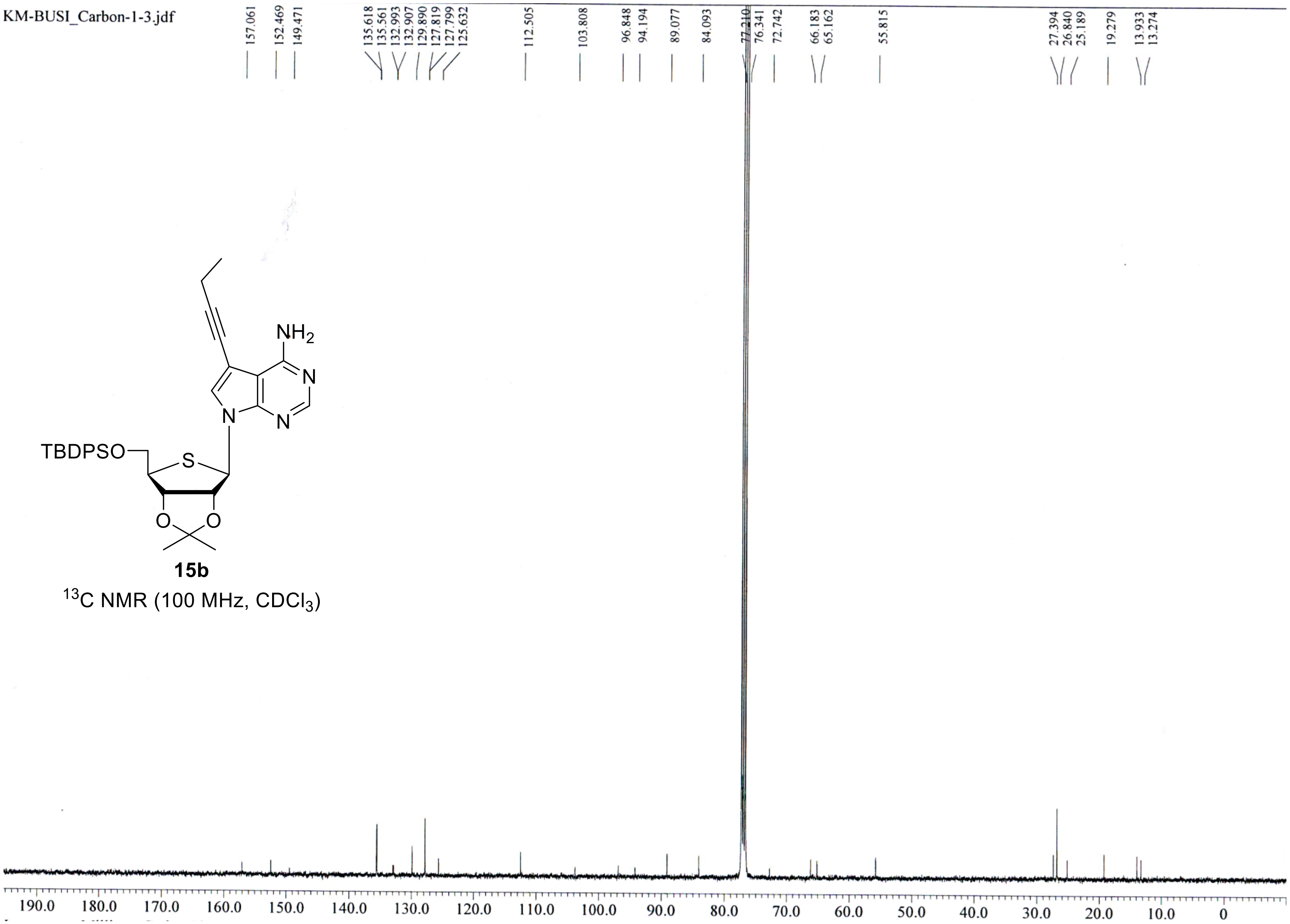

15b

^{13}C NMR (100 MHz, $CDCl_3$)

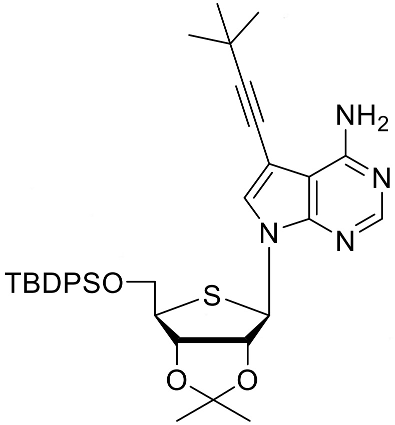

15c

^{1}H NMR (500 MHz, $CDCl_3$)

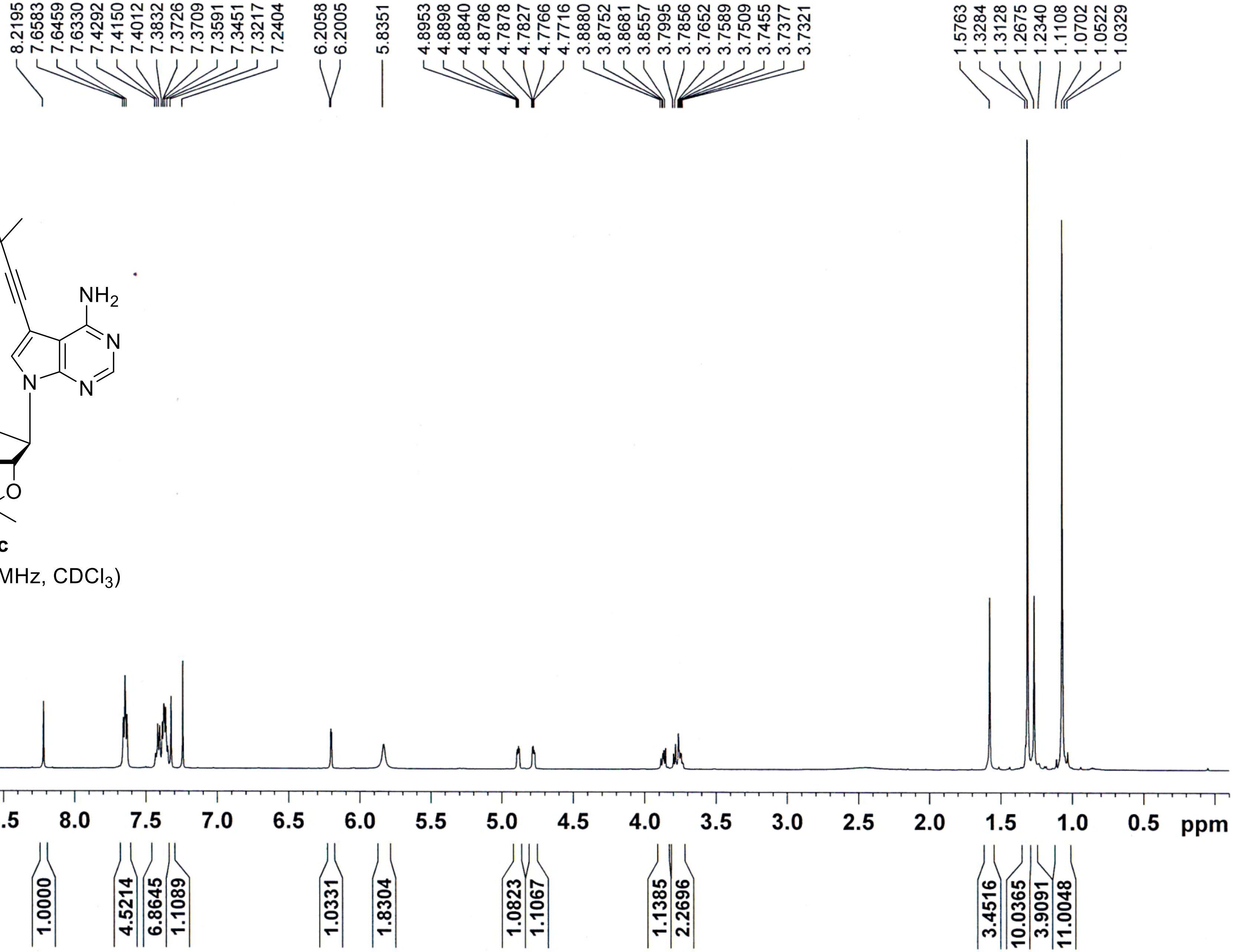

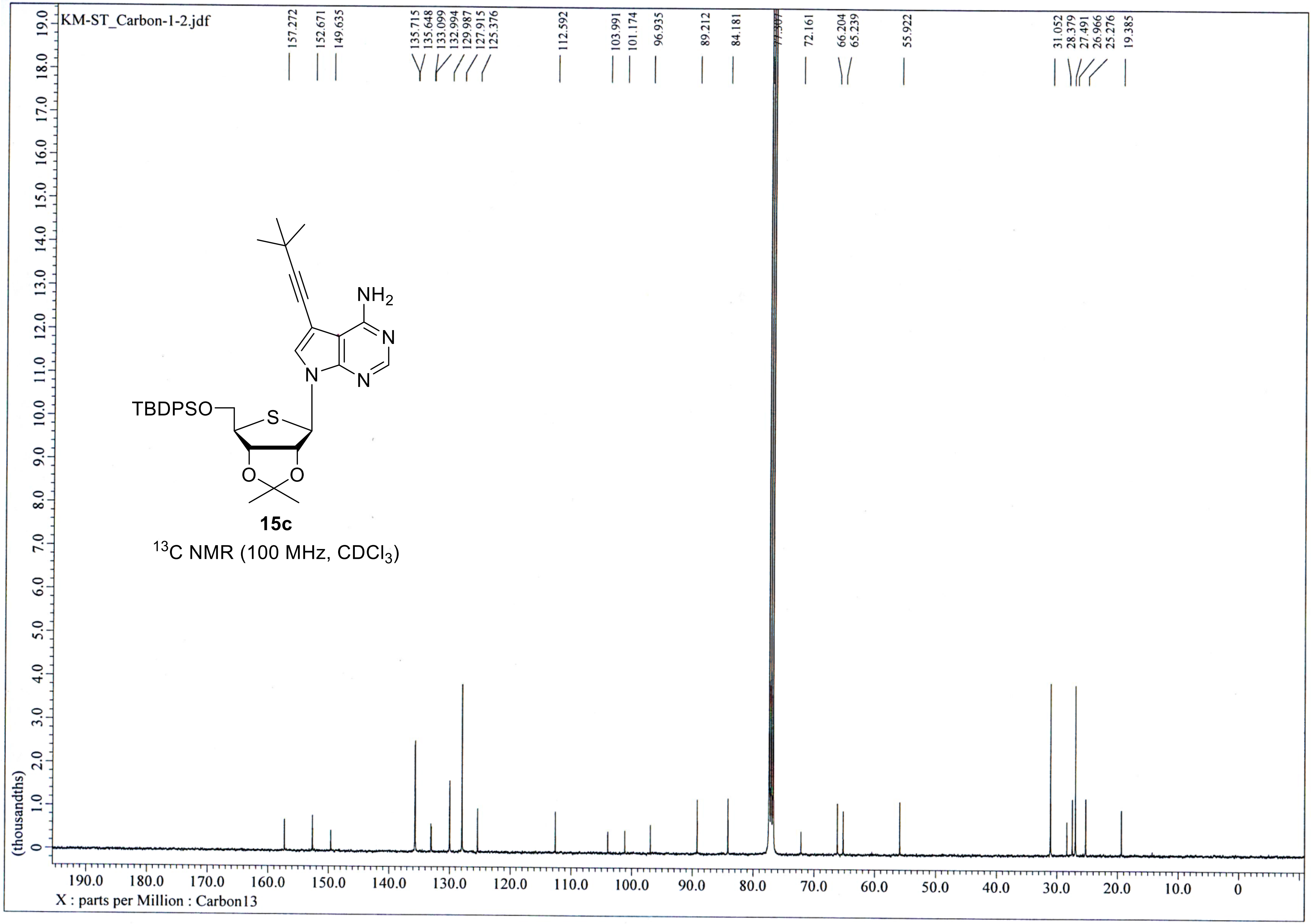
KM-ST_Carbon-1-2.jdf
157.272
152.671
149.635
135.715
135.648
133.099
132.994
129.987
127.915
125.376
112.592
103.991
101.174
96.935
89.212
84.181
72.161
66.204
65.239
55.922
31.052
28.379
27.491
26.966
25.276
19.385
NH2
N
N
N
S
TBDPSO
O
O
15c
13C NMR (100 MHz, CDCl3)
(thousandths)
X : parts per Million : Carbon13

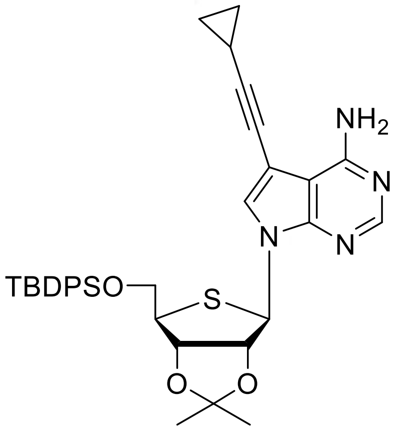

15d

^{1}H NMR (400 MHz, $CDCl_3$)

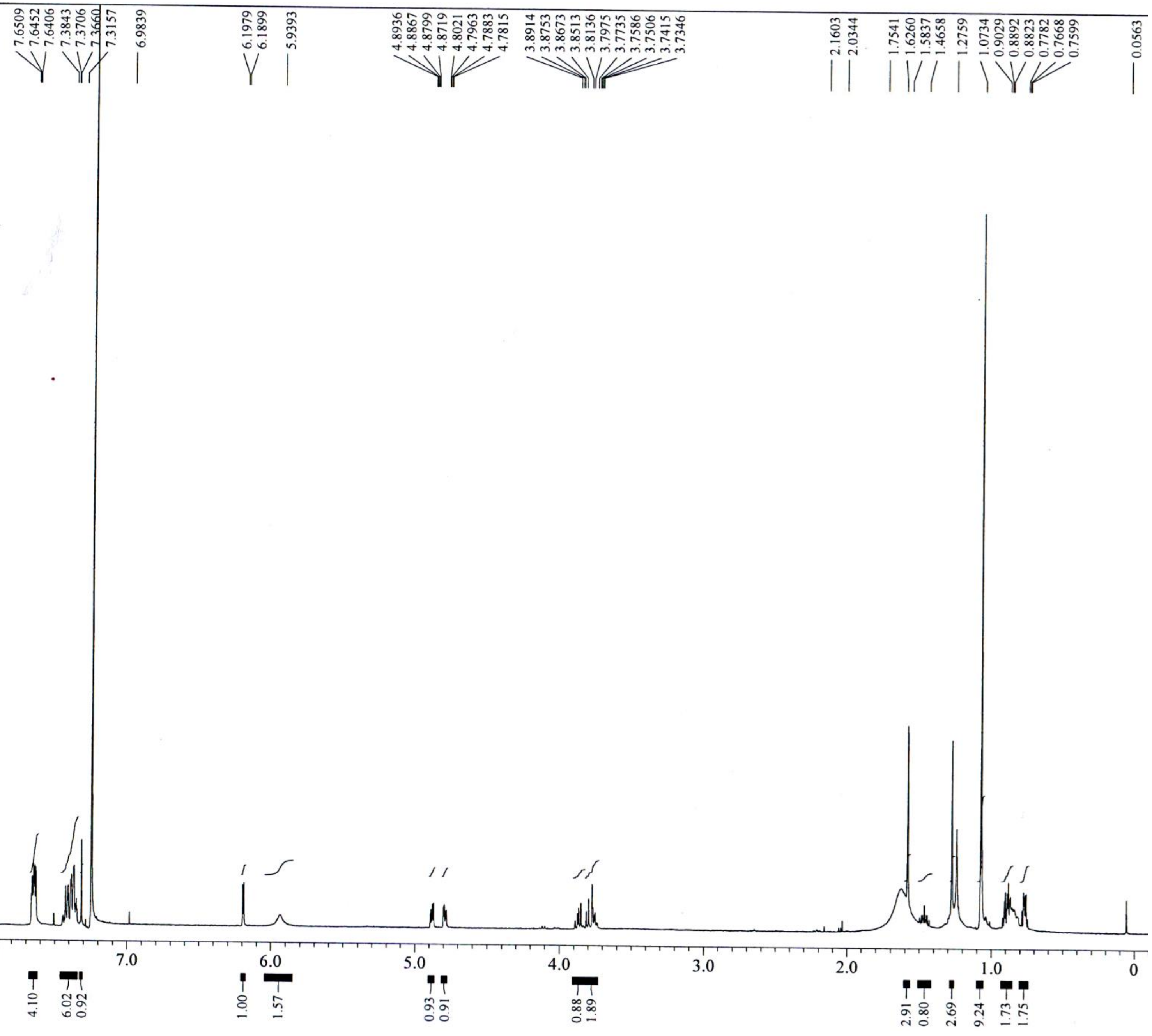

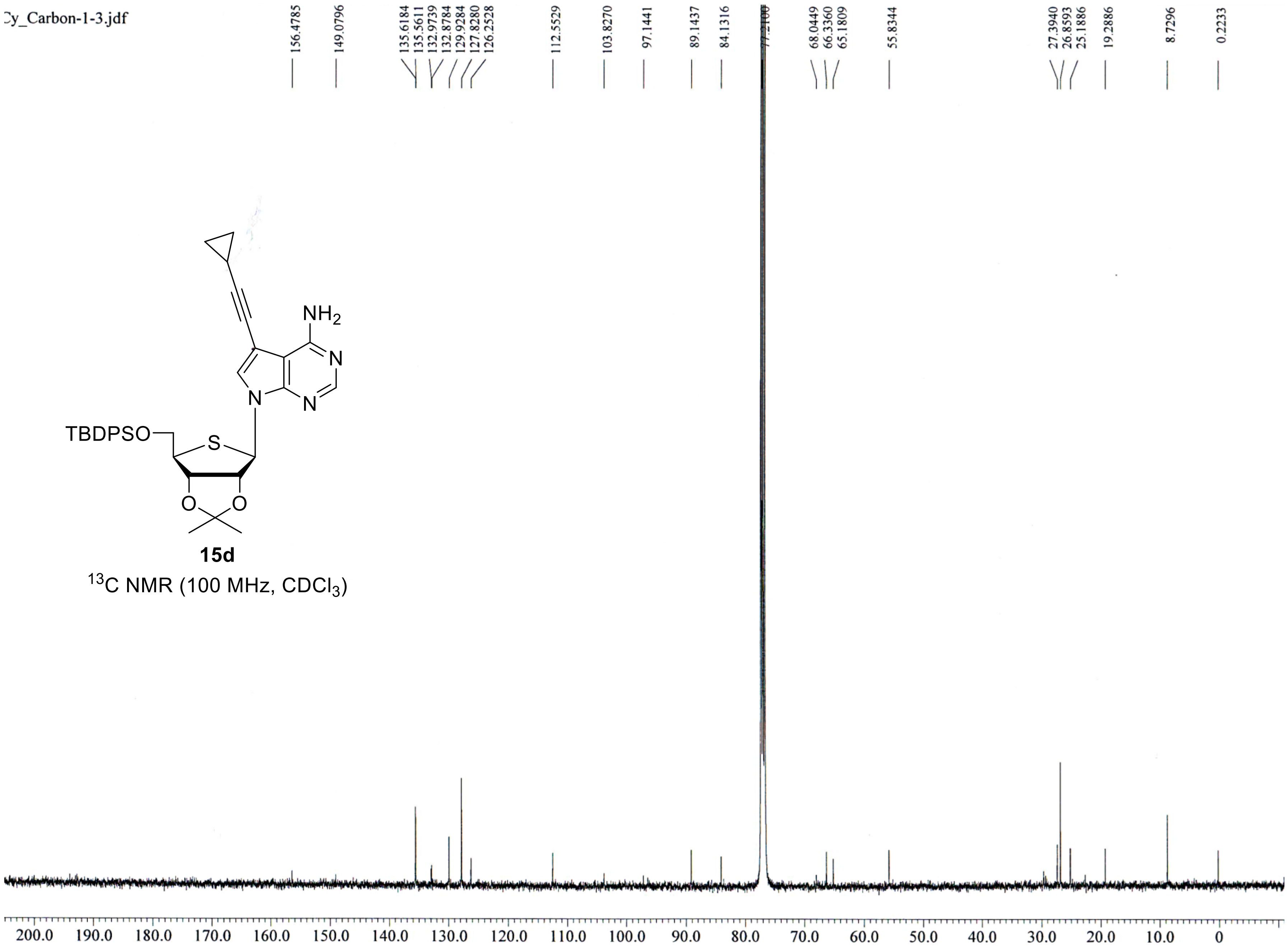

15d

^{13}C NMR (100 MHz, $CDCl_3$)

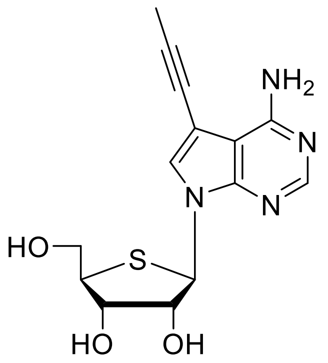

1h

^{1}H NMR (500 MHz, DMSO-d_6)

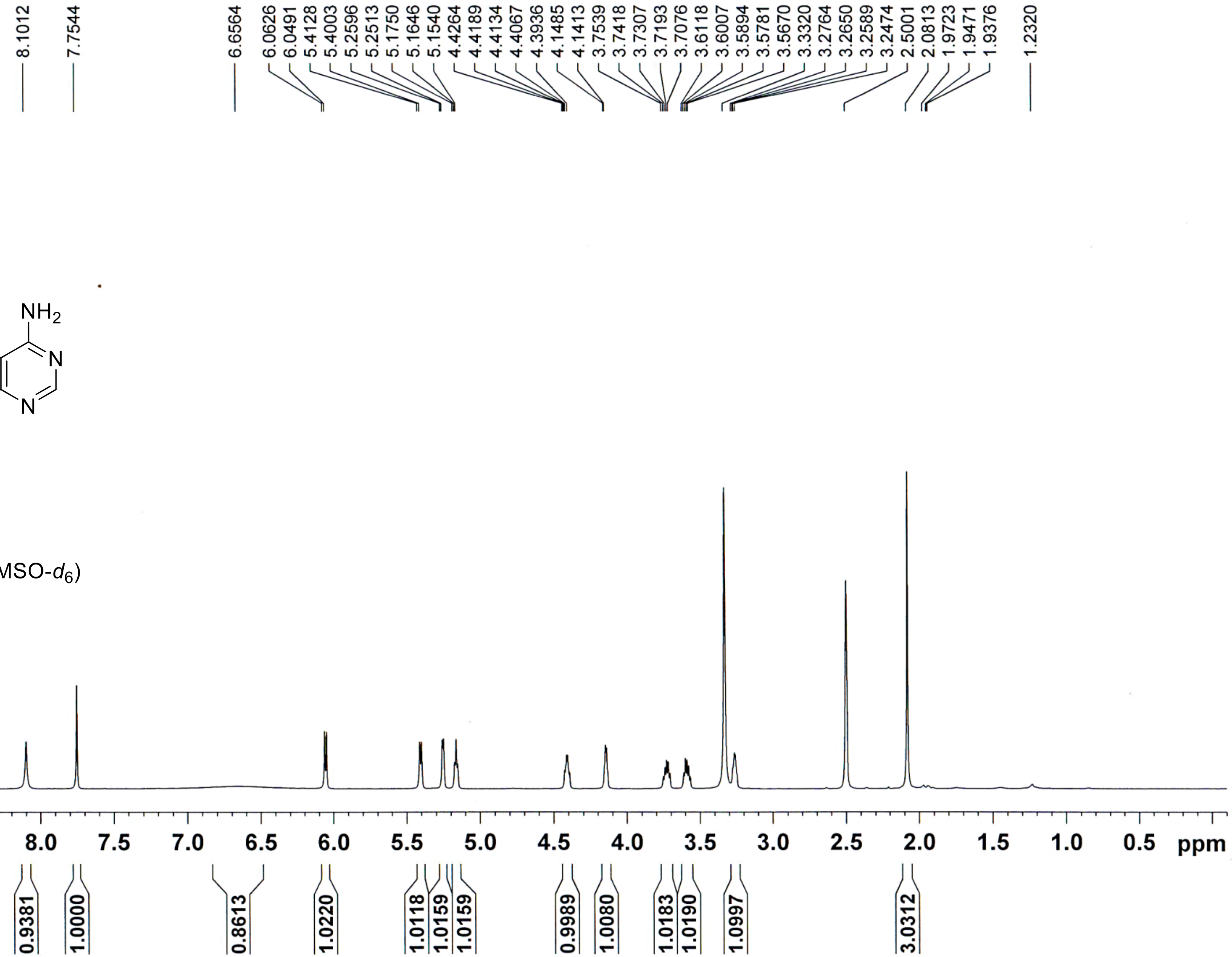

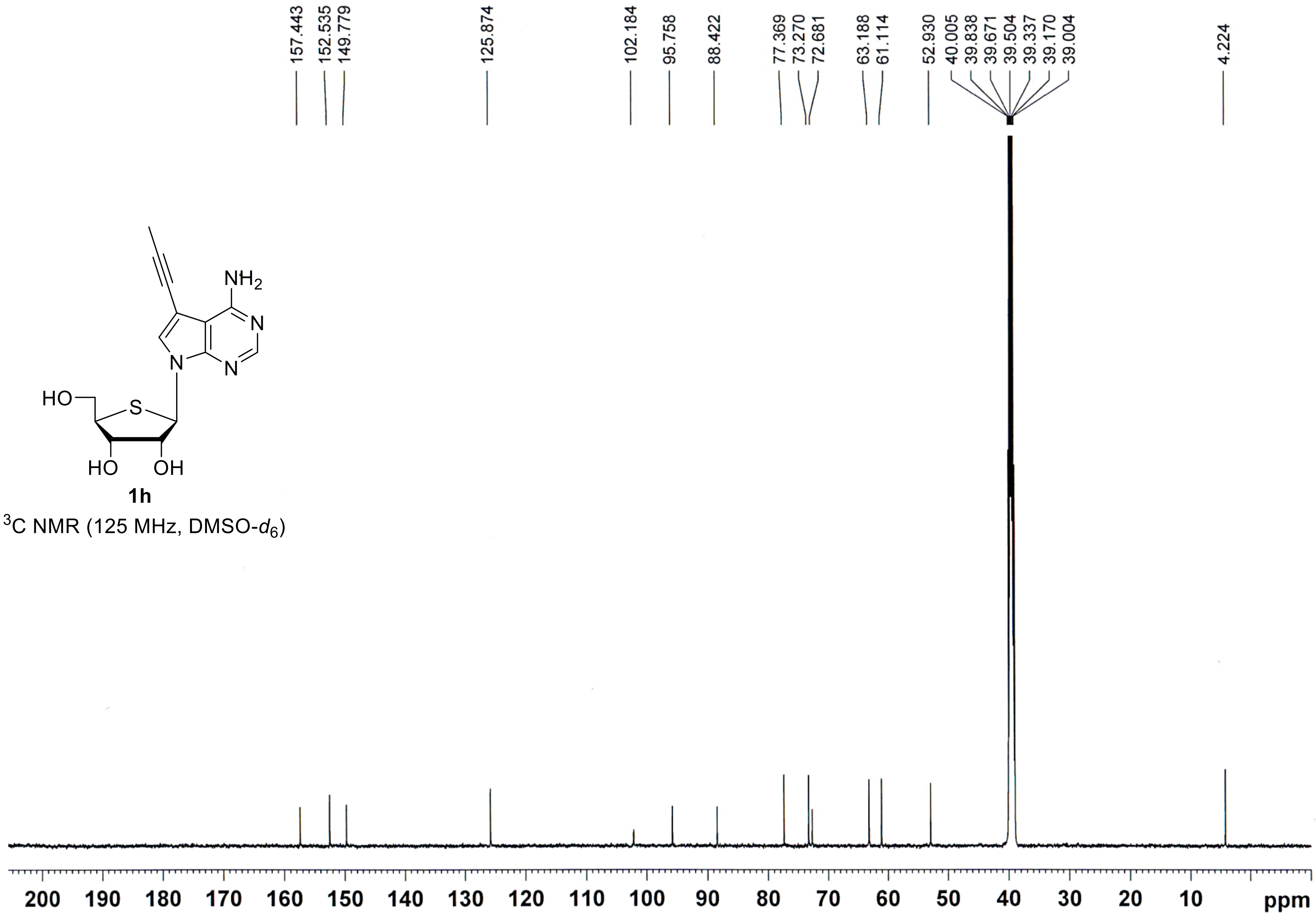

1h

^{13}C NMR (125 MHz, DMSO-d_6)

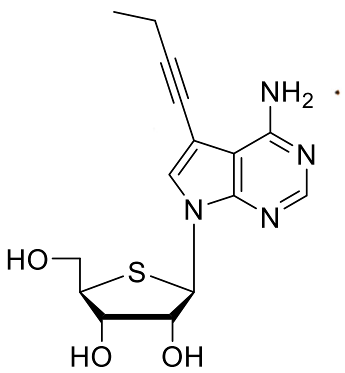

1i

^{1}H NMR (500 MHz, DMSO-d_6)

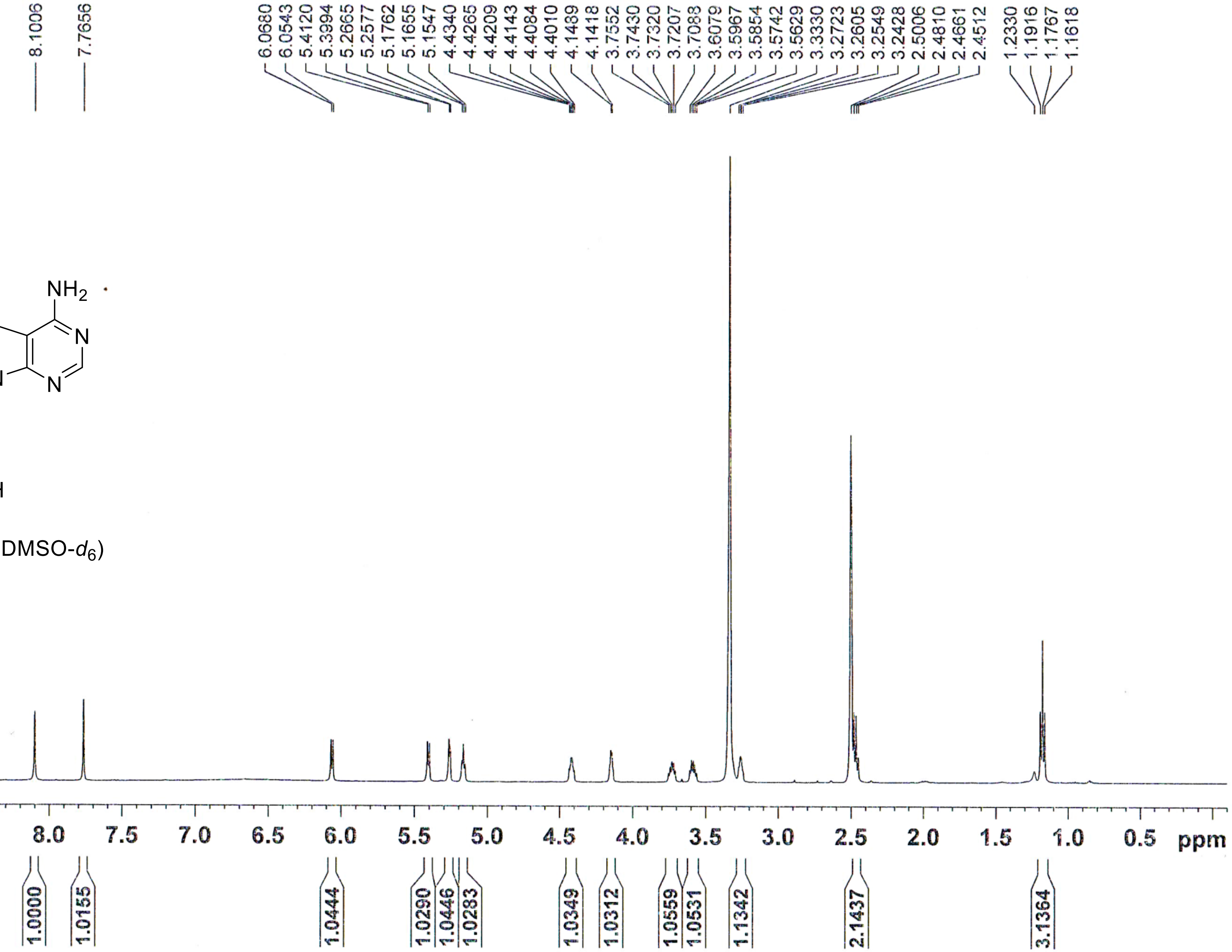

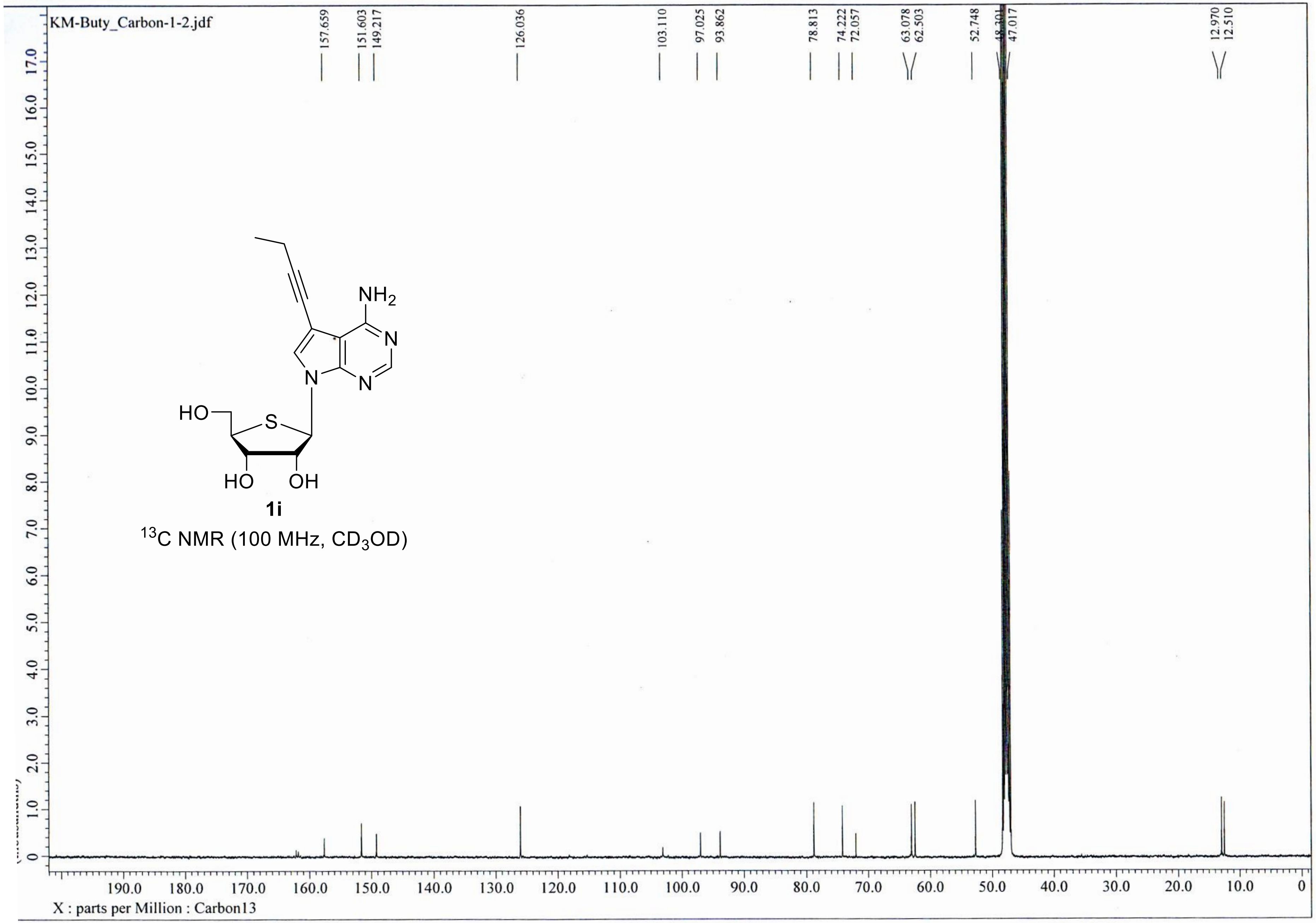

1i

^{13}C NMR (100 MHz, CD_3OD)

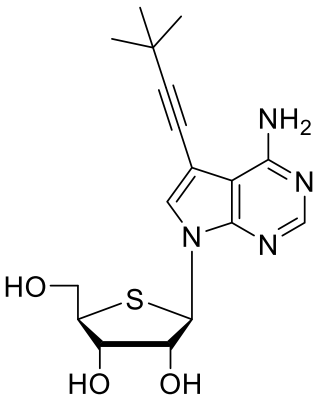

1j

^{1}H NMR (500 MHz, DMSO-d_6)

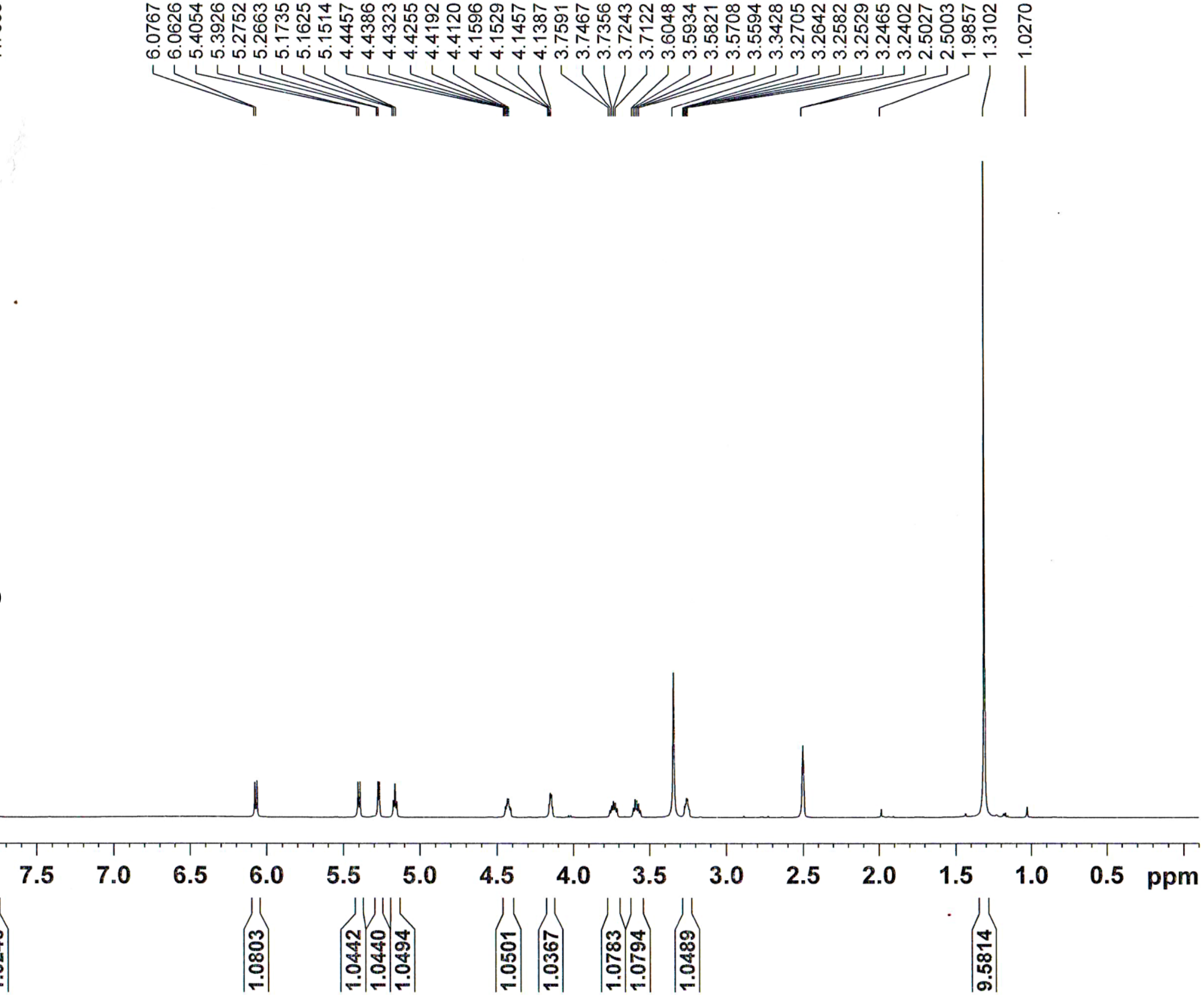

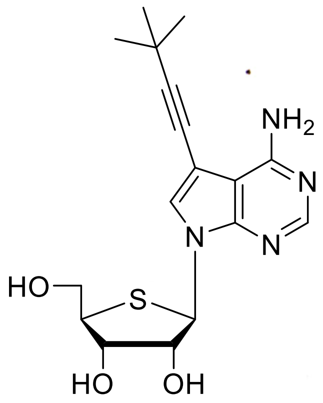

1j

^{13}C NMR (100 MHz, DMSO-d_6)

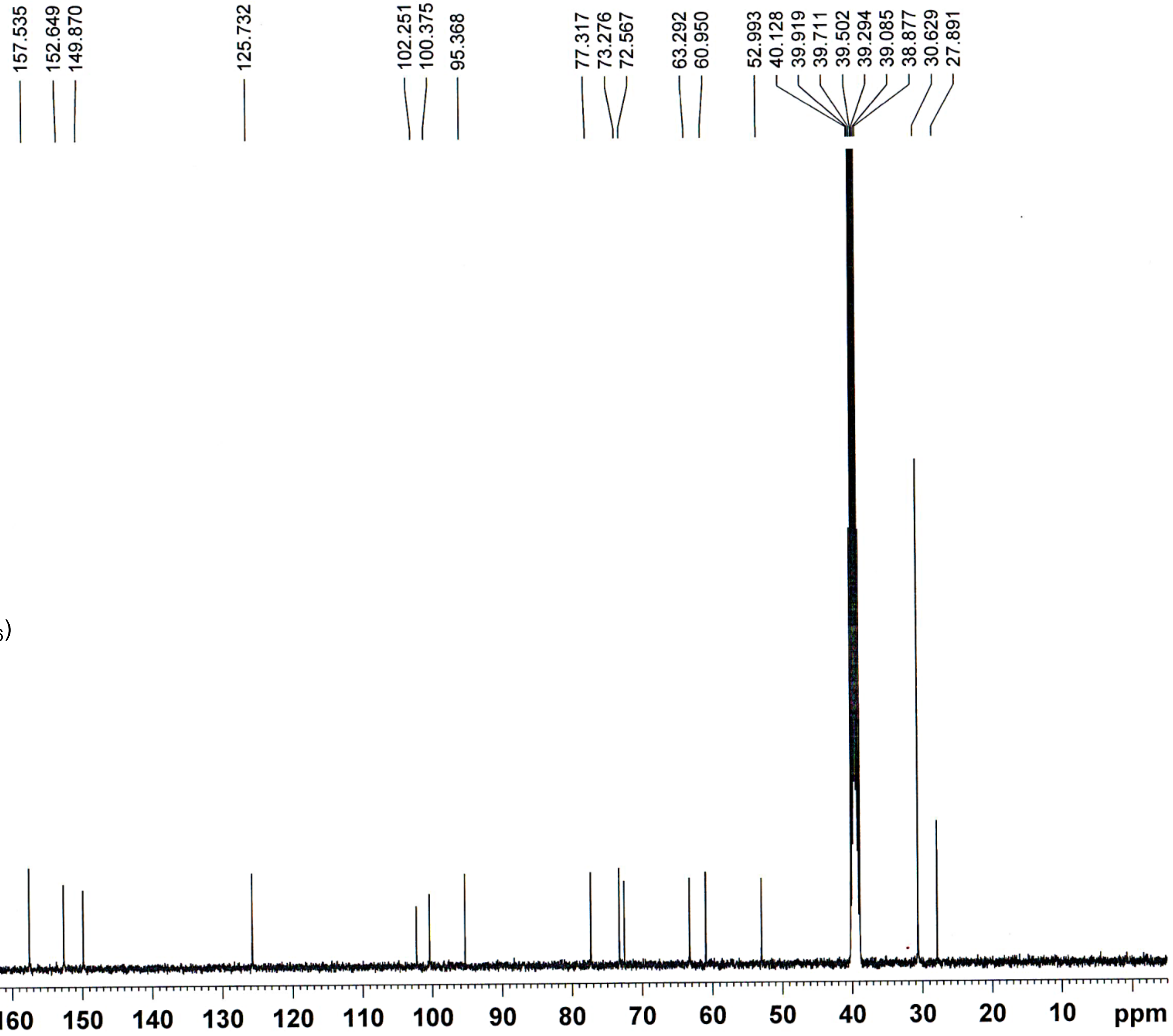

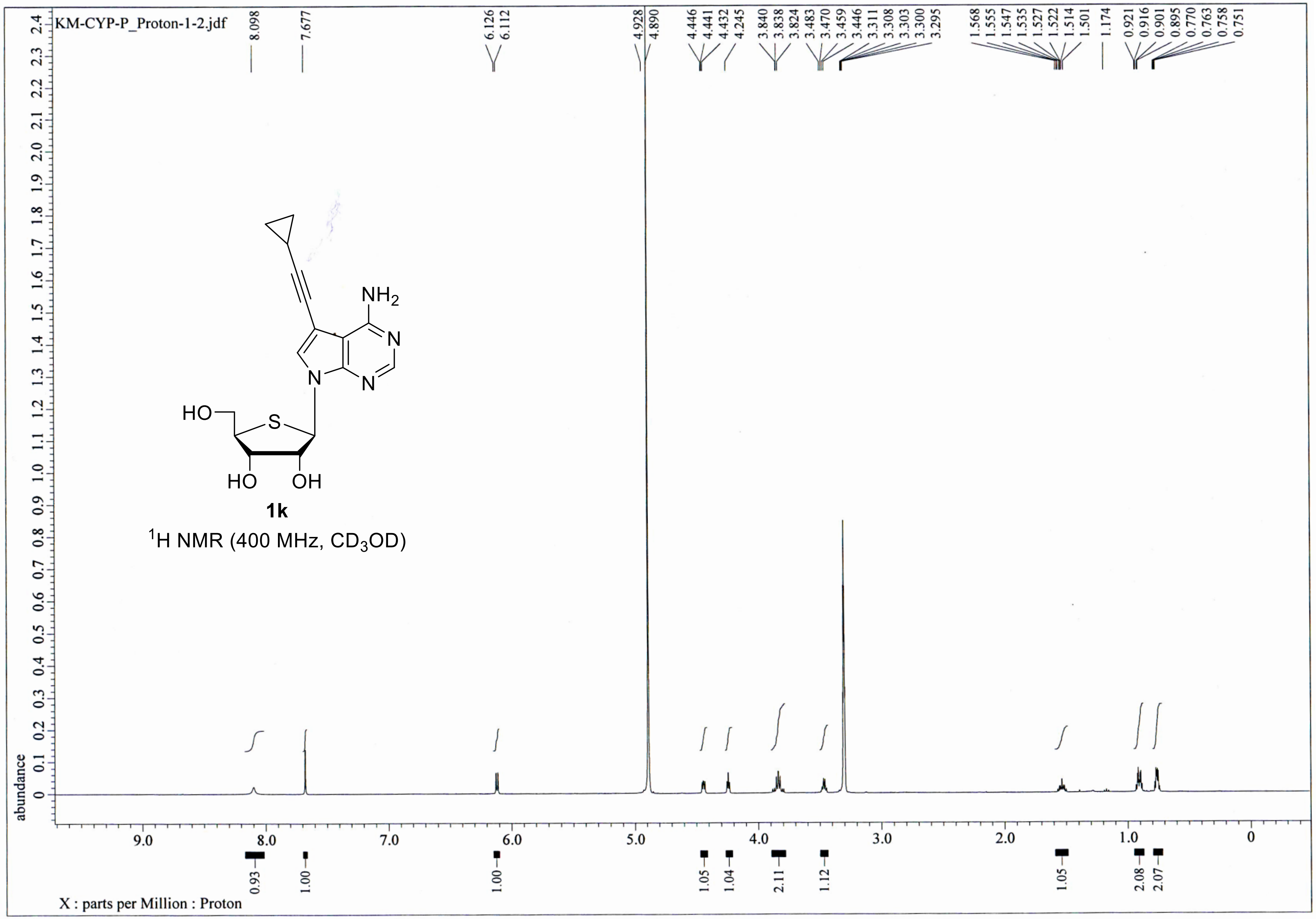
KM-CYP-P_Proton-1-2.jdf
8.098
7.677
6.126
6.112
4.928
4.890
4.446
4.441
4.432
4.245
3.840
3.838
3.824
3.483
3.470
3.459
3.446
3.311
3.308
3.303
3.300
3.295
1.568
1.555
1.547
1.535
1.527
1.522
1.514
1.501
1.174
0.921
0.916
0.901
0.895
0.770
0.763
0.758
0.751
NH2
N
N
N
HO
S
HO
OH
1k
1H NMR (400 MHz, CD3OD)
abundance
X : parts per Million : Proton
0.93
1.00
1.00
1.05
1.04
2.11
1.12
1.05
2.08
2.07

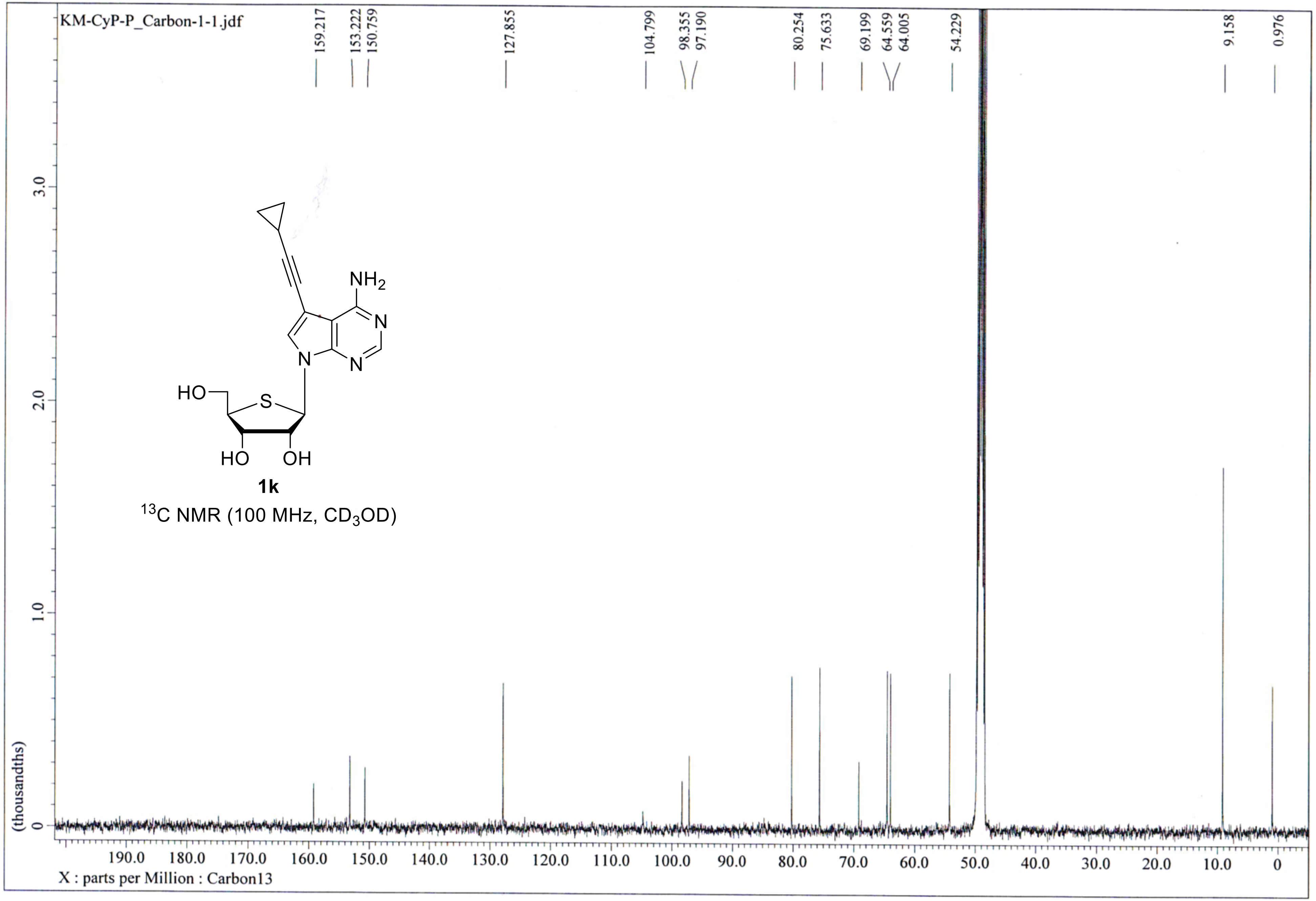
KM-CyP-P_Carbon-1-1.jdf
159.217
153.222
150.759
127.855
104.799
98.355
97.190
80.254
75.633
69.199
64.559
64.005
54.229
9.158
0.976
NH2
N
N
N
HO
S
HO
OH
1k
13C NMR (100 MHz, CD3OD)
(thousandths)
X : parts per Million : Carbon13

HRMS (ESI-Q-TOF) data for compound 1g

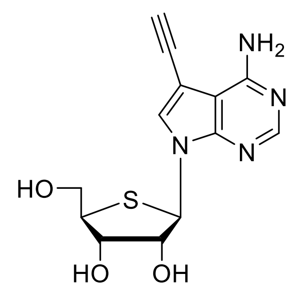

1g

Qualitative Compound Report

MS Zoomed Spectrum

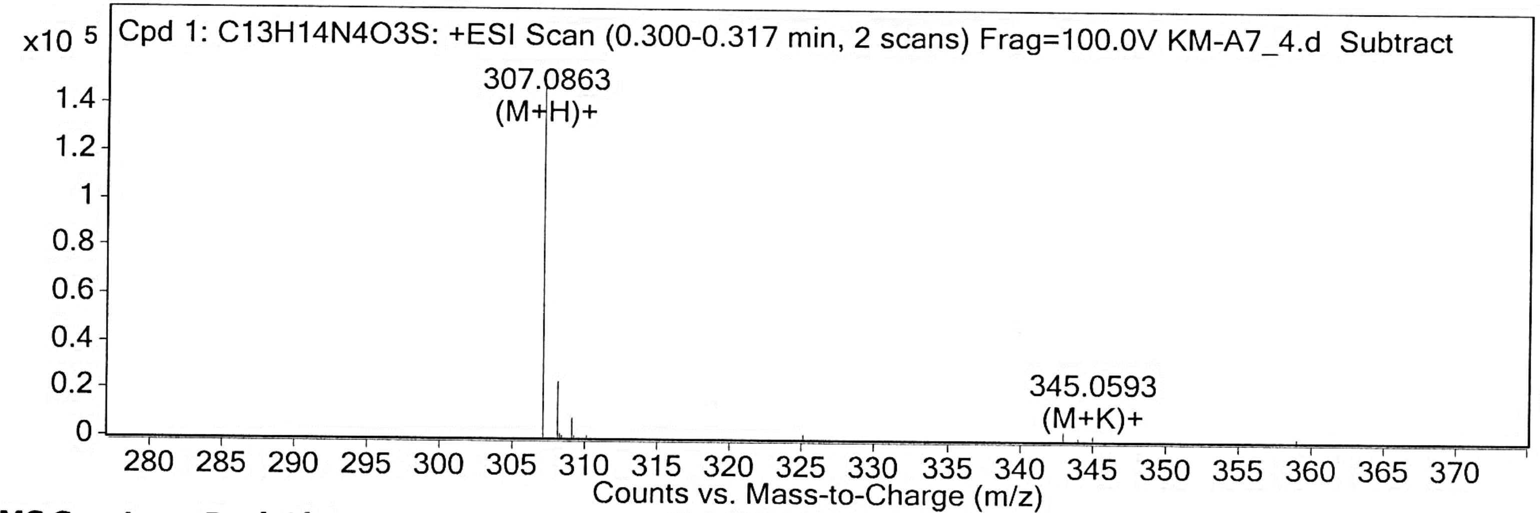

MS Spectrum Peak List

m/z	*Calc m/z*	Diff(ppm)	z	Abund	Formula	Ion
307.0863	307.0859	1.05		149838	C13 H15 N4 O3 S	(M+H)+
308.0893	308.0886	2.17		23738	C13 H15 N4 O3 S	(M+H)+
308.2138				1455		
308.2503				999		
308.292				632		
308.3751				516		
309.0846	309.0845	0.48		8038	C13 H15 N4 O3 S	(M+H)+
309.1713				938		
310.0859	310.0862	-0.95		1185	C13 H15 N4 O3 S	(M+H)+
345.0593	345.0418	50.81	1	1552	C13 H14 K N4 O3 S	(M+K)+

Single-Crystal X-ray crystallography data for compound 1g

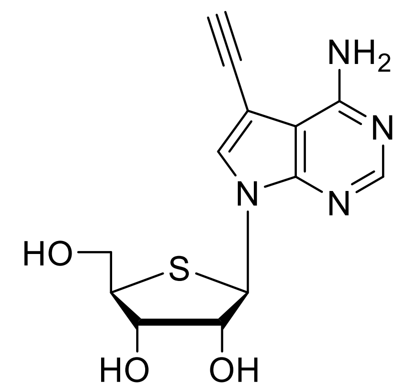

1g

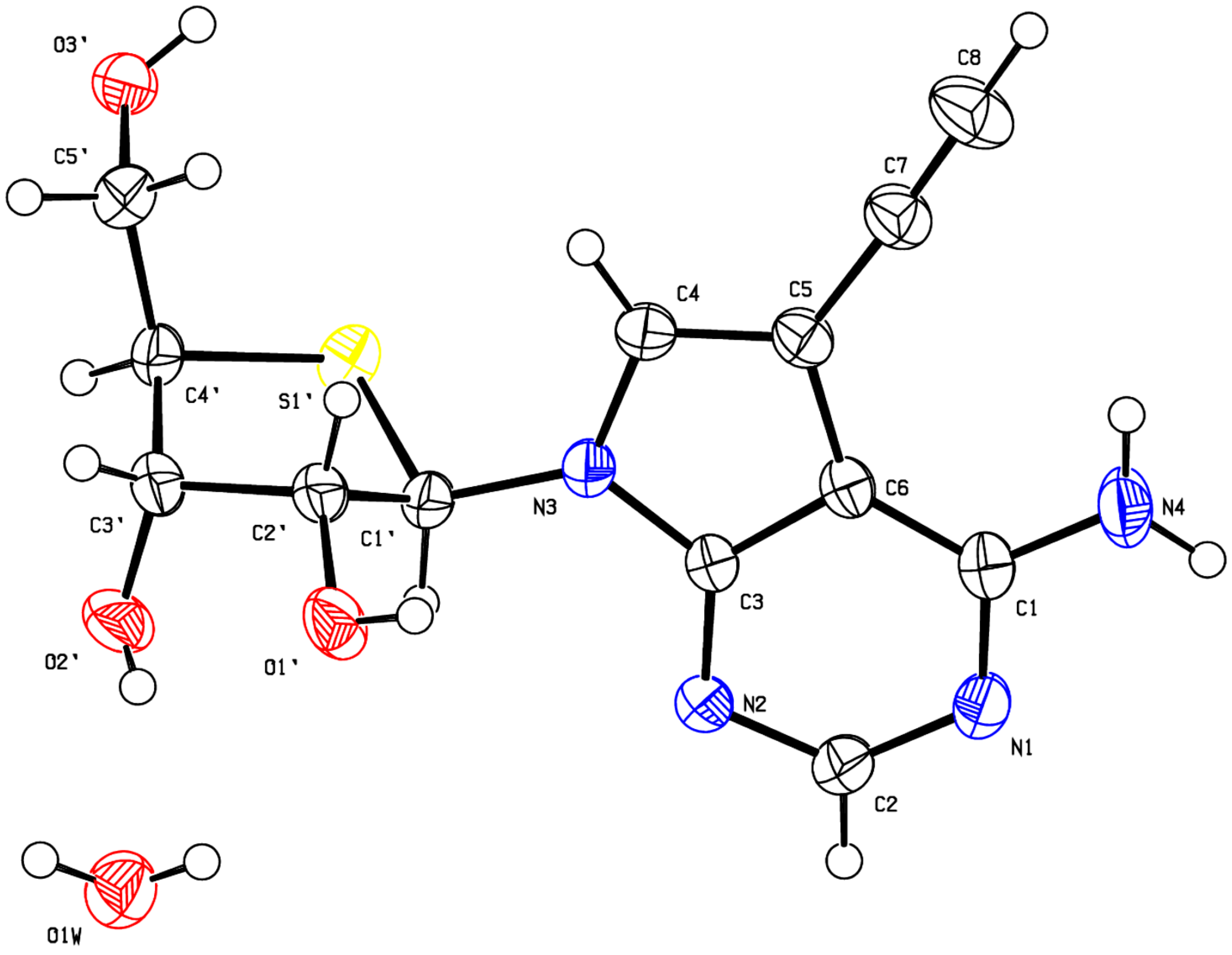

Fig S1. ORTEP diagram of compound **1g** showing thermal ellipsoid at 50% probability

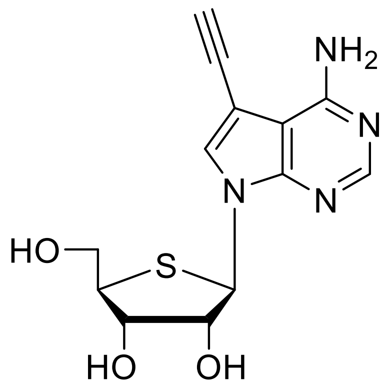

1g

Crystal structure data for $C_{13}H_{16}N_4O_4S$ (**1g**), M_r =324.36, triclinic, space group P1 (no. 1), a = 5.31660(10) Å, b = 7.5604(2) Å, c = 10.2329(2) Å, V = 362.147(15) Å^3, Z = 1, T = 294.8(2) K, ρ_{calc} = 1.487 g cm^{-3}, F(000) = 170.0, crystal dimension 0.227 × 0.1 × 0.049 mm^3, μ(CuKα) = 2.225 mm^{-1}, CuKα radiation (λ = 1.54184 Å). Of 18922 reflections collected in the 2θ range from 9.19 to 134.13° using an ω scan on a SuperNova, Dual, Cu at zero, AtlasS2 diffractometer, 2460 were unique reflections (R_{int} = 0.0230, R_{sigma} = 0.0141). Using Olex2, the structure was solved with the She1XT structure solution program using Intrinsic Phasing and refined with the She1XL refinement package using Least Squares minimization. wR_2 = 0.0658, R_1 = 0.0239, GOF = 1.102, and max/min residual electron density 0.13/-0.14 e Å^{-3}. Flack x parameter = -0.002 (9). Further details of the crystal structure investigation(s) may be obtained from the Cambridge Crystallographic Data Centre (CCDC), 12 Union Road, Cambridge, CB2 1EZ (UK); Tel: (+44)1223-336-408, Fax: (+44)1223-336-033, e-mail: deposit@ccdc.cam.ac.uk) on quoting the depository no. **CCDC 1575257**.

Conformational analysis of the sugar ring in compound 1g

ν0	ν1	ν2	ν3	ν4	P	νmax	χ	γ	TYPE
-19.0	42.05	-50.83	35.38	-9.11	173.96	51.11	-118.3	-179.12	PUR, C2'-endo

Definition of parameters:	
ν0-ν4 - Dihedral angles	P - Pseudorotation phase angle
PUR - Purine type base (χ: S1'-C1'-N3-C3)	γ - O3'-C5'-C4'-C3'

Pseudorotational angle P = 174.0°

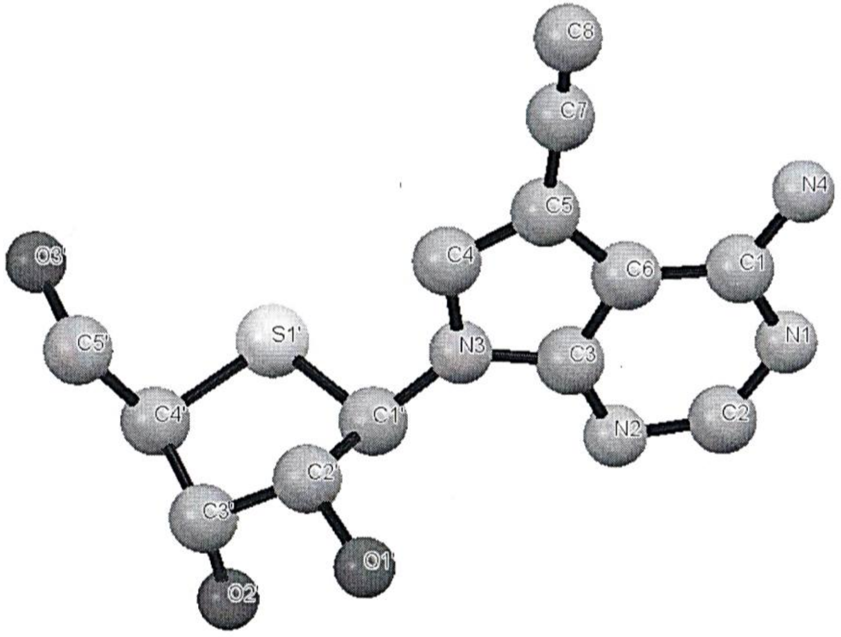

HPLC purity data of 1a

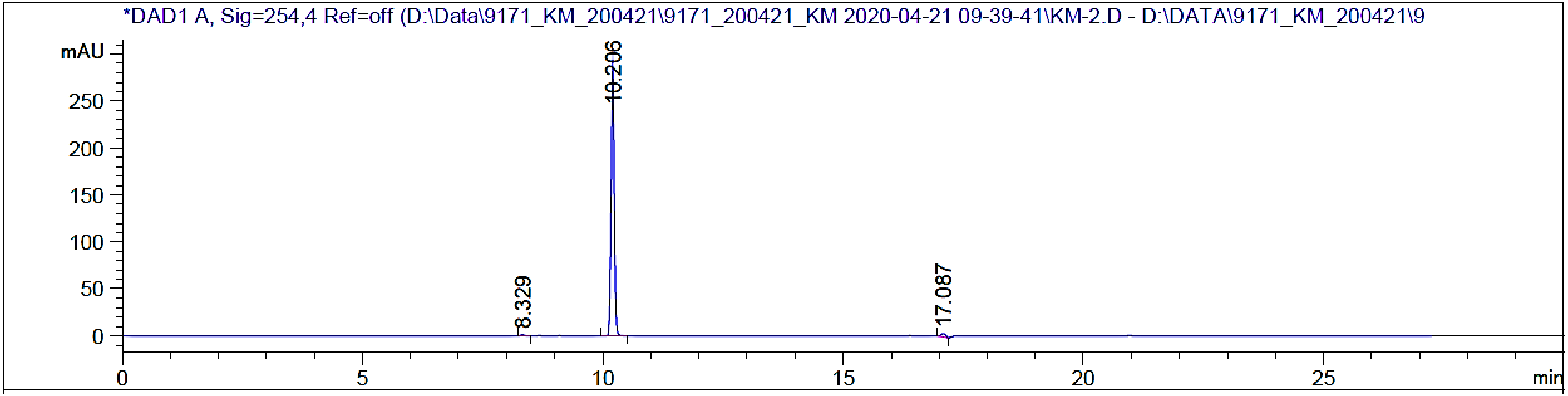

Peak #	RetTime [min]	Type	Width [min]	Area [mAU*s]	Height [mAU]	Area %
1	8.329	BB	0.0737	6.60799	1.38433	0.4607
2	10.206	BB	0.0726	1404.34875	300.04871	97.9183
3	17.087	BB	0.1039	23.24776	3.57208	1.6210

Totals : 1434.20451 305.00512

HPLC purity data of 1b

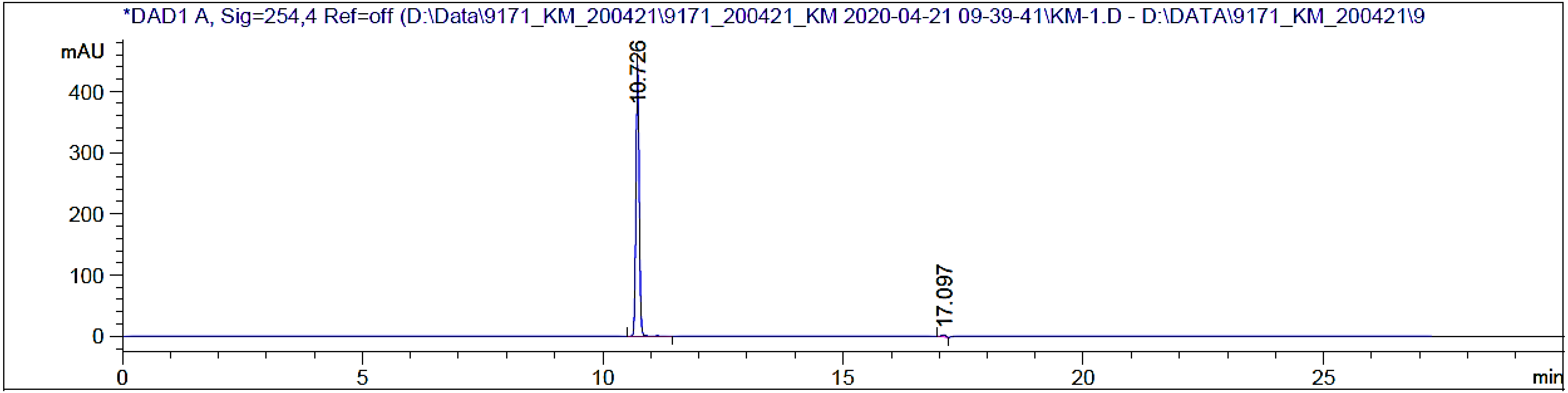

Peak #	RetTime [min]	Type	Width [min]	Area [mAU*s]	Height [mAU]	Area %
1	10.726	BV R	0.0736	2195.87183	461.01913	98.9904
2	17.097	BB	0.1019	22.39597	3.53589	1.0096
Totals :				2218.26780	464.55502	

HPLC purity data of 1c

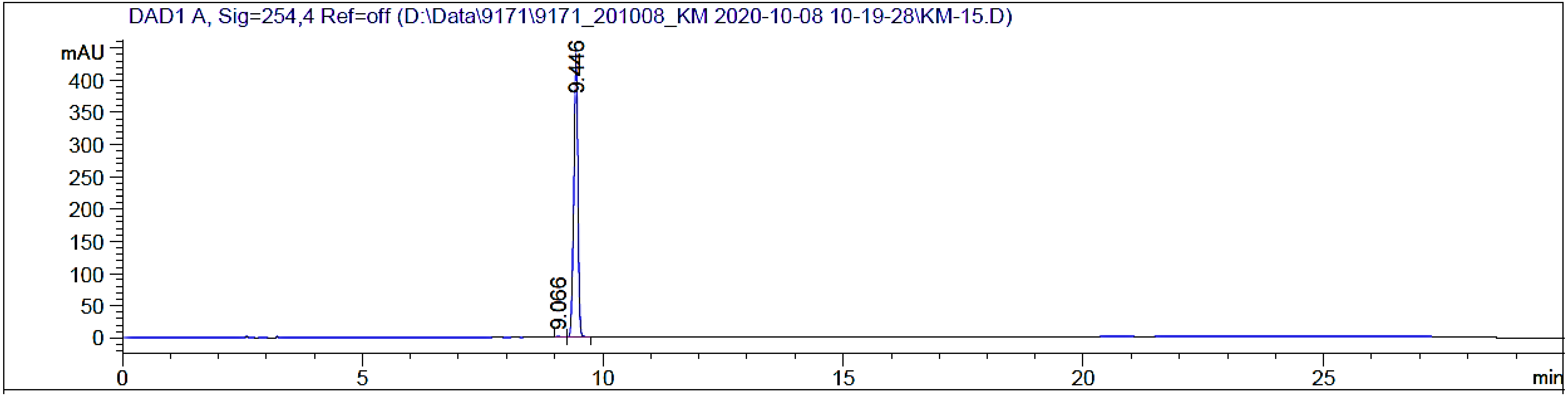

Peak #	RetTime [min]	Type	Width [min]	Area [mAU*s]	Height [mAU]	Area %
1	9.066	BB	0.0708	6.64823	1.46846	0.2508
2	9.446	BB	0.0902	2643.77856	439.59253	99.7492

Totals : 2650.42679 441.06099

HPLC purity data of 1d

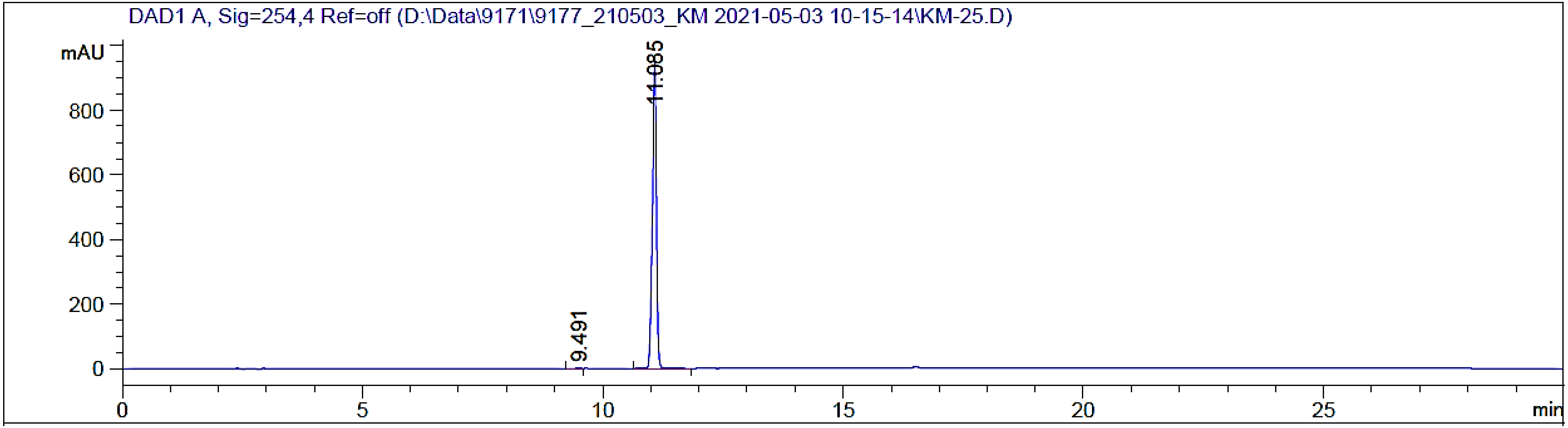

Peak #	RetTime [min]	Type	Width [min]	Area [mAU*s]	Height [mAU]	Area %
1	9.491	BV	0.0913	12.72995	2.02748	0.2218
2	11.085	BB	0.0890	5726.36230	967.73749	99.7782

Totals : 5739.09226 969.76497

HPLC purity data of 1e

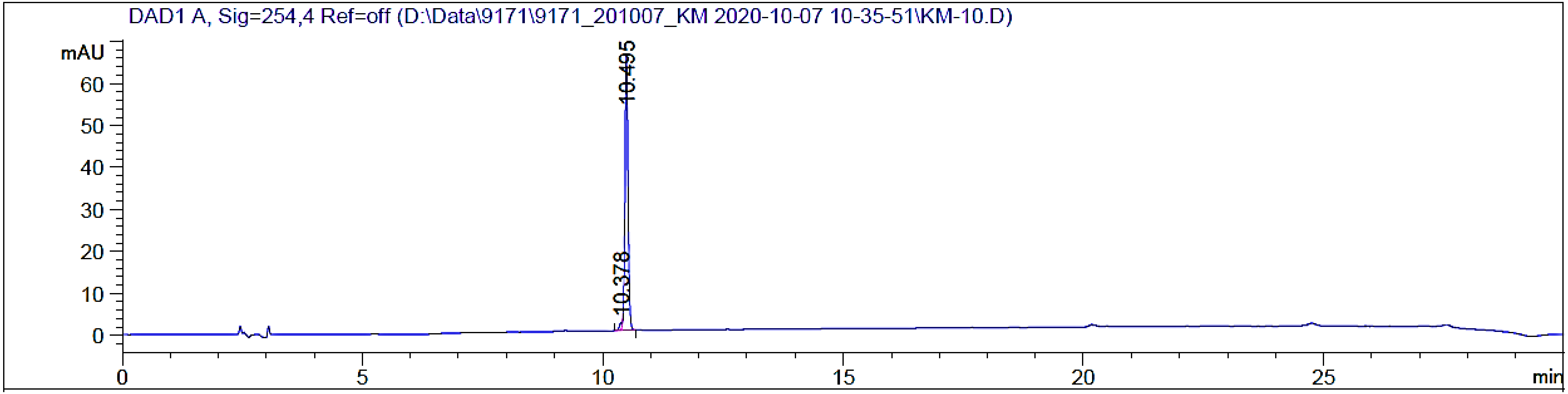

Peak #	RetTime [min]	Type	Width [min]	Area [mAU*s]	Height [mAU]	Area %
1	10.378	BV E	0.0561	6.35986	1.68225	2.0317
2	10.495	VB R	0.0701	306.66940	66.09574	97.9683
Totals :				313.02926	67.77799	

HPLC purity data of 1f

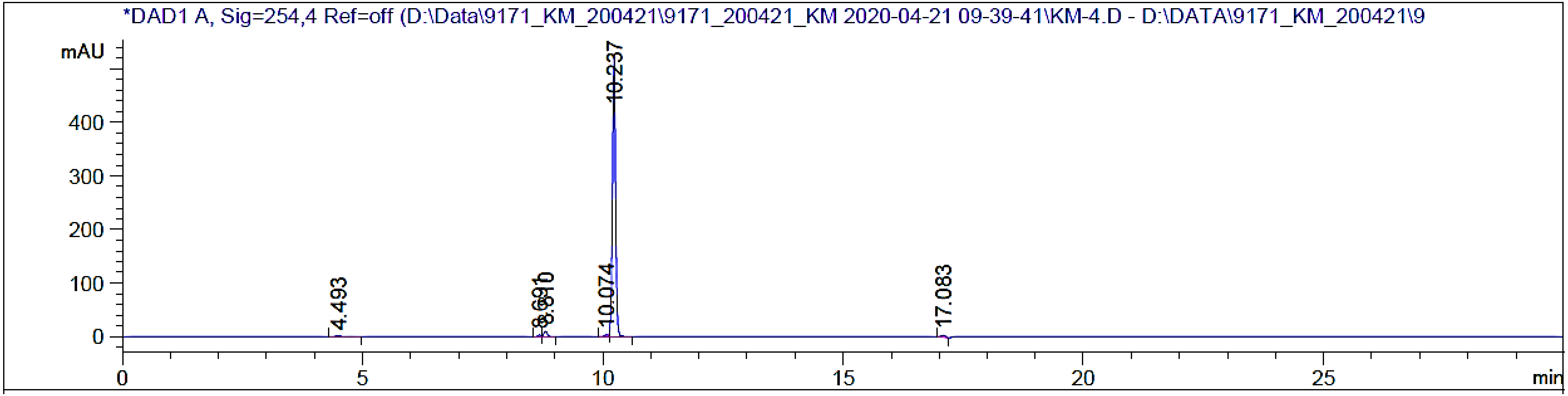

Peak #	RetTime [min]	Type	Width [min]	Area [mAU*s]	Height [mAU]	Area %
1	4.493	BB	0.1257	10.35117	1.28913	0.3912
2	8.691	BV	0.0705	15.31487	3.27851	0.5788
3	8.810	VB	0.0759	51.18538	10.30826	1.9343
4	10.074	BV E	0.0656	15.44302	3.63367	0.5836
5	10.237	VB R	0.0739	2530.55591	527.73730	95.6304
6	17.083	BB	0.1066	23.33294	3.55428	0.8818
Totals :				2646.18329	549.80114	

HPLC purity data of 1g

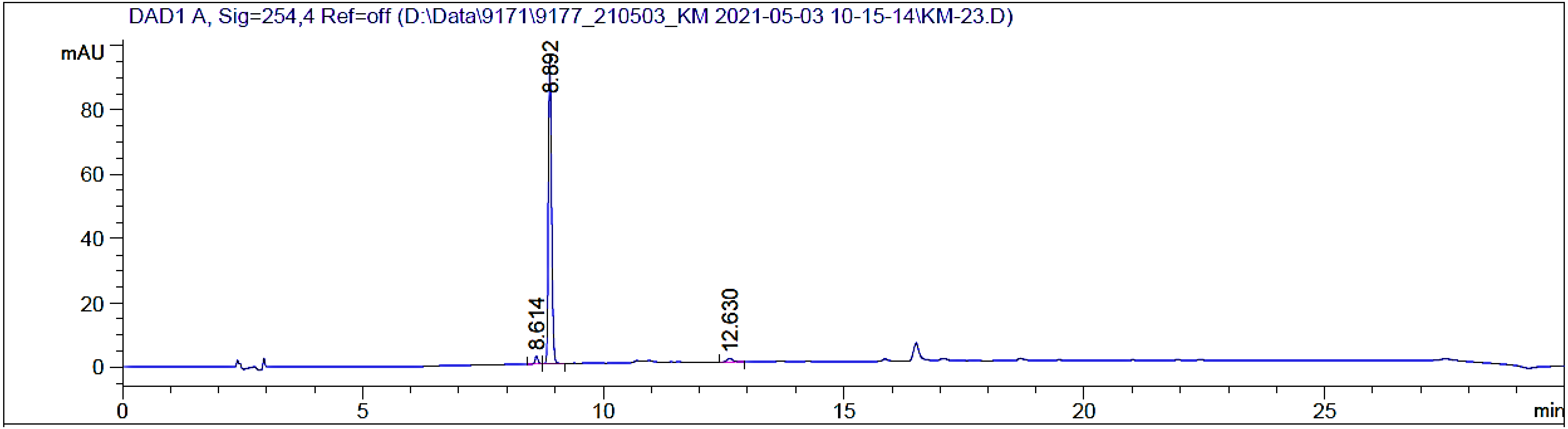

Peak #	RetTime [min]	Type	Width [min]	Area [mAU*s]	Height [mAU]	Area %
1	8.614	BB	0.0699	10.65508	2.39596	2.3159
2	8.892	BB	0.0715	438.71045	95.74940	95.3525
3	12.630	BB	0.1651	10.72762	1.01638	2.3316

Totals :				460.09315	99.16173	

HPLC purity data of 1h

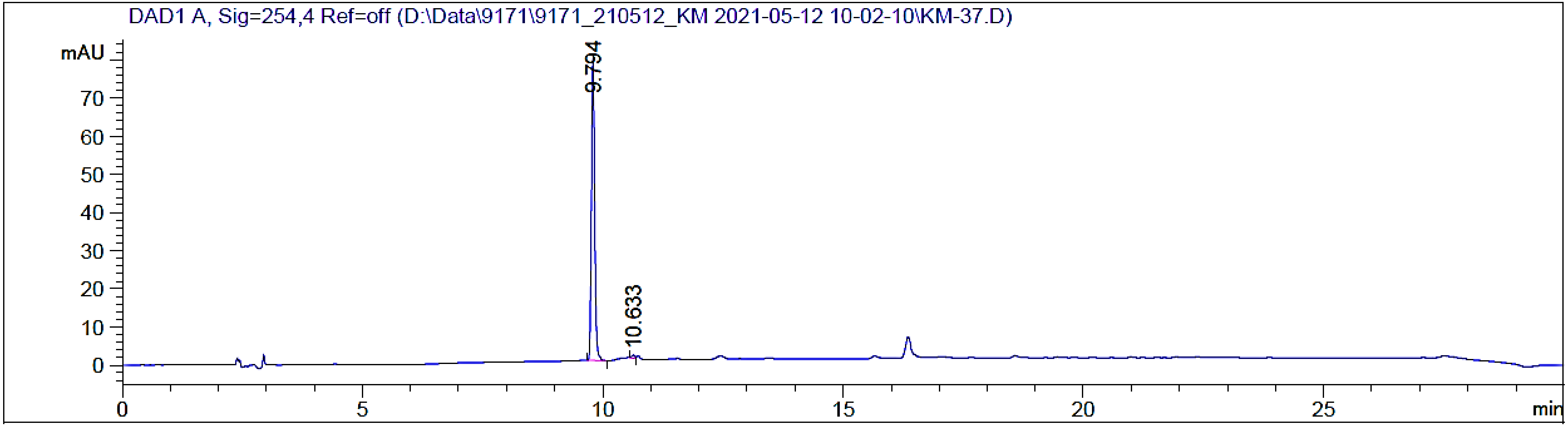

Peak #	RetTime [min]	Type	Width [min]	Area [mAU*s]	Height [mAU]	Area %
1	9.794	BB	0.0690	364.31149	80.18752	98.9838
2	10.633	BV	0.0692	3.74005	8.20275e-1	1.0162
Totals :				368.05154	81.00780	

HPLC purity data of 1i

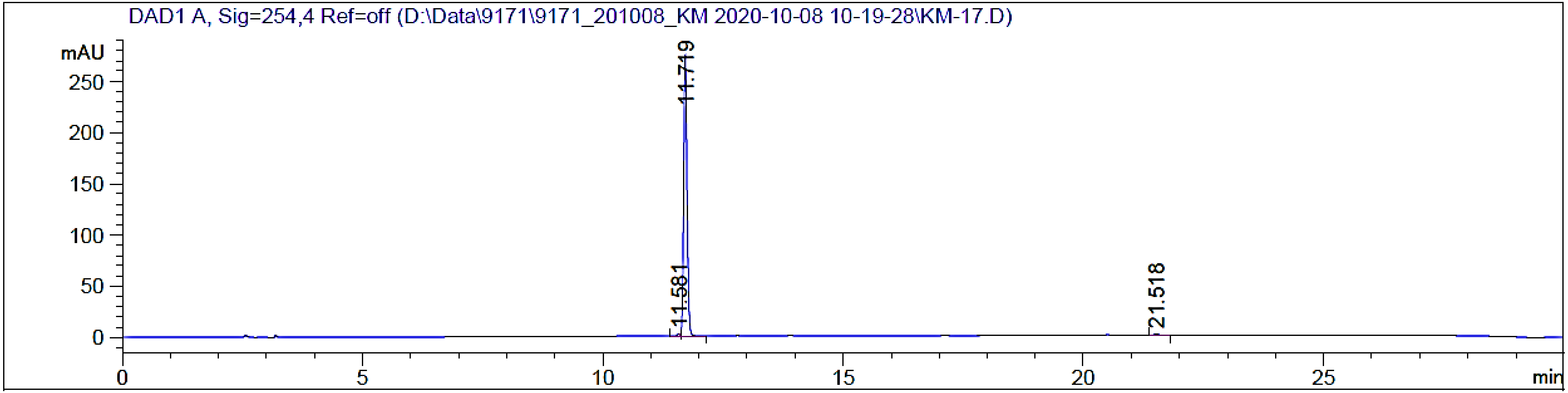

Peak #	RetTime [min]	Type	Width [min]	Area [mAU*s]	Height [mAU]	Area %
1	11.581	BV E	0.0600	7.97739	2.02500	0.6025
2	11.719	VB R	0.0713	1306.85962	275.95297	98.6961
3	21.518	BB	0.0928	9.28758	1.53137	0.7014

Totals : 1324.12458 279.50934

HPLC purity data of 1j

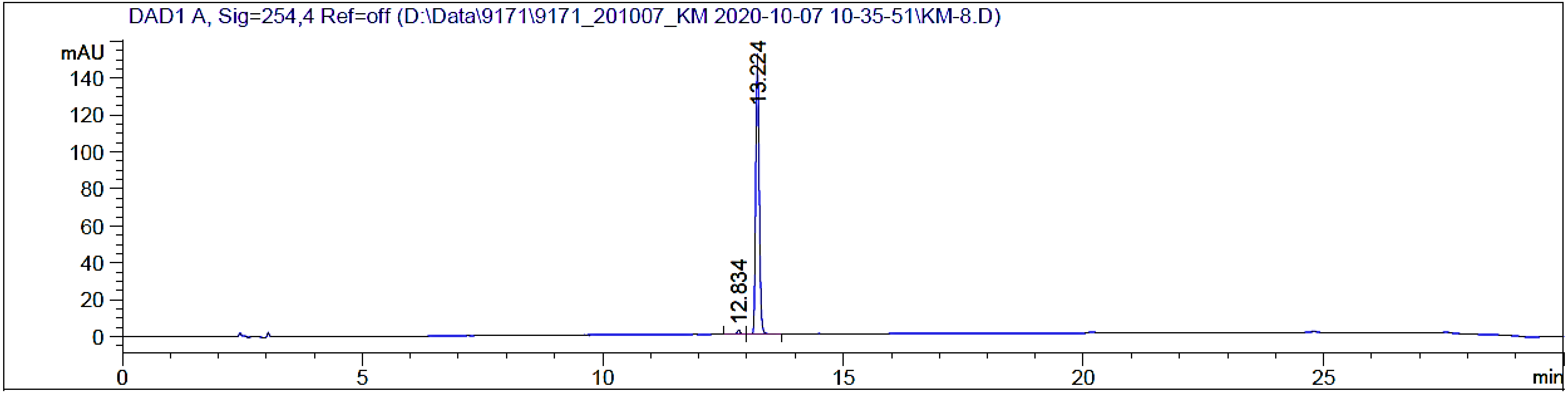

Peak #	RetTime [min]	Type	Width [min]	Area [mAU*s]	Height [mAU]	Area %
1	12.834	BB	0.0756	10.97668	2.22396	1.4582
2	13.224	BB	0.0769	741.76349	152.00572	98.5418

Totals : 752.74017 154.22968

HPLC purity data of 1k

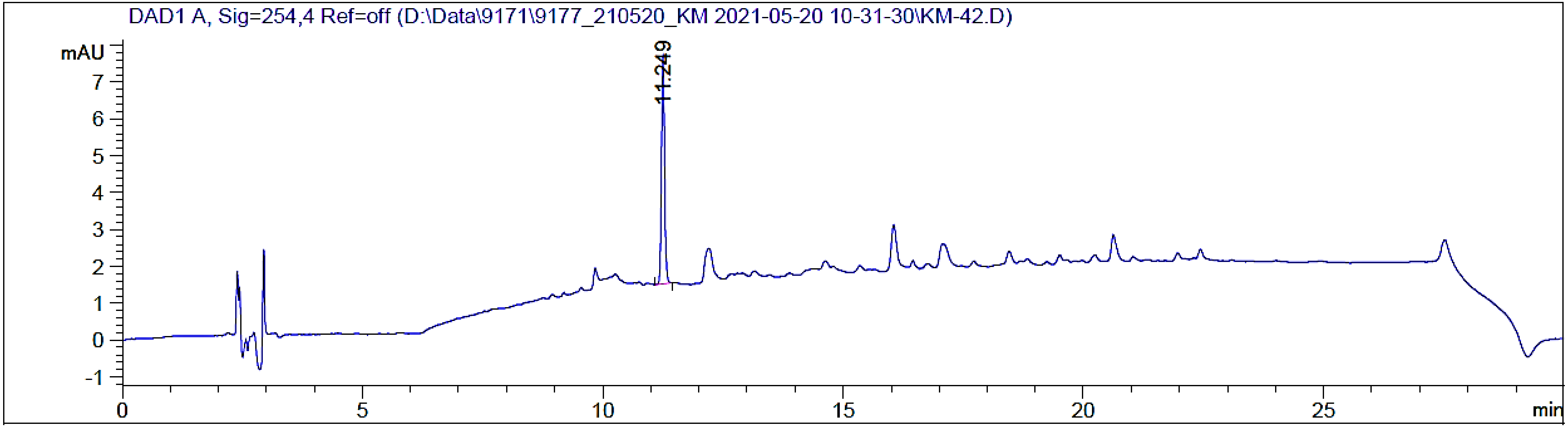

Peak #	RetTime [min]	Type	Width [min]	Area [mAU*s]	Height [mAU]	Area %
1	11.249	BB	0.0696	28.49407	6.19632	100.0000

Totals : 28.49407 6.19632

Table S1. Kinome scan data of compound 1g at 1000 nM[a]

DiscoveRx Gene Symbol	Entrez Gene Symbol	Compound 1g (% inhibition)
ABL1(E255K)-phosphorylated	ABL1	18.0
ABL1(T315I)-phosphorylated	ABL1	65.0
ABL1-nonphosphorylated	ABL1	39.0
ABL1-phosphorylated	ABL1	35.0
ACVR1B	ACVR1B	29.0
ADCK3	CABC1	8.0
AKT1	AKT1	7.0
AKT2	AKT2	4.0
ALK	ALK	31.0
AURKA	AURKA	22.0
AURKB	AURKB	0.0
AXL	AXL	7.0
BMPR2	BMPR2	1.0
BRAF	BRAF	0.0
BRAF(V600E)	BRAF	0.0
BTK	BTK	0.0
CDK11	CDK19	72.1
CDK2	CDK2	9.0
CDK3	CDK3	18.0
CDK7	CDK7	52.3
CDK9	CDK9	4.0
CHEK1	CHEK1	0.0
CSF1R	CSF1R	20.0

CSNK1D	CSNK1D	91.3
CSNK1G2	CSNK1G2	54.0
DCAMKL1	DCLK1	6.0
DYRK1B	DYRK1B	83.0
EGFR	EGFR	0.0
EGFR(L858R)	EGFR	0.0
EPHA2	EPHA2	28.0
ERBB2	ERBB2	15.0
ERBB4	ERBB4	3.0
ERK1	MAPK3	4.0
FAK	PTK2	0.0
FGFR2	FGFR2	12.0
FGFR3	FGFR3	10.0
FLT3	FLT3	27.0
GSK3B	GSK3B	0.0
IGF1R	IGF1R	7.0
IKK-alpha	CHUK	10.0
IKK-beta	IKBKB	0.0
INSR	INSR	5.0
JAK2(JH1domain-catalytic)	JAK2	35.0
JAK3(JH1domain-catalytic)	JAK3	0.0
JNK1	MAPK8	17.0
JNK2	MAPK9	0.0
JNK3	MAPK10	13.0

KIT	KIT	1.0
KIT(D816V)	KIT	22.0
KIT(V559D,T670I)	KIT	17.0
LKB1	STK11	2.0
MAP3K4	MAP3K4	29.0
MAPKAPK2	MAPKAPK2	0.0
MARK3	MARK3	0.0
MEK1	MAP2K1	74.0
MEK2	MAP2K2	69.0
MET	MET	4.0
MKNK1	MKNK1	0.0
MKNK2	MKNK2	62.0
MLK1	MAP3K9	7.0
p38-alpha	MAPK14	11.0
p38-beta	MAPK11	3.0
PAK1	PAK1	18.0
PAK2	PAK2	44.0
PAK4	PAK4	50.0
PCTK1	CDK16	0.0
PDGFRA	PDGFRA	0.0
PDGFRB	PDGFRB	8.0
PDPK1	PDPK1	11.0
PIK3C2B	PIK3C2B	1.0
PIK3CA	PIK3CA	7.0

PIK3CG	PIK3CG	0.0
PIM1	PIM1	37.0
PIM2	PIM2	3.0
PIM3	PIM3	25.0
PKAC-alpha	PRKACA	11.0
PLK1	PLK1	0.0
PLK3	PLK3	0.0
PLK4	PLK4	0.0
PRKCE	PRKCE	0.0
RAF1	RAF1	8.0
RET	RET	11.0
RIOK2	RIOK2	0.0
ROCK2	ROCK2	27.0
RSK2(Kin.Dom.1-N-terminal)	RPS6KA3	55.0
SNARK	NUAK2	0.0
SRC	SRC	6.0
SRPK3	SRPK3	6.0
TGFBR1	TGFBR1	11.0
TIE2	TEK	6.0
TRKA (NTRK1)	NTRK1	95.1
TSSK1B	TSSK1B	16.0
TYK2(JH1domain-catalytic)	TYK2	70.0
ULK2	ULK2	0.0
VEGFR2	KDR	30.0
ZAP70	STK32C	0.0

[a]Compound **1g** was screened at 1000 nM against 96 kinases using DiscoveRx Kinome Scan. The results for binding interactions are reported as % inhibition, where higher values indicate strong affinity.

Table S2. Kinase inhibition profile of 1a-k against TRKA, CK1δ, and DYRK1A/1B

Compound no.	IC_{50} (K_i) (nM)[a]			
	**TRKA**	**CK1δ**	**DYRK1A**	**DYRK1B**
**1a**	37 (74)	123 (123)	15 (30)	14 (14)
**1b**	11 (22)	18 918)	166 (332)	117 (117)
**1c**	474 (948)	77 (77)	112 (224)	86 (86)
**1d**	157 (314)	27 (27)	357 (714)	288 (288)
**1e**	> 10,000	> 10,000	> 10,000	> 10,000
**1f**	> 10,000	910 (910)	> 10,000	> 10,000
**1g**	148 (296)	80 (80)	43 (86)	33 (33)
**1h**	293 (586)	970 (970)	1,255 (2,510)	1,069 (1,069)
**1i**	573 (1,146)	430 (430)	2,981 (5,962)	1,343 (1,343)
**1j**	958 (786)	1,321 (1,321)	> 10,000	> 10,000
**1k**	393 (354)	1,782 (1,782)	> 10,000	> 10,000
GNF 5837[b]	177	> 10,000	> 10,000	> 10,000
AZ Dyrk1B 33[c]	> 10,000	> 10,000	14 (28)	50 (50)
Harmine[d]	> 10,000	> 10,000	66 9132)	85 (85)
LH 846[e]	> 10,000	80	> 10,000	> 10,000
TC-S 7004[f]	> 10,000	> 10,000	< 10	22 (22)

[a] IC_{50} values were determined using an 11-point 3-fold serial dilution of each test compound and K_i was determined by Cheng-Prusoff equation; [b] Selective pan-TRK inhibitor; [c] Selective Dyrk1B kinase inhibitor; [d] DYRK1A kinase inhibitor; [e] potent inhibitor of CK1δ; [f] Potent and selective DYRK1A/B inhibitor.
